# Supplementary material for: Synthesis of New Highly Functionalized 1H-Indole-2-carbonitriles via Cross-Coupling Reactions
Source: Molecules. 2021 Aug 31;26(17):5287. doi: 10.3390/molecules26175287 (PMC8434198; doi:10.3390/molecules26175287)

SUPPORTING INFORMATION

Table of Contents

1. ^{1}H NMR and J-mod spectra of compounds **3** *page 2-6*
2. ^{1}H NMR and ^{13}C NMR spectra of compound **4** *page 7*
3. ^{1}H NMR and ^{13}C NMR spectra of compounds **5** *page 8-13*
4. ^{1}H NMR and ^{13}C NMR spectra of compounds **6** *page 14-18*
5. ^{1}H NMR and ^{13}C NMR spectra or J-mod experiment of compounds **7** *page 19-23*
6. ^{1}H NMR and ^{13}C NMR spectra or J-mod experiment of compounds **8** *page 24-36*
7. ^{1}H NMR and ^{13}C NMR spectra or J-mod experiment of of compounds **9** *page 37-56*
8. ^{1}H NMR and ^{13}C NMR spectra of compounds **10** *page 57-63*
9. ^{1}H NMR and ^{13}C NMR spectra of compounds **11** *page 64-65*
10. ^{1}H NMR and ^{13}C NMR spectra of compound **8b’** *page 66*

1*H*-Indole-2-carbonitrile (3a)

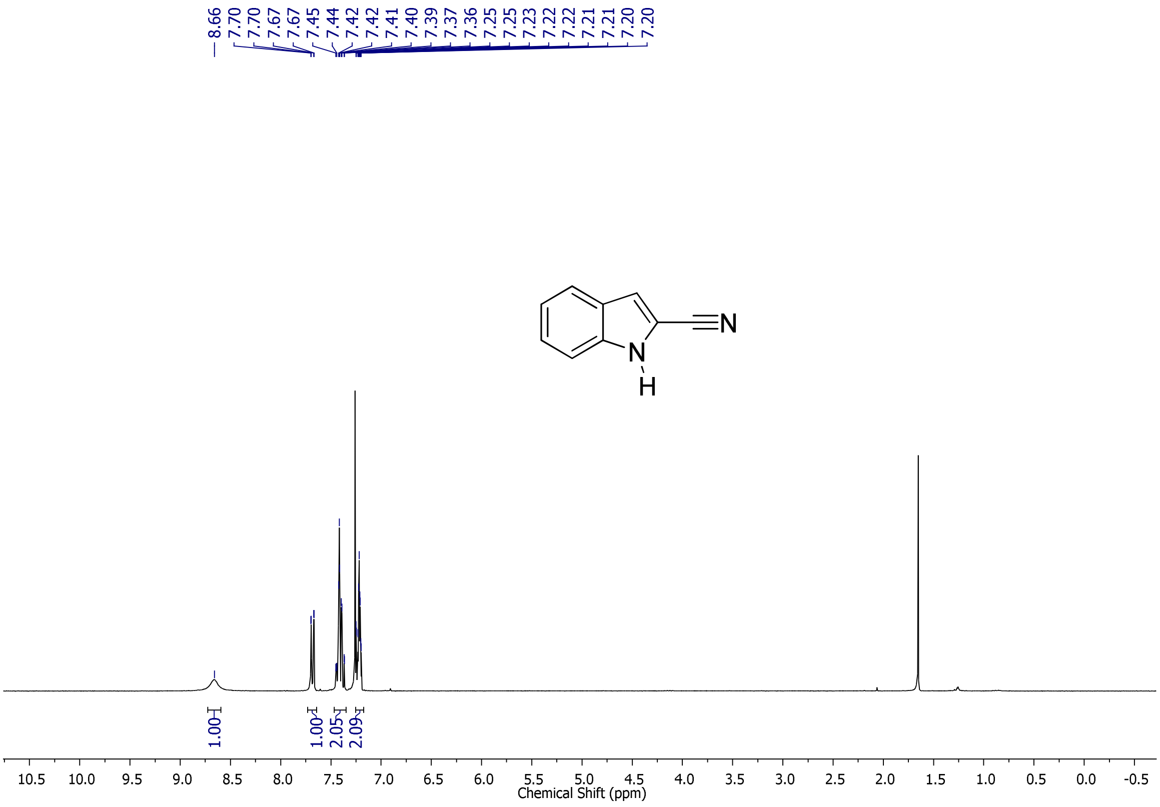

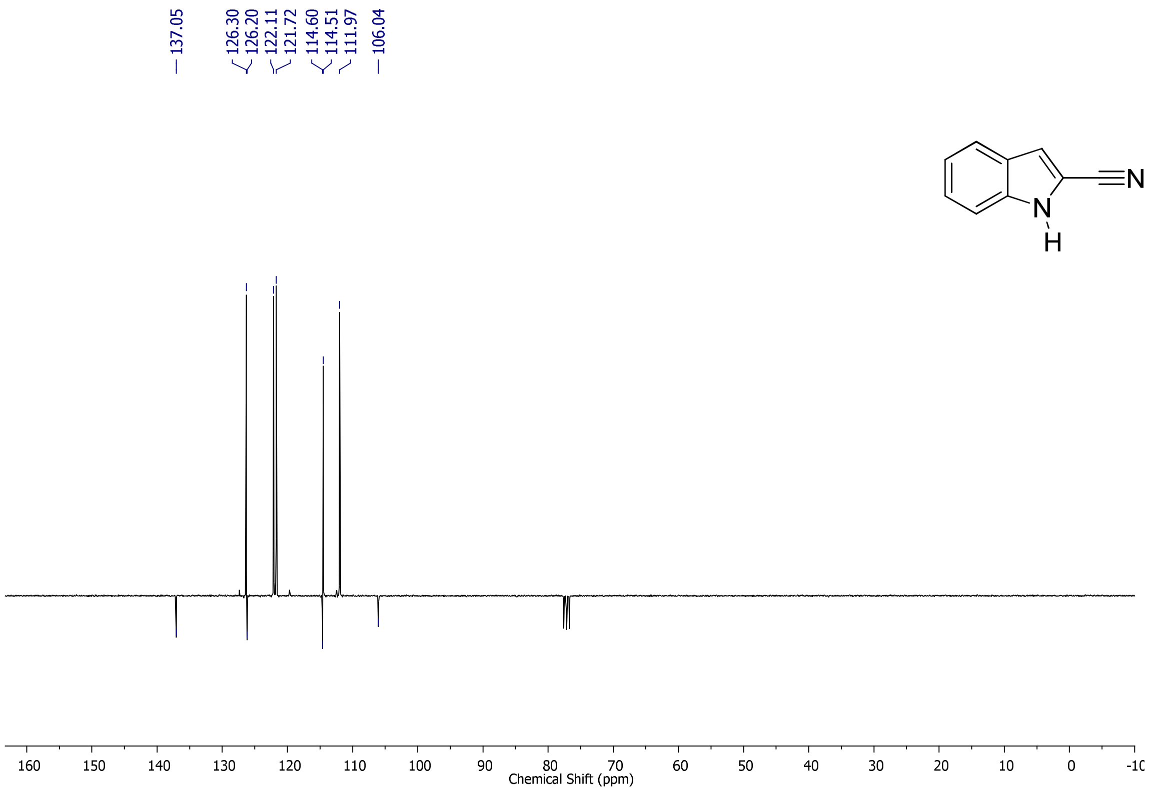

5-Methoxy-1*H*-indole-2-carbonitrile (3b)

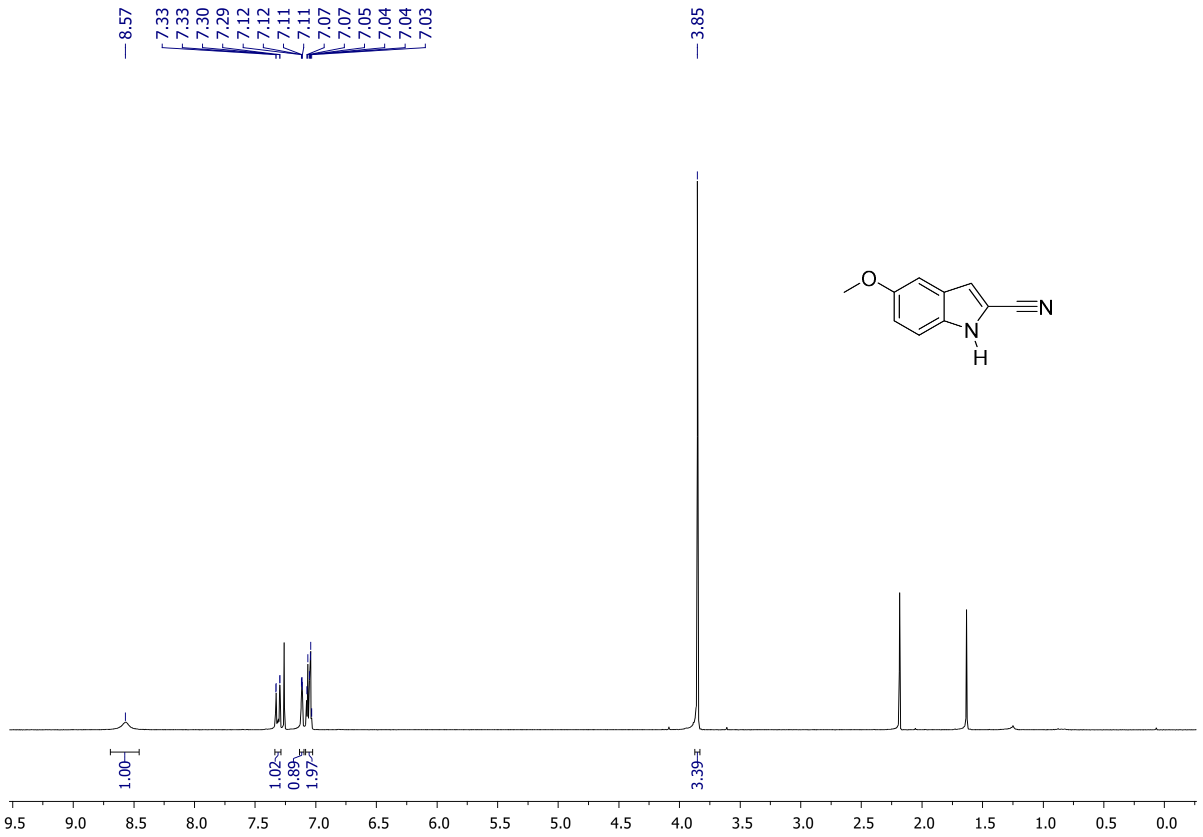

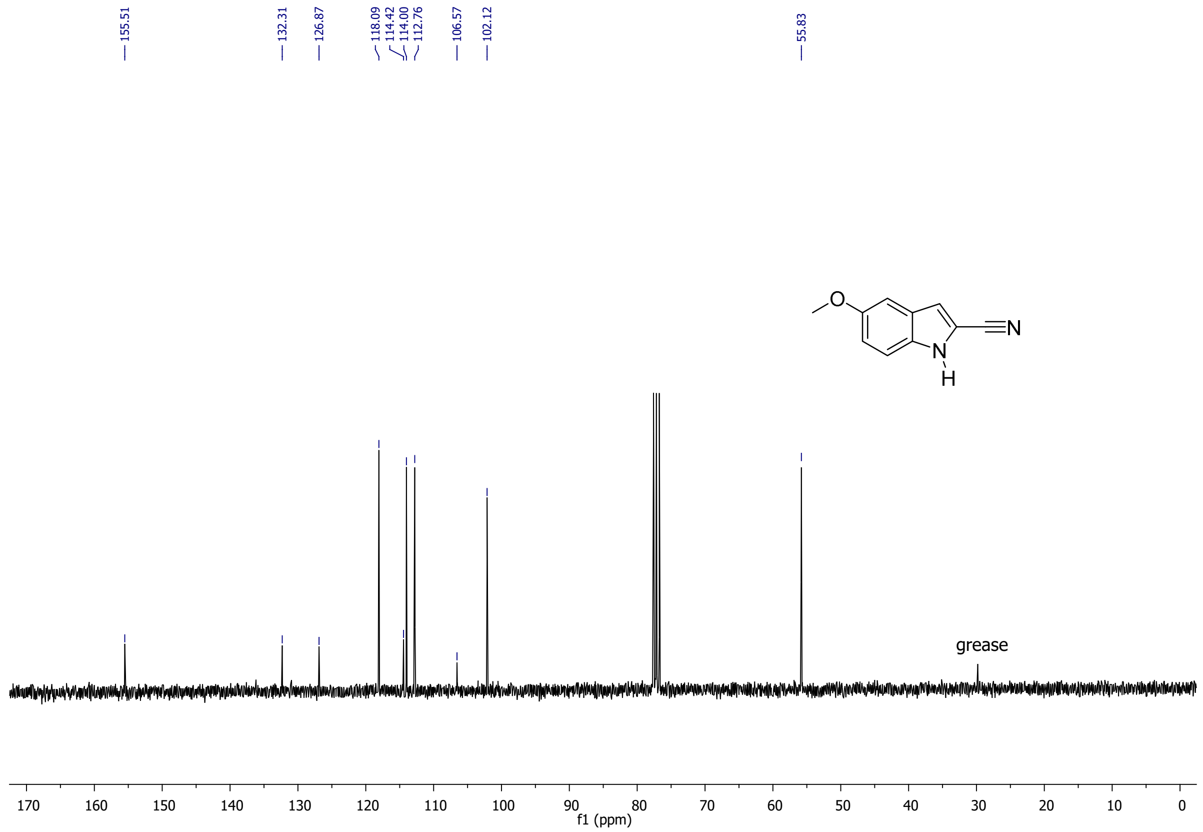

6-Methoxy-1*H*-indole-2-carbonitrile (3c)

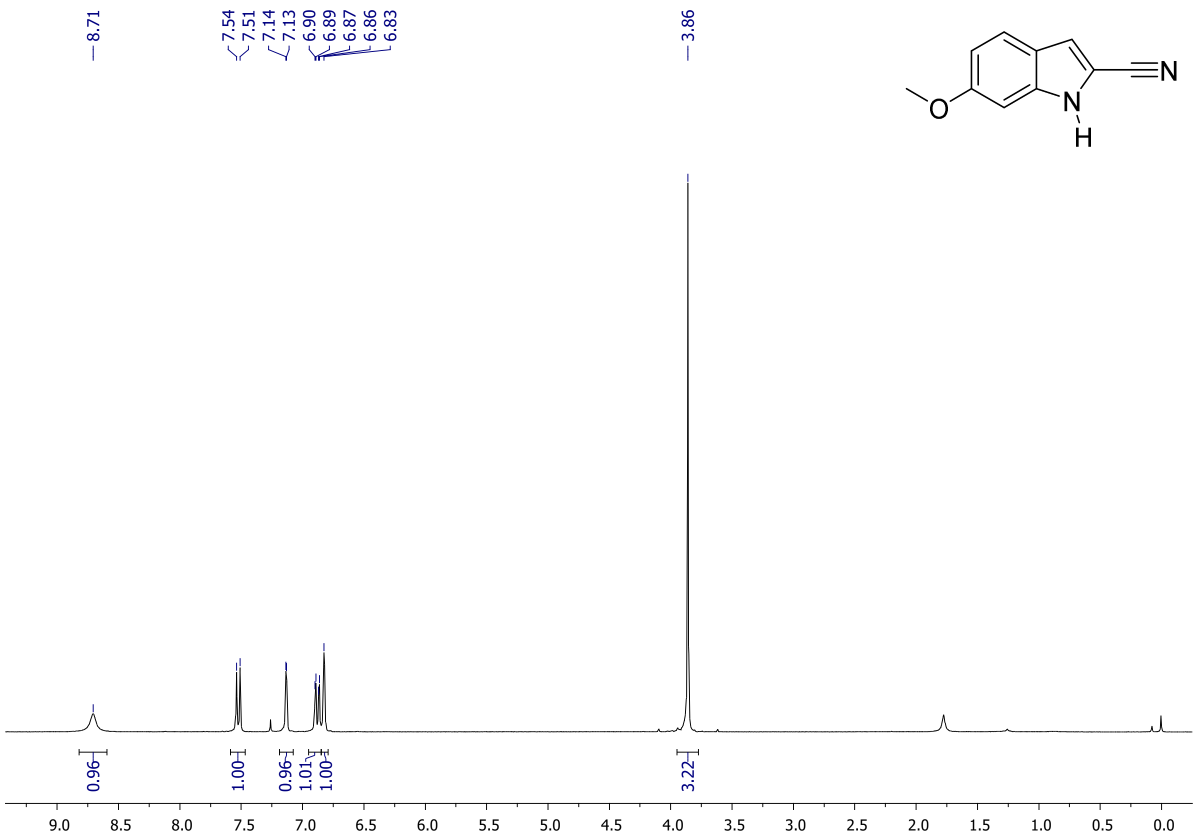

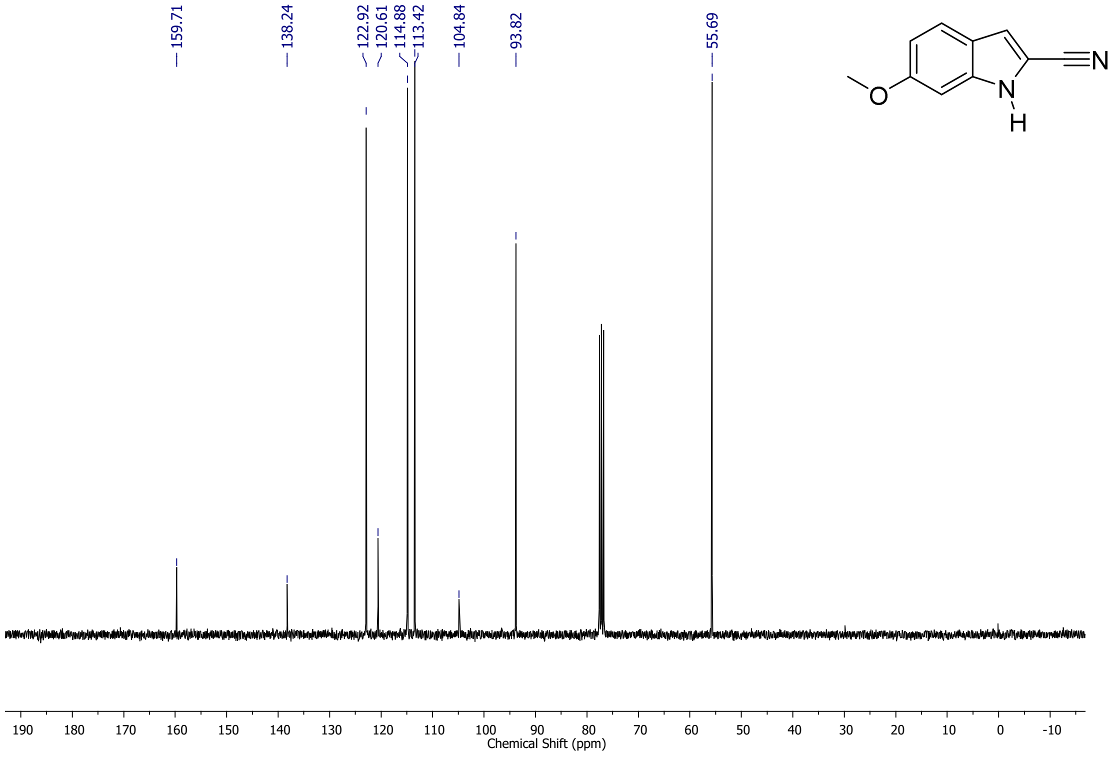

5-Fluoro-1*H*-indole-2-carbonitrile (3d)

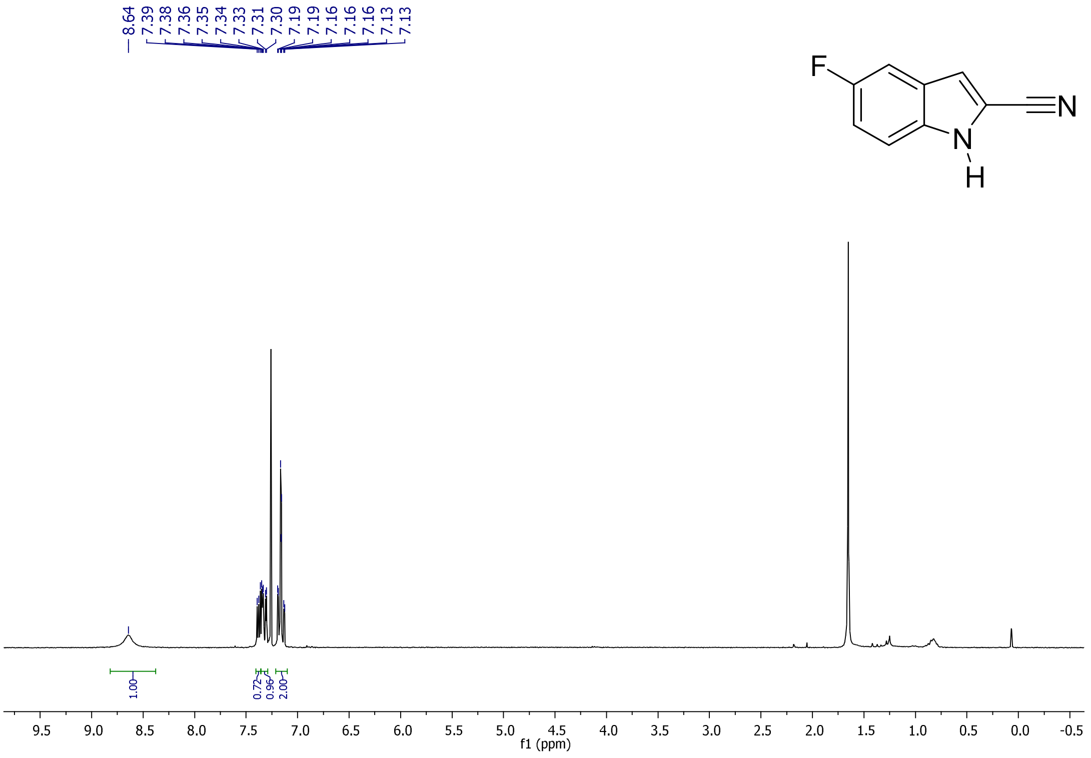

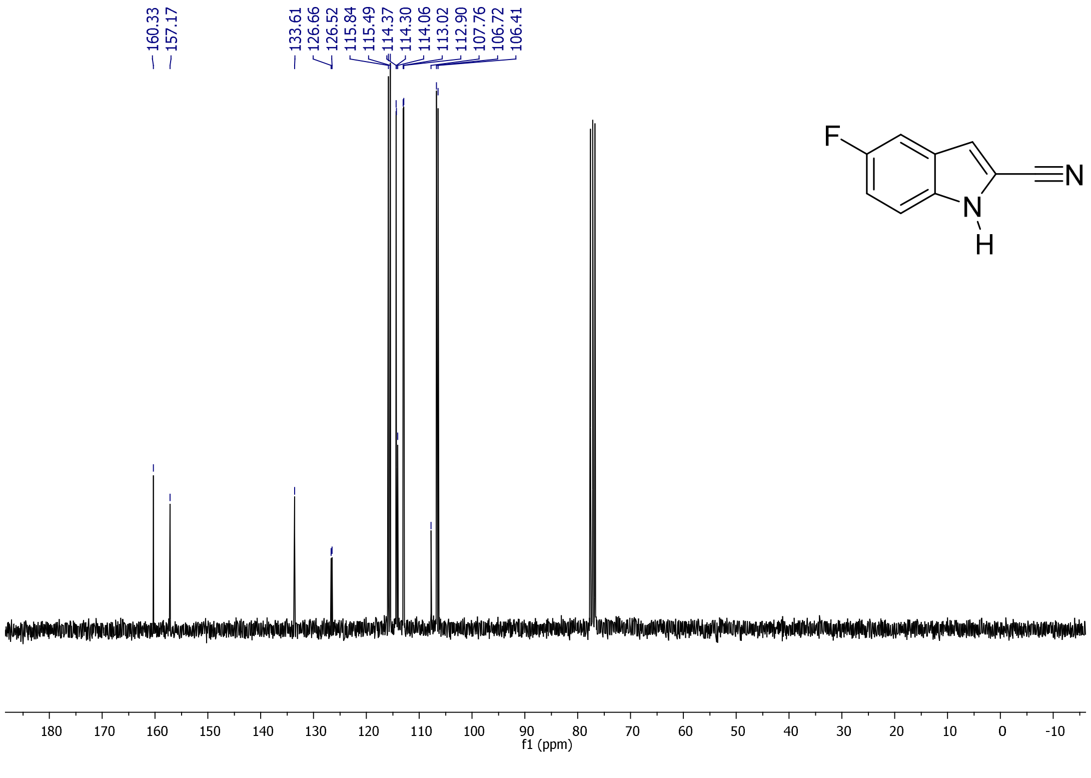

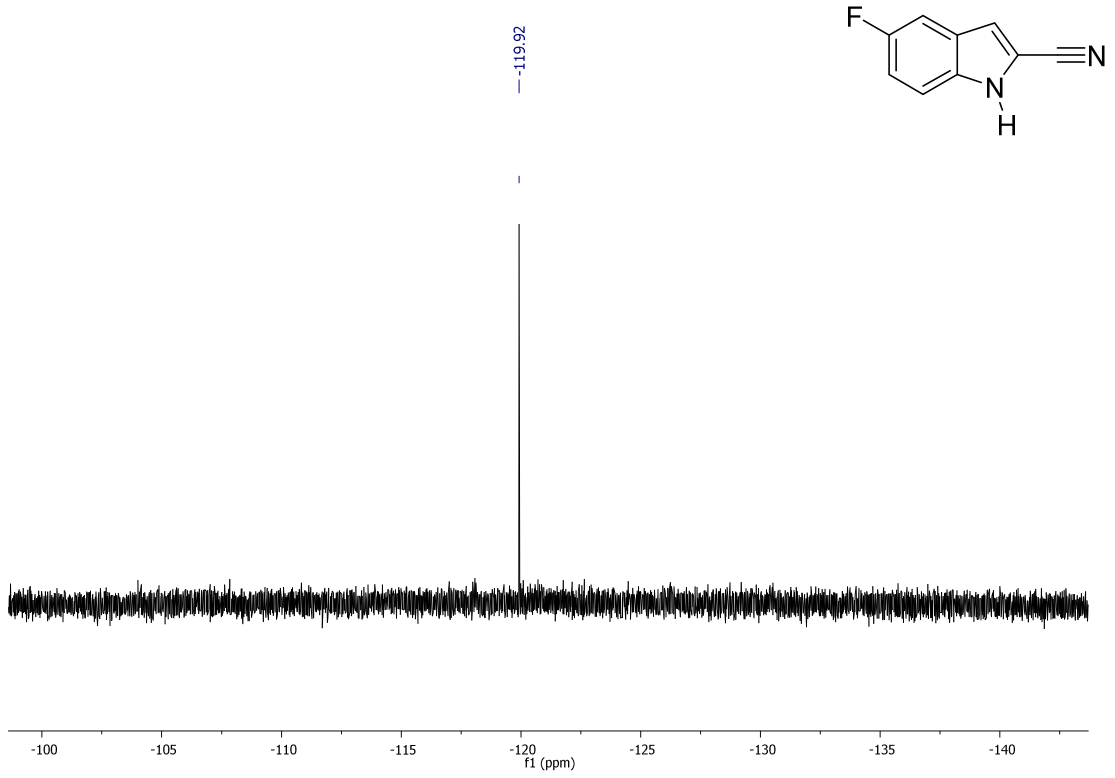

-119.92
F
N
H
N
-100
-105
-110
-115
-120
-125
-130
-135
-140
f1 (ppm)

1-(Prop-2-yn-1-yl)-1*H*-indole-2-carbonitrile (4a)

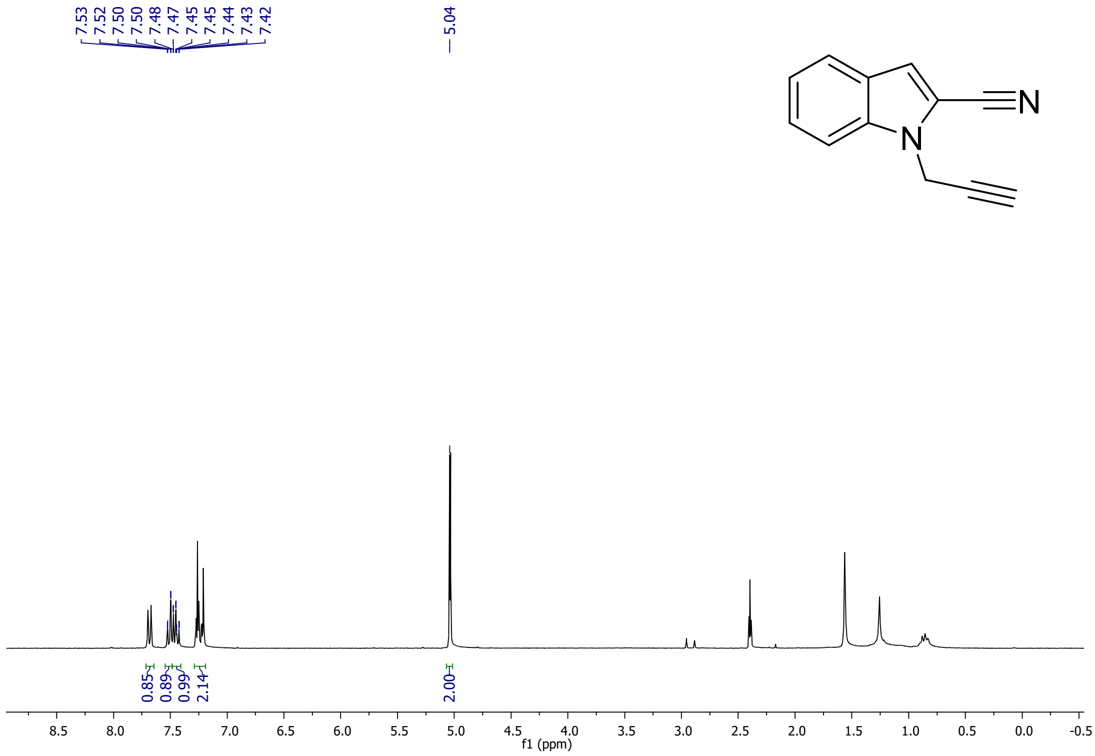

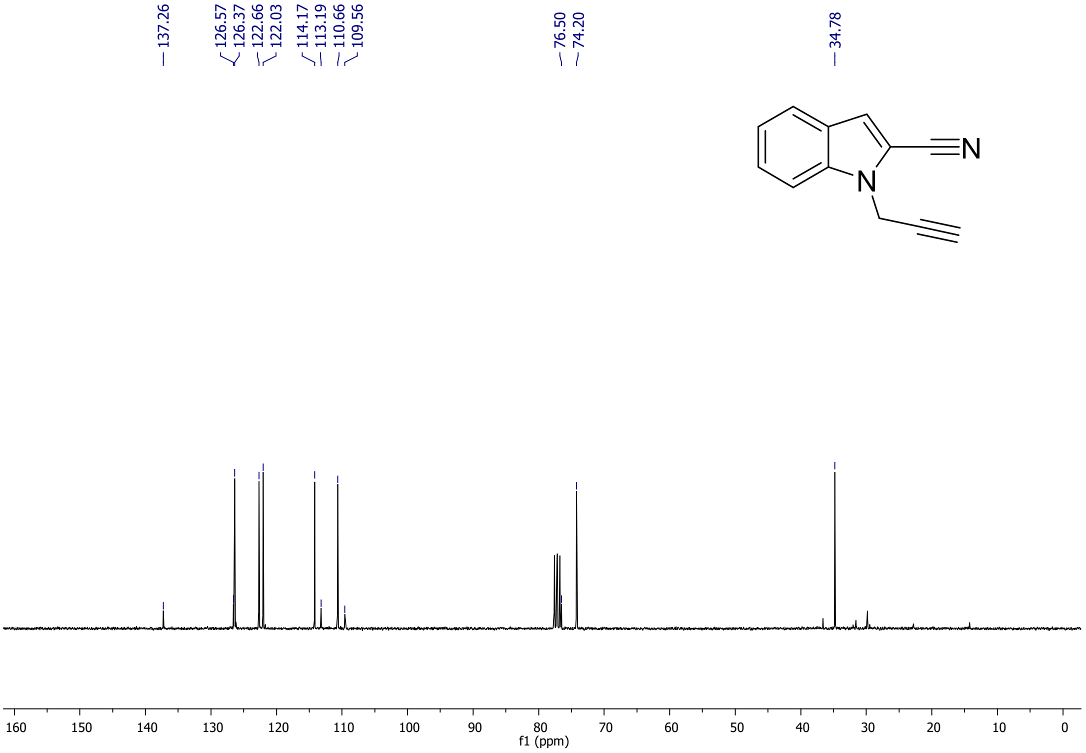

1-(3-(*p*-Tolyl)prop-2-yn-1-yl)-1*H*-indole-2-carbonitrile (5a)

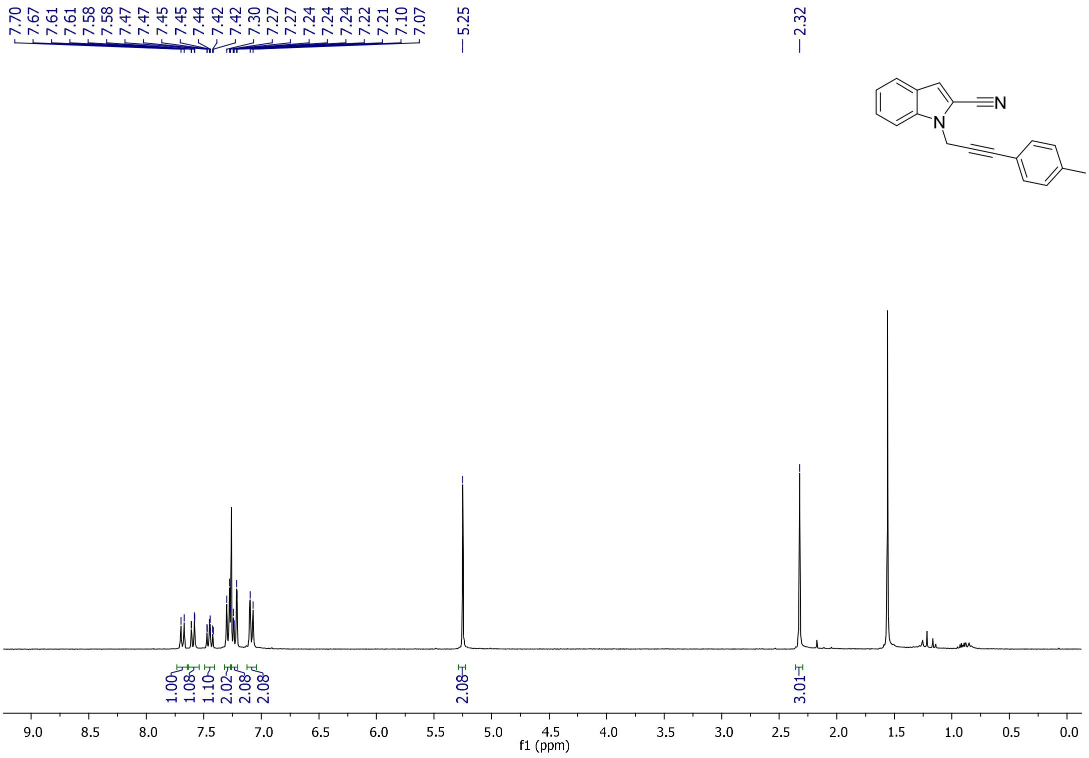

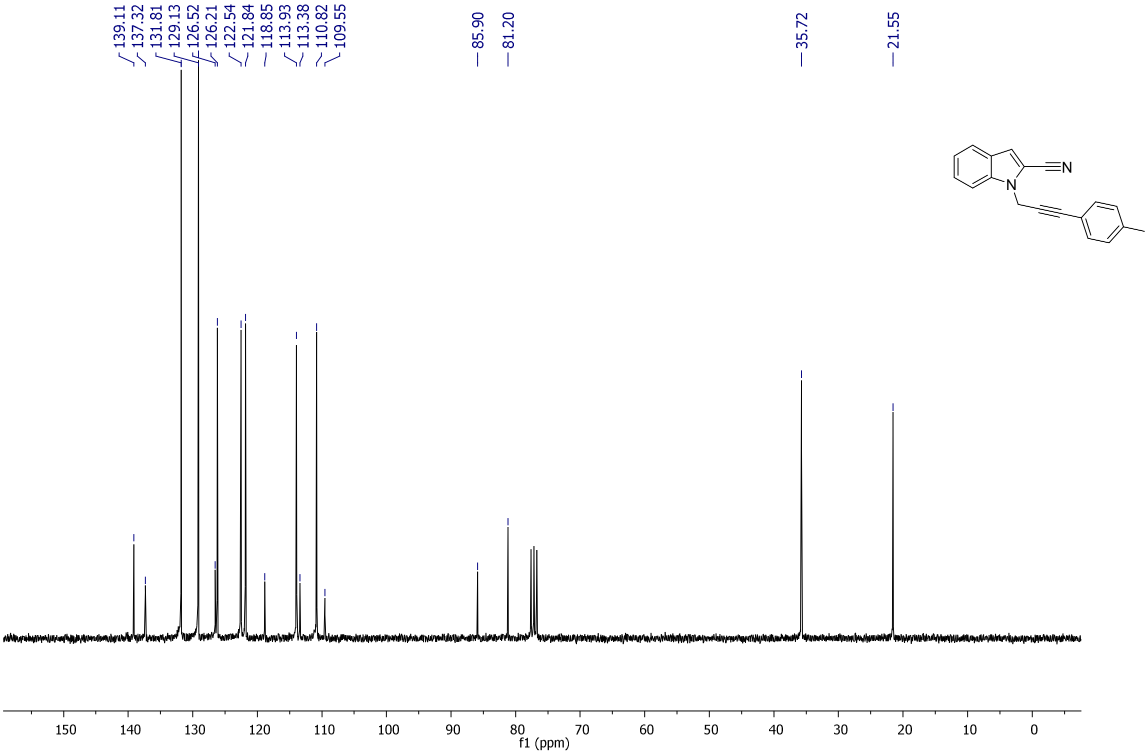

1-(3-(4-Chlorophenyl)prop-2-yn-1-yl)-1*H*-indole-2-carbonitrile (5b)

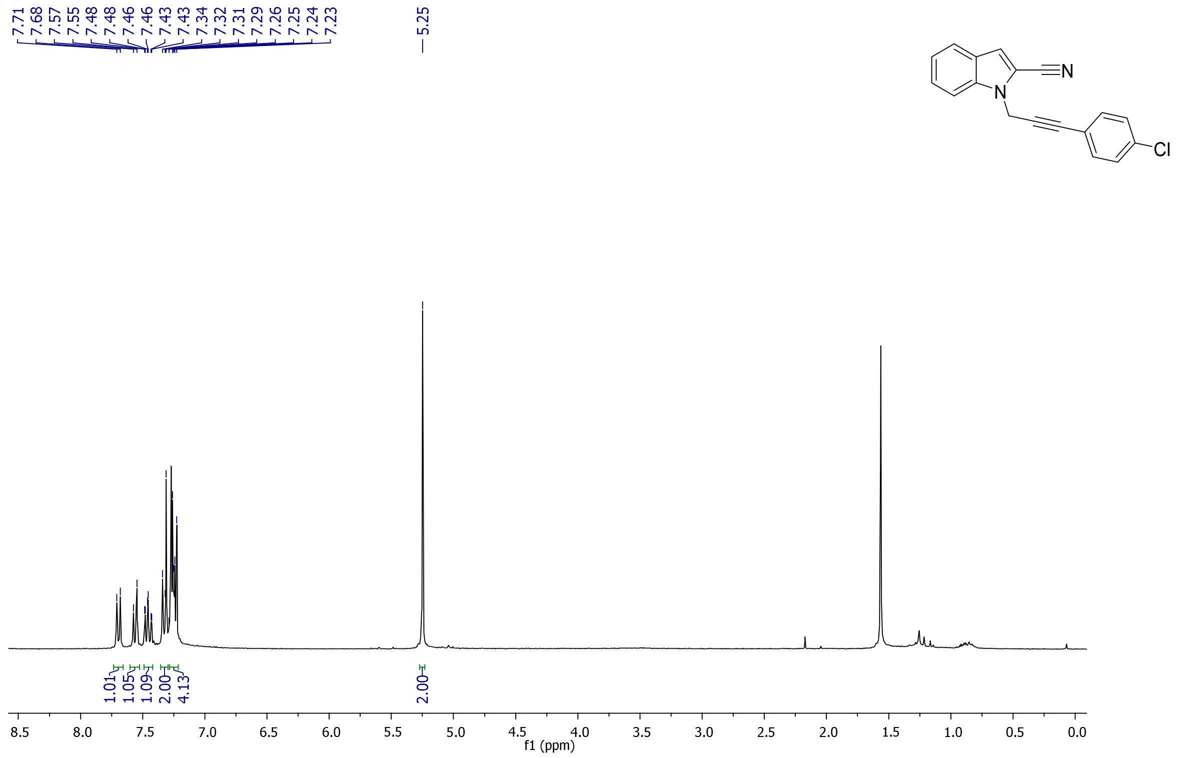

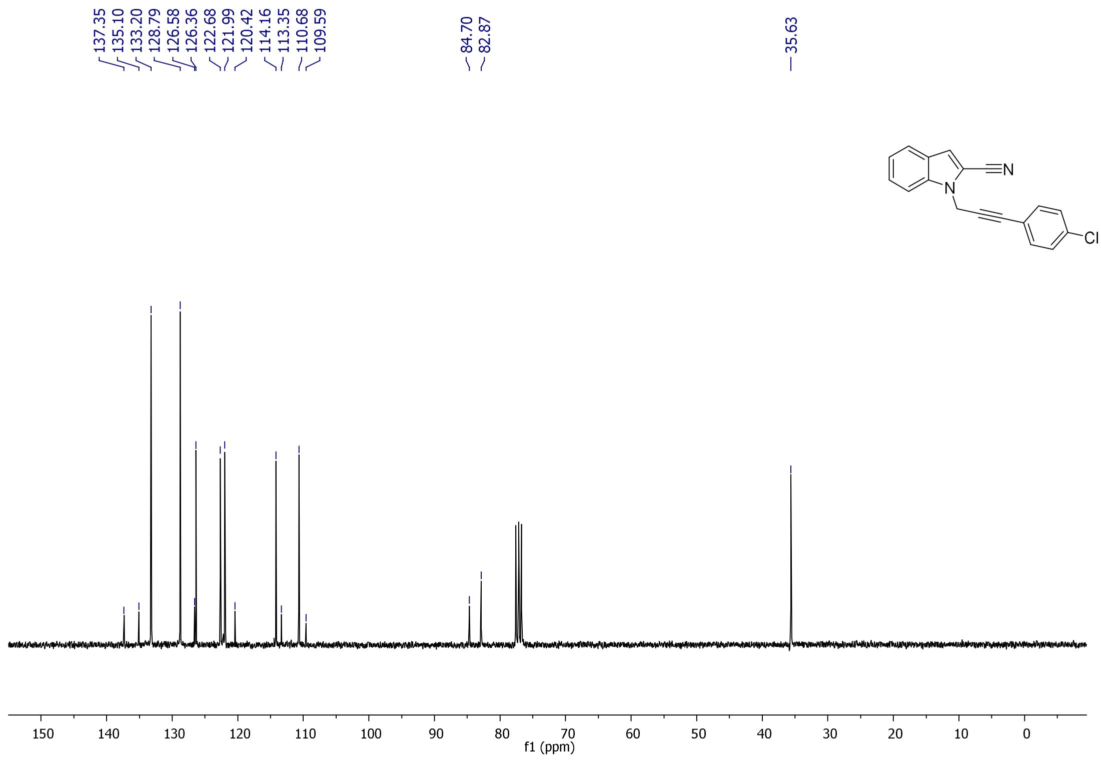

1-(3-(4-(Trifluoromethyl)phenyl)prop-2-yn-1-yl)-1*H*-indole-2-carbonitrile (5c)

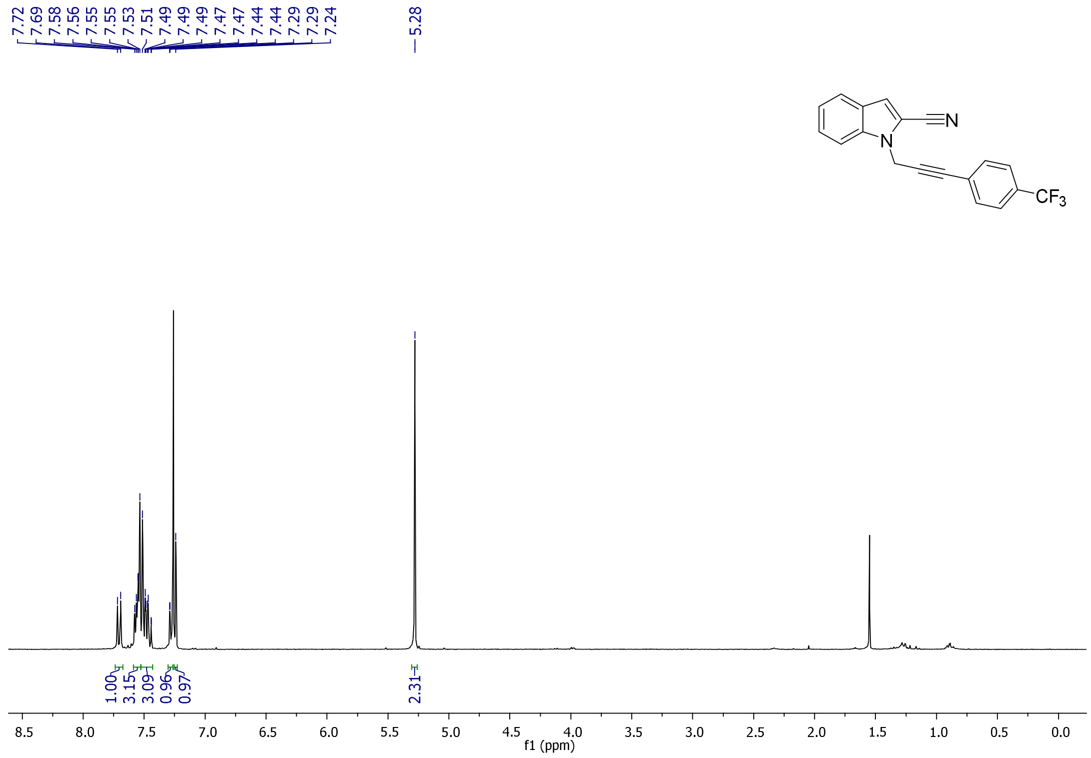

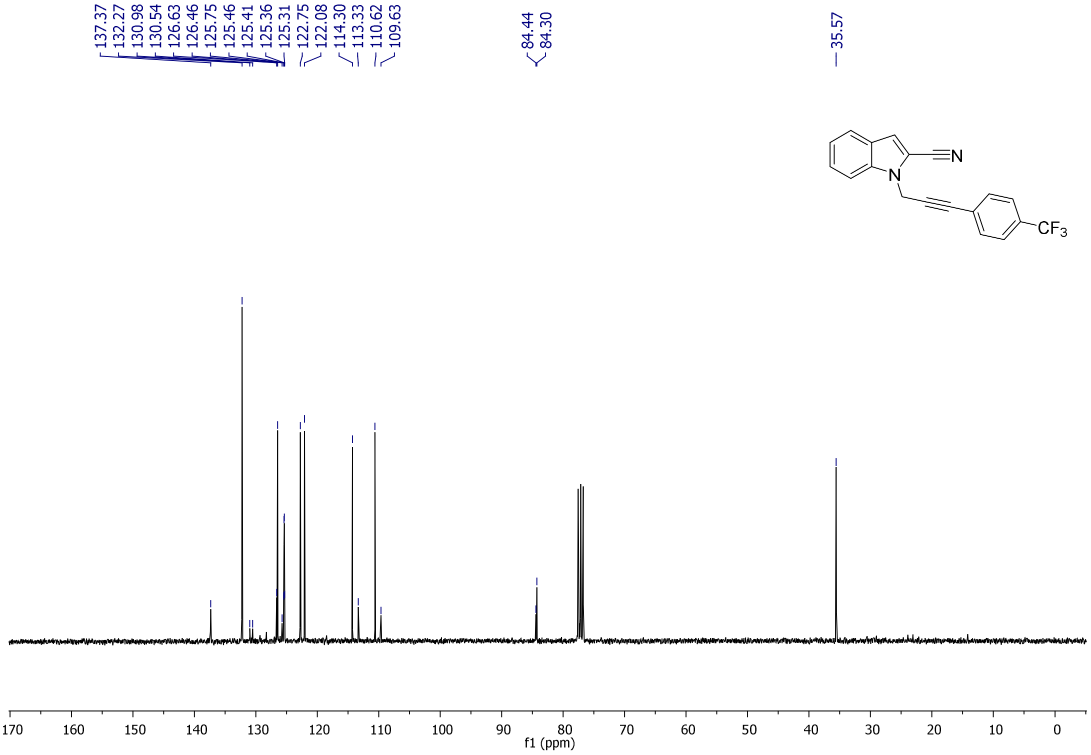

1-(3-(2-Cyanophenyl)prop-2-yn-1-yl)-1*H*-indole-2-carbonitrile (5d)

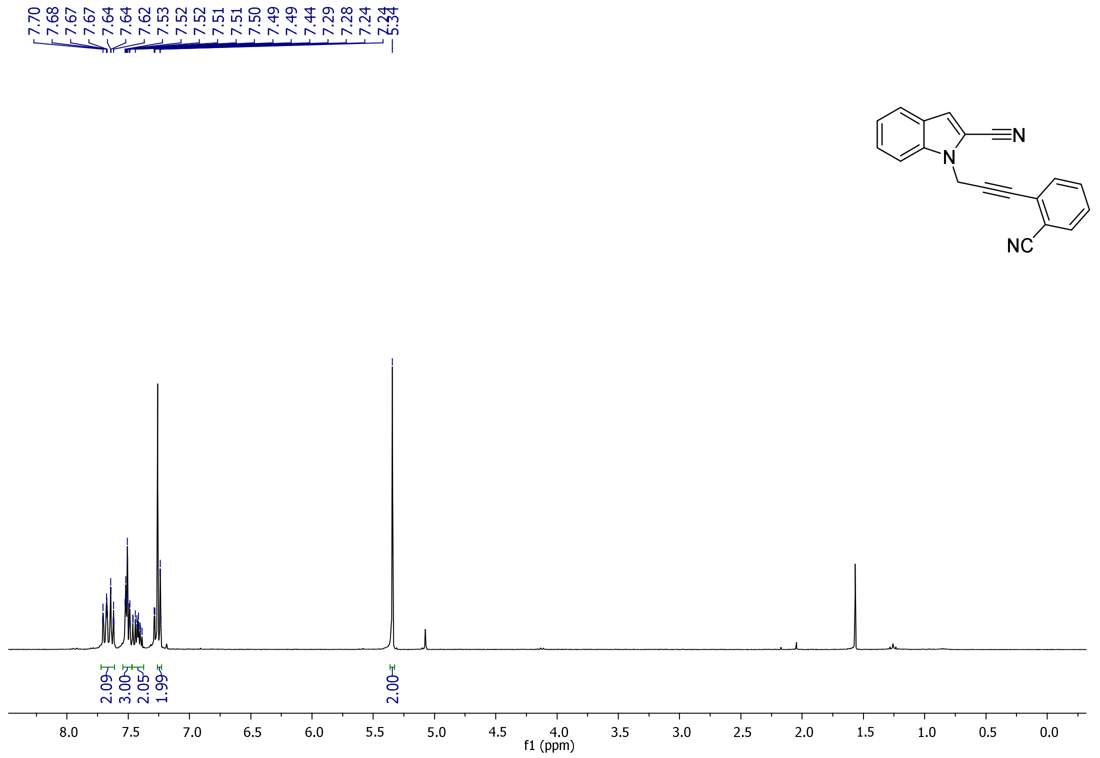

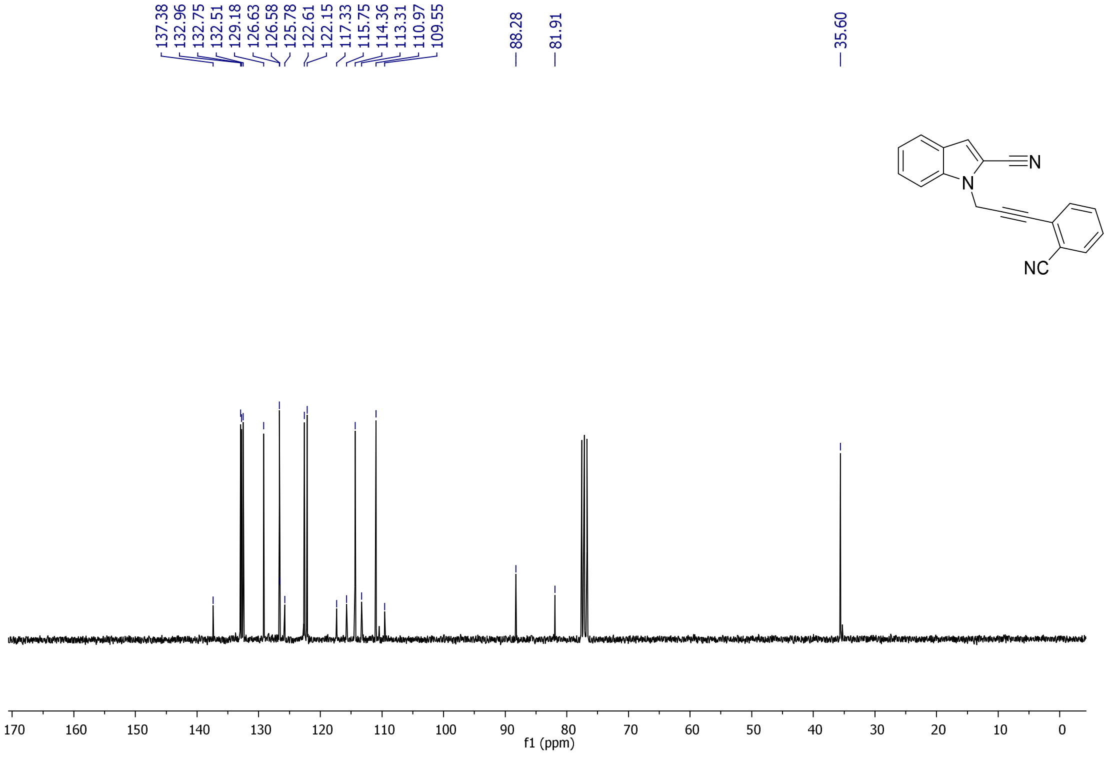

1-(3-(2-Methoxyphenyl)prop-2-yn-1-yl)-1*H*-indole-2-carbonitrile (5e)

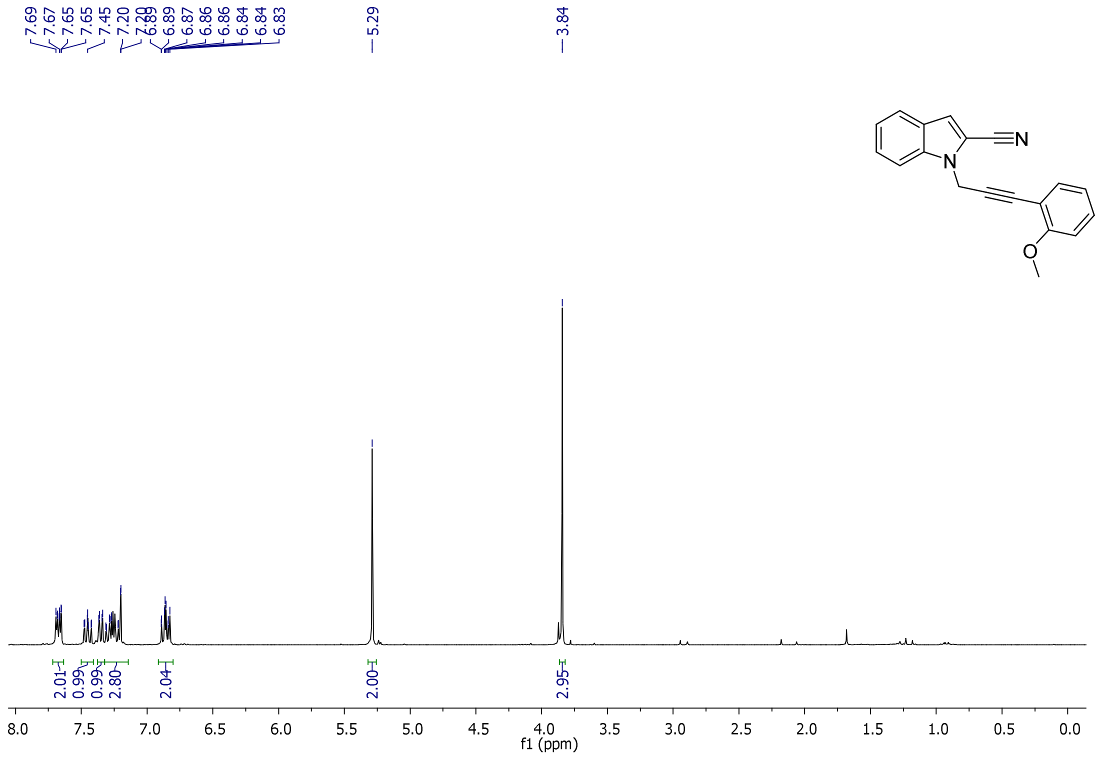

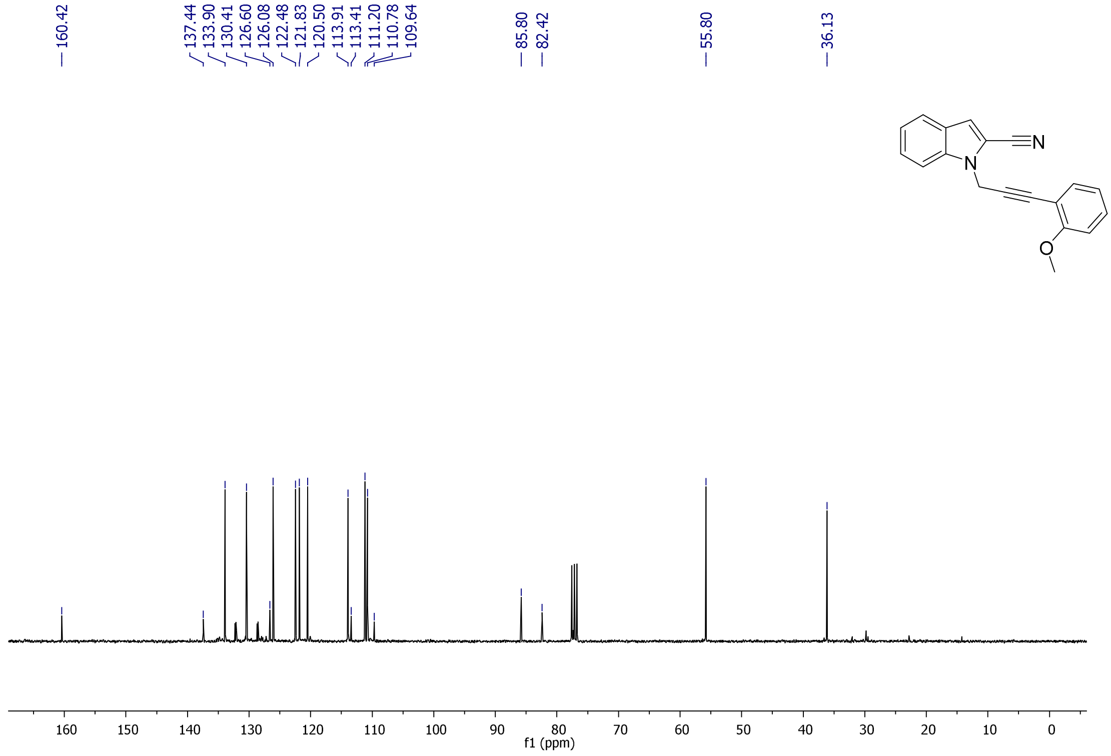

1-(3-(3,4-Dichlorophenyl)prop-2-yn-1-yl)-1*H*-indole-2-carbonitrile (5f)

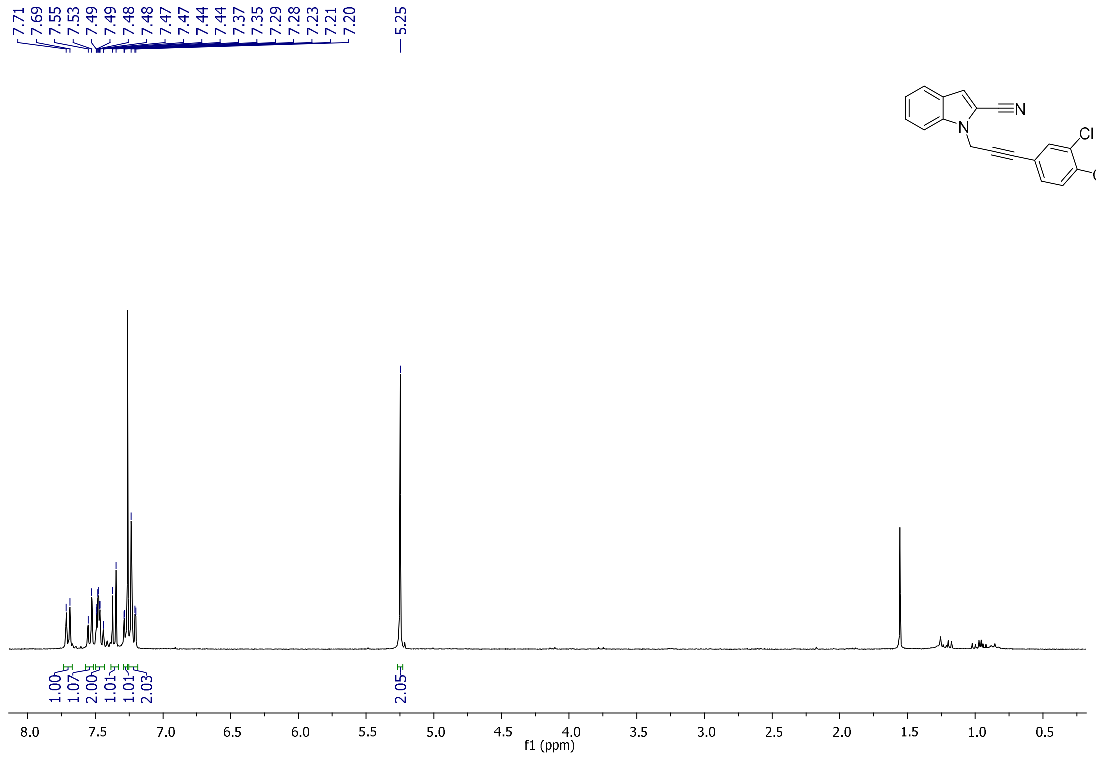

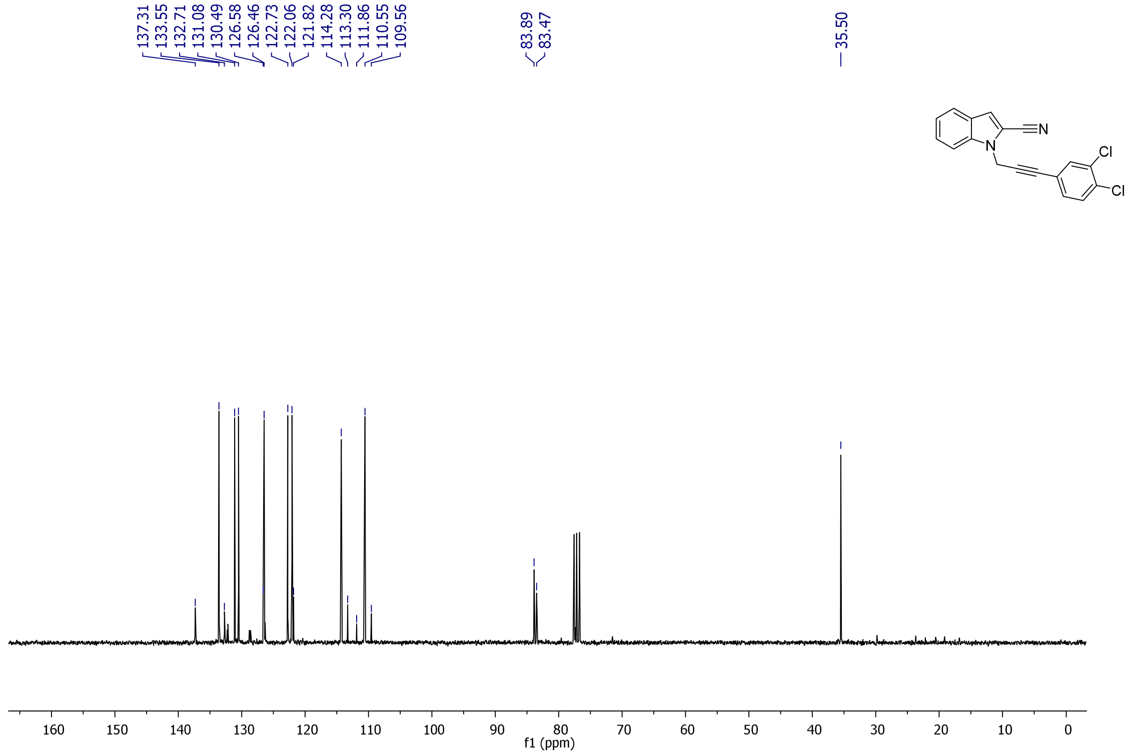

3-Iodo-1*H*-indole-2-carbonitrile (6a)

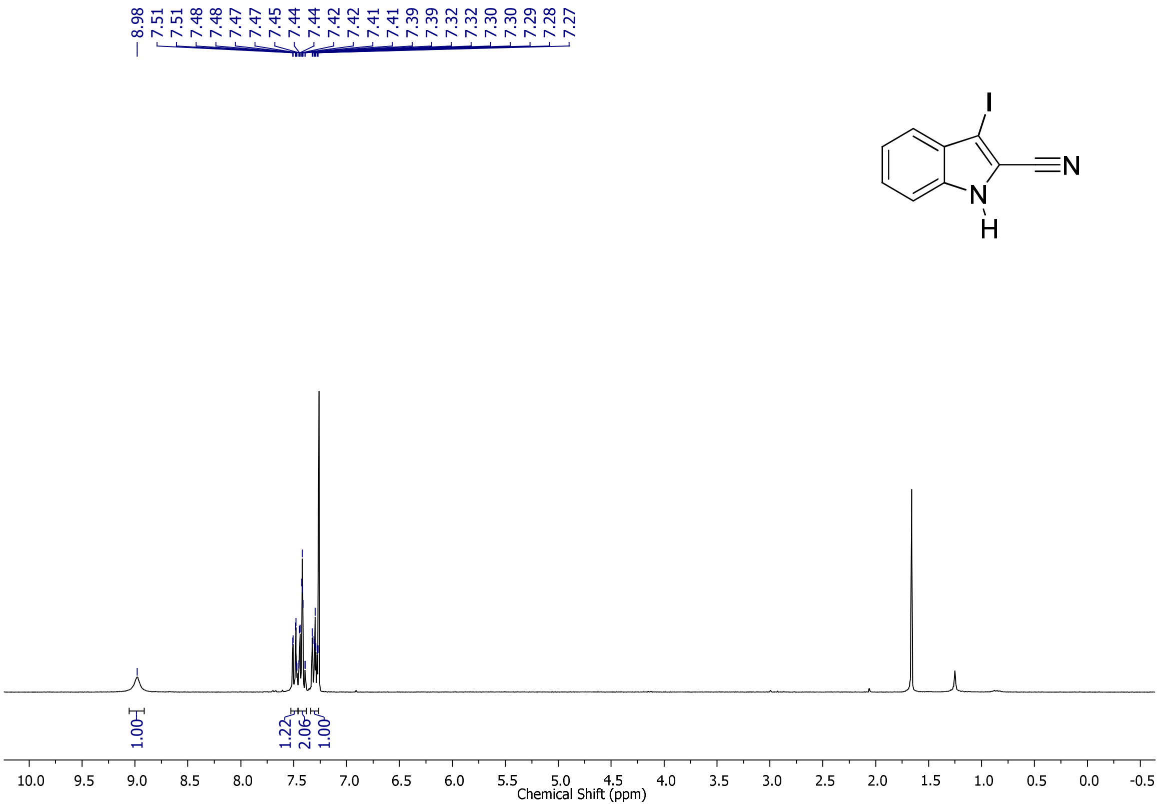

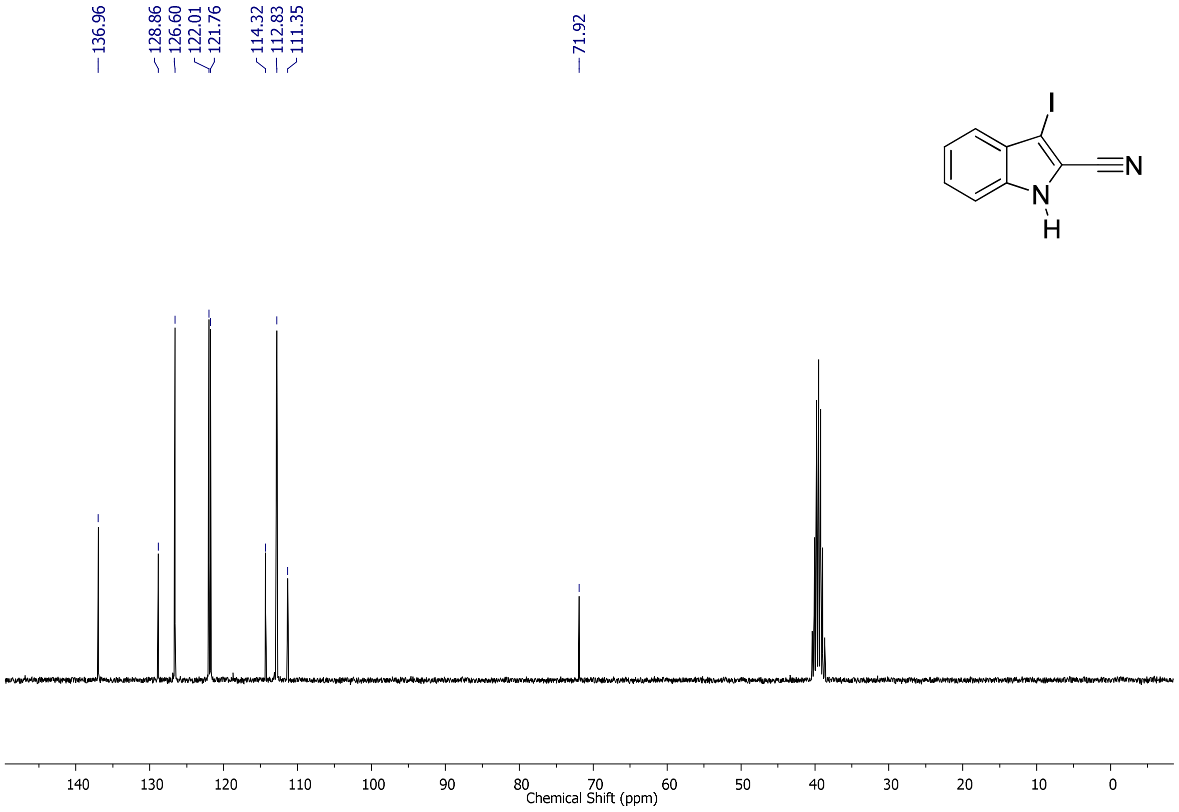

3-Iodo-5-methoxy-1*H*-indole-2-carbonitrile (6b)

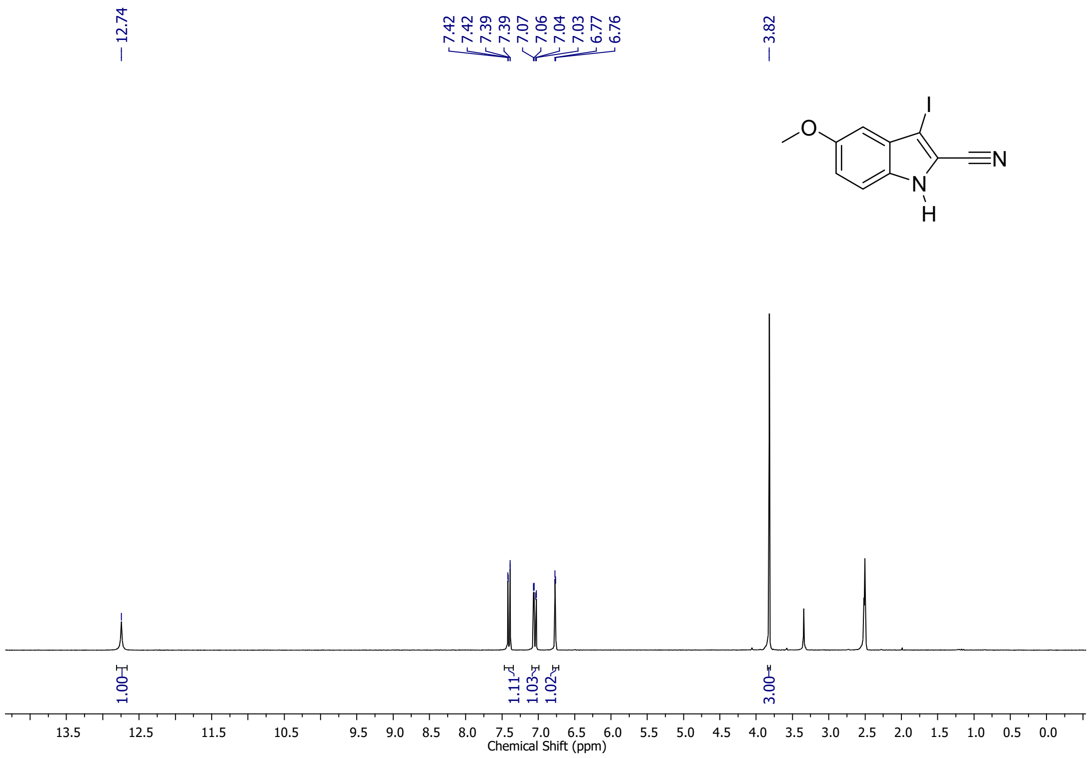

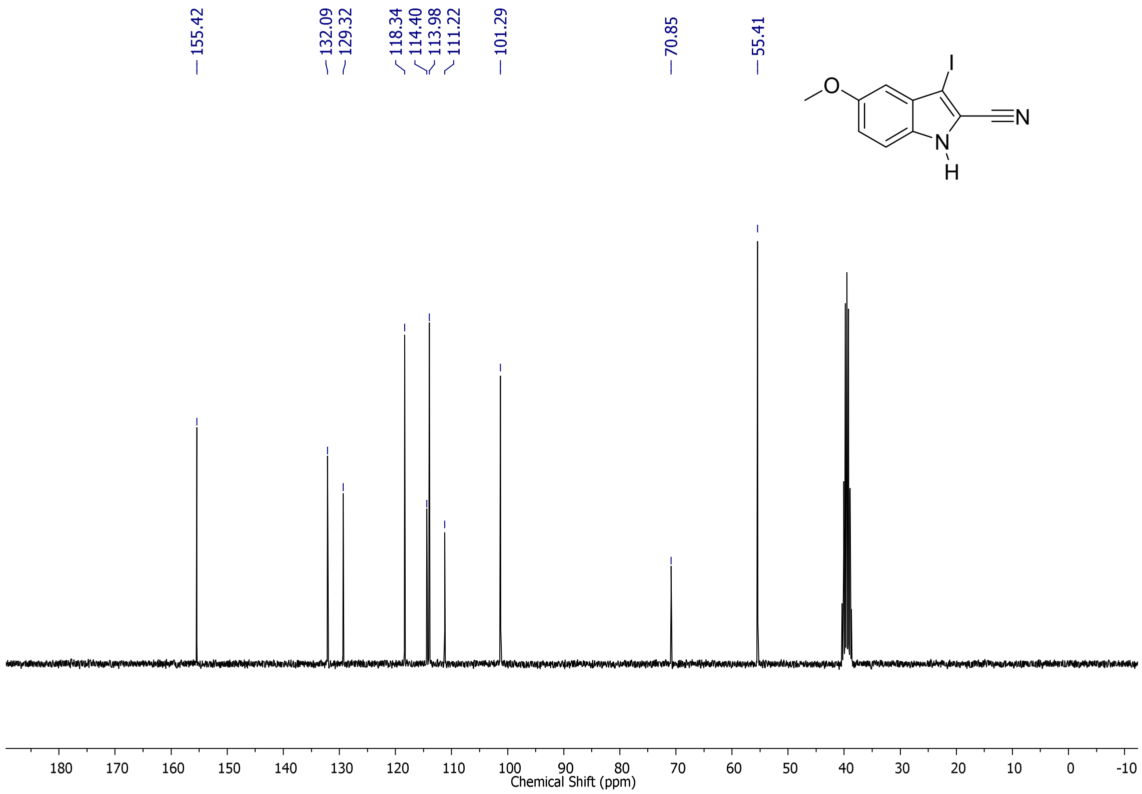

3-Iodo-6-methoxy-1*H*-indole-2-carbonitrile (6c)

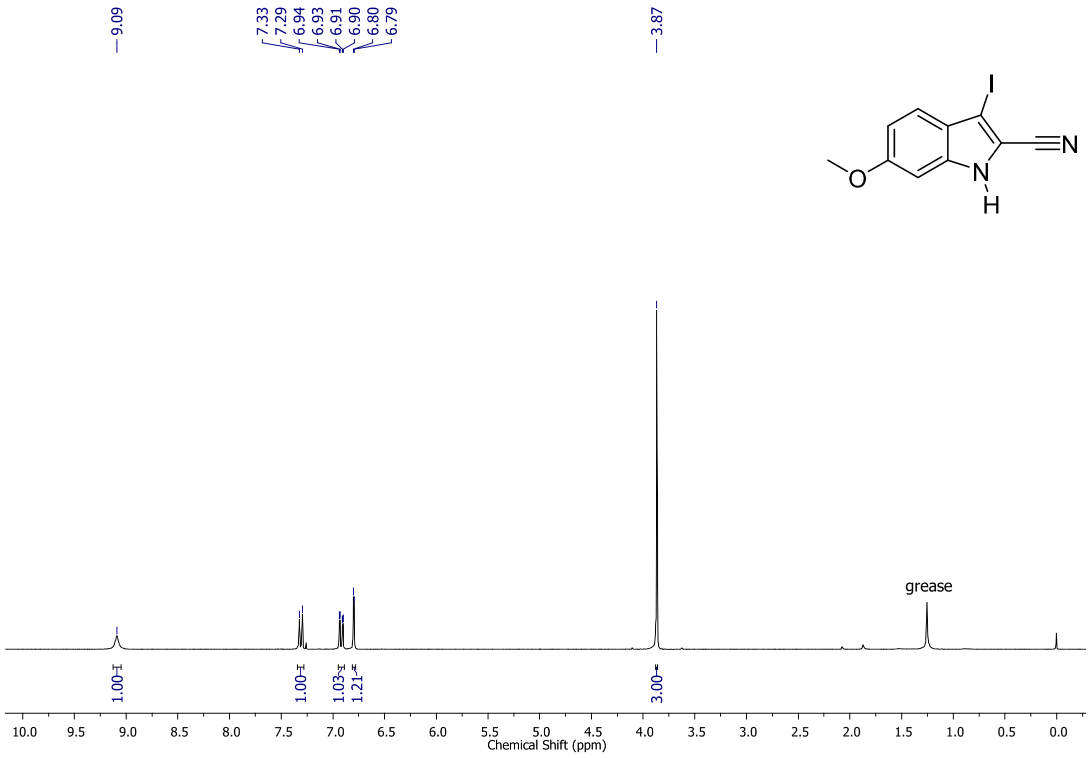

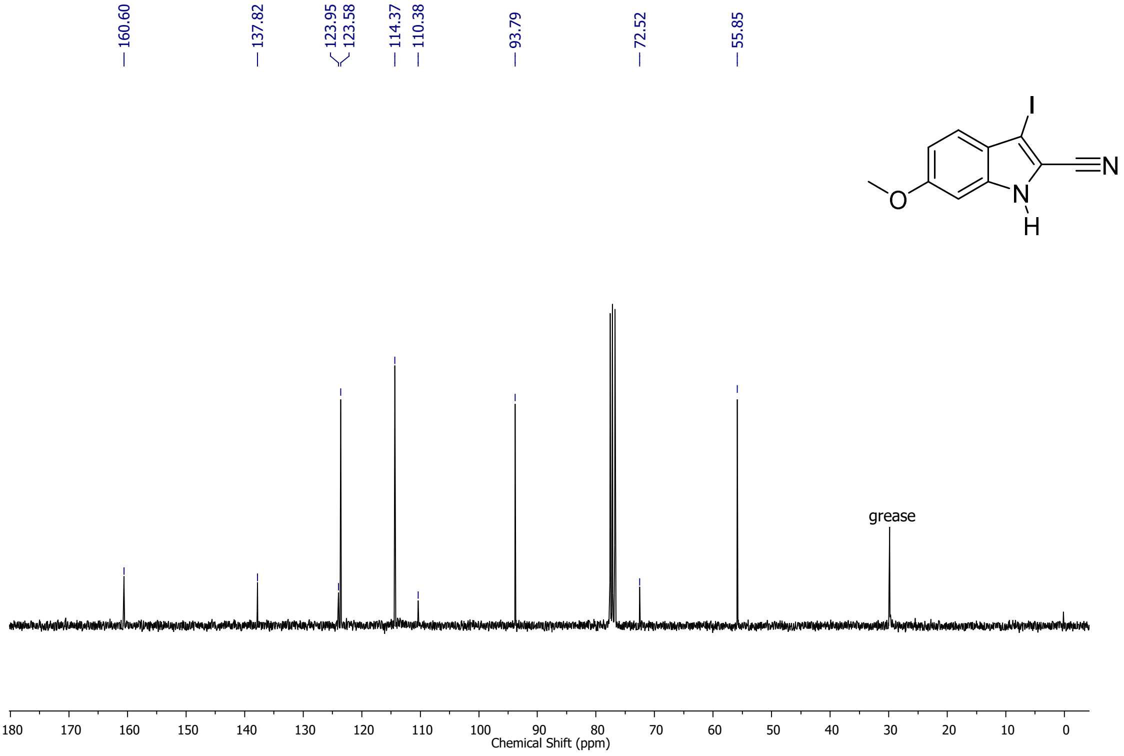

5-Fluoro-3-iodo-1*H*-indole-2-carbonitrile (6d)

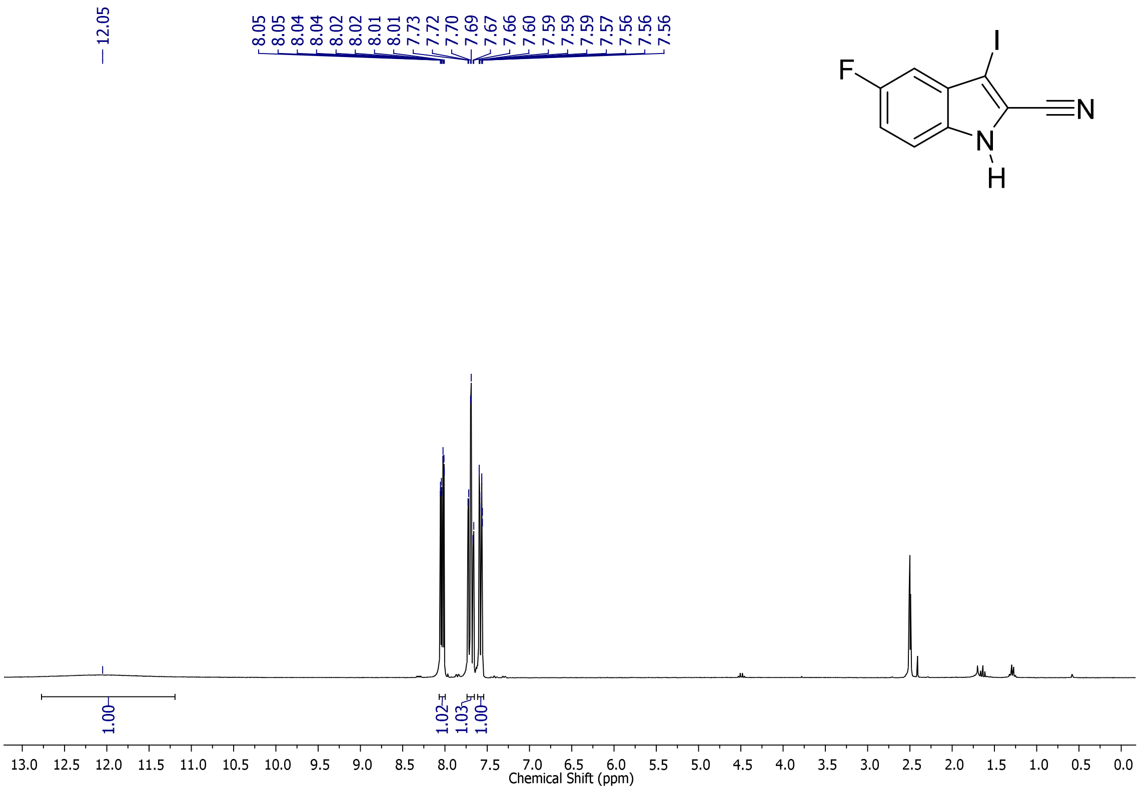

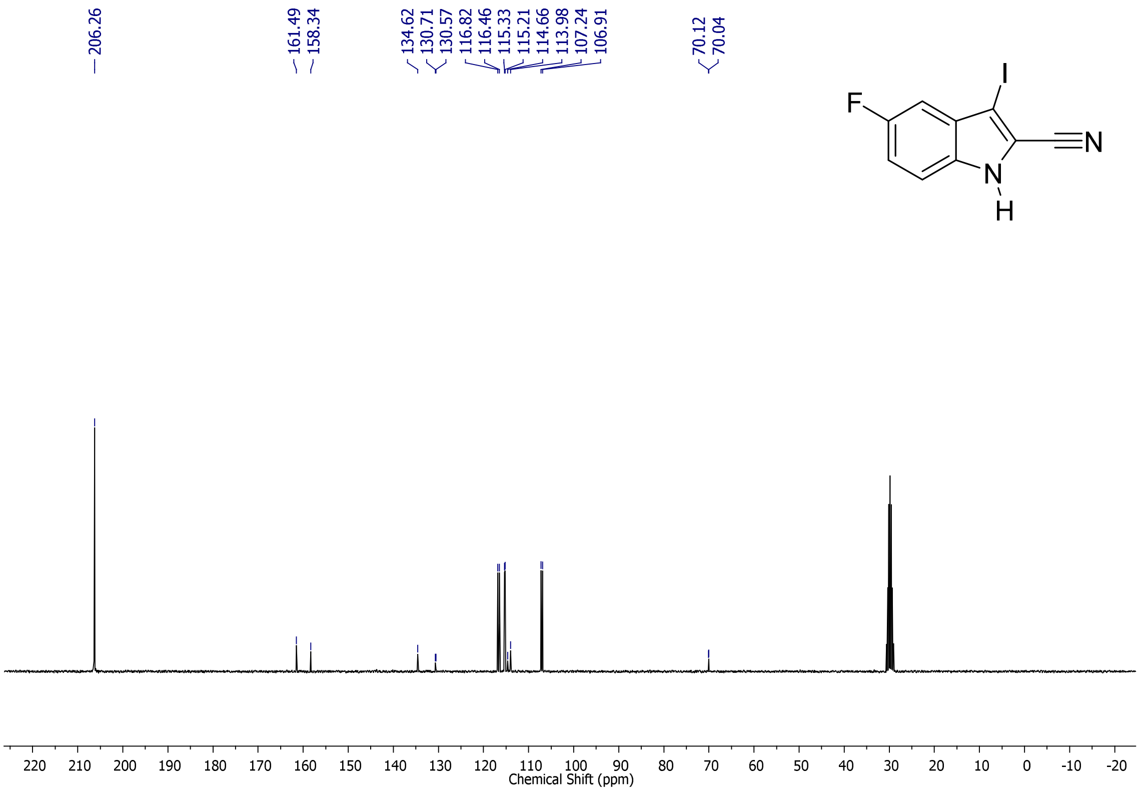

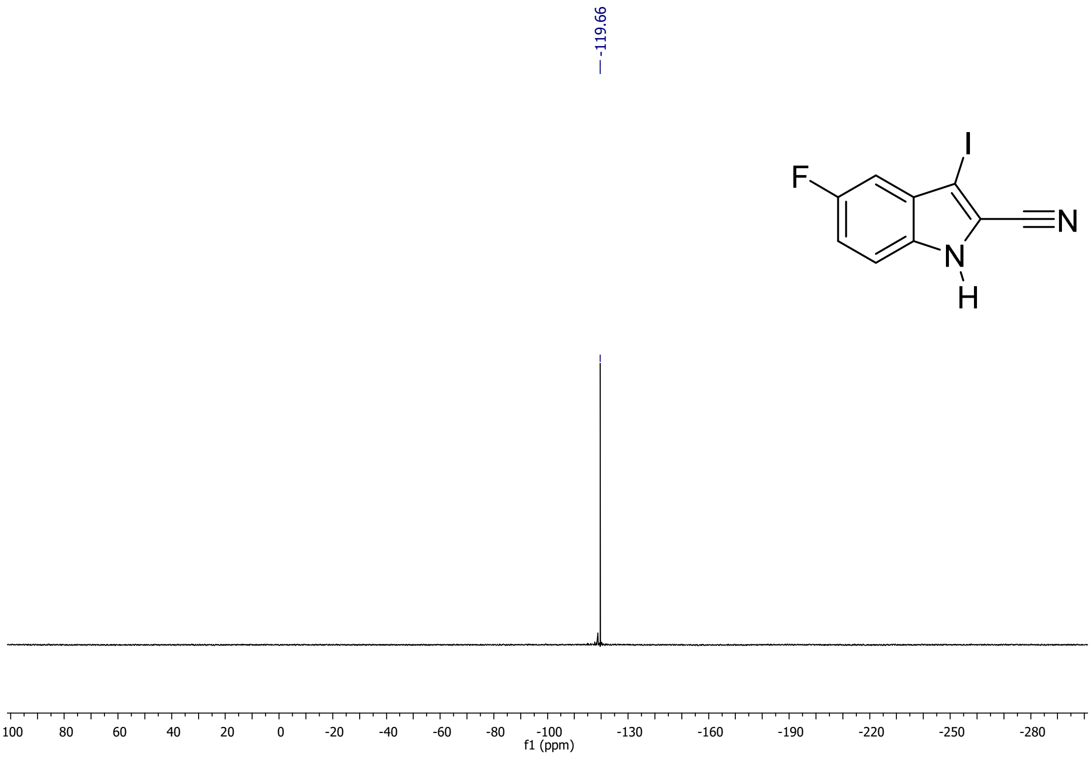

-119.66
F
I
N
N
H
100
80
60
40
20
0
-20
-40
-60
-80
-100
-130
-160
-190
-220
-250
-280
f1 (ppm)

1-Benzyl-3-Iodo-1*H*-Indole-2-carboxamide (7a)

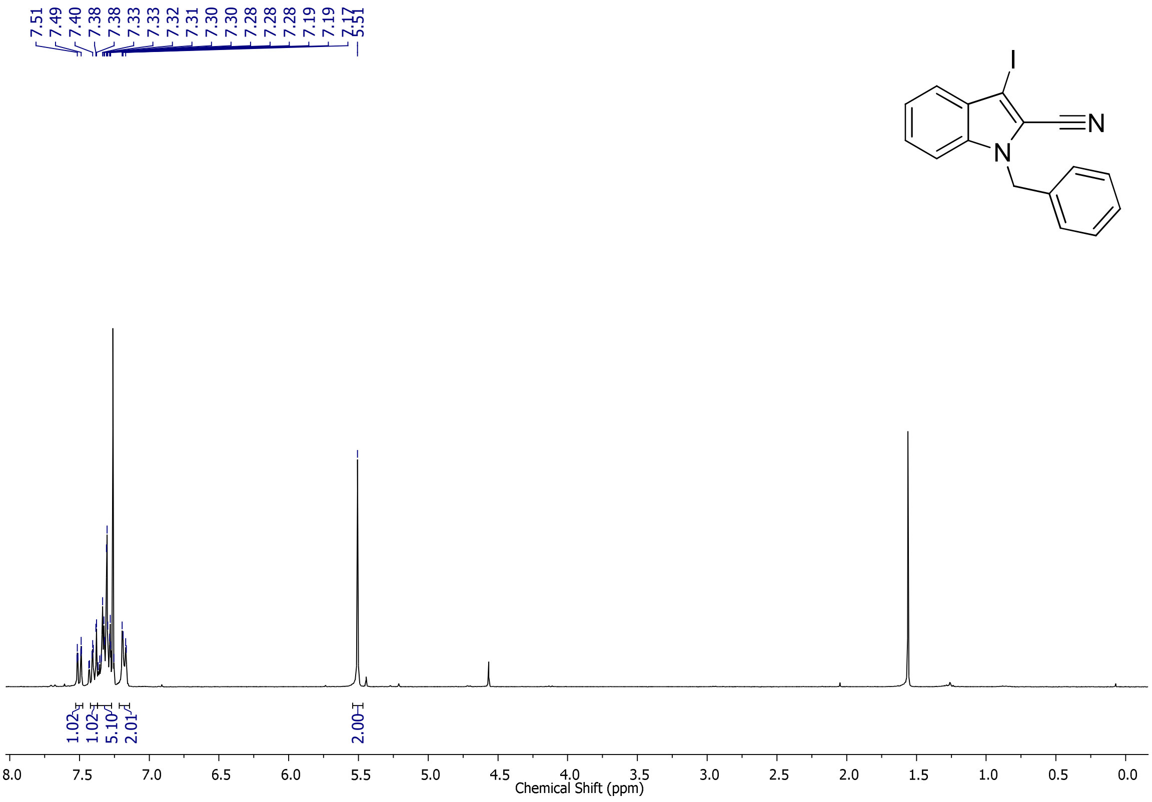

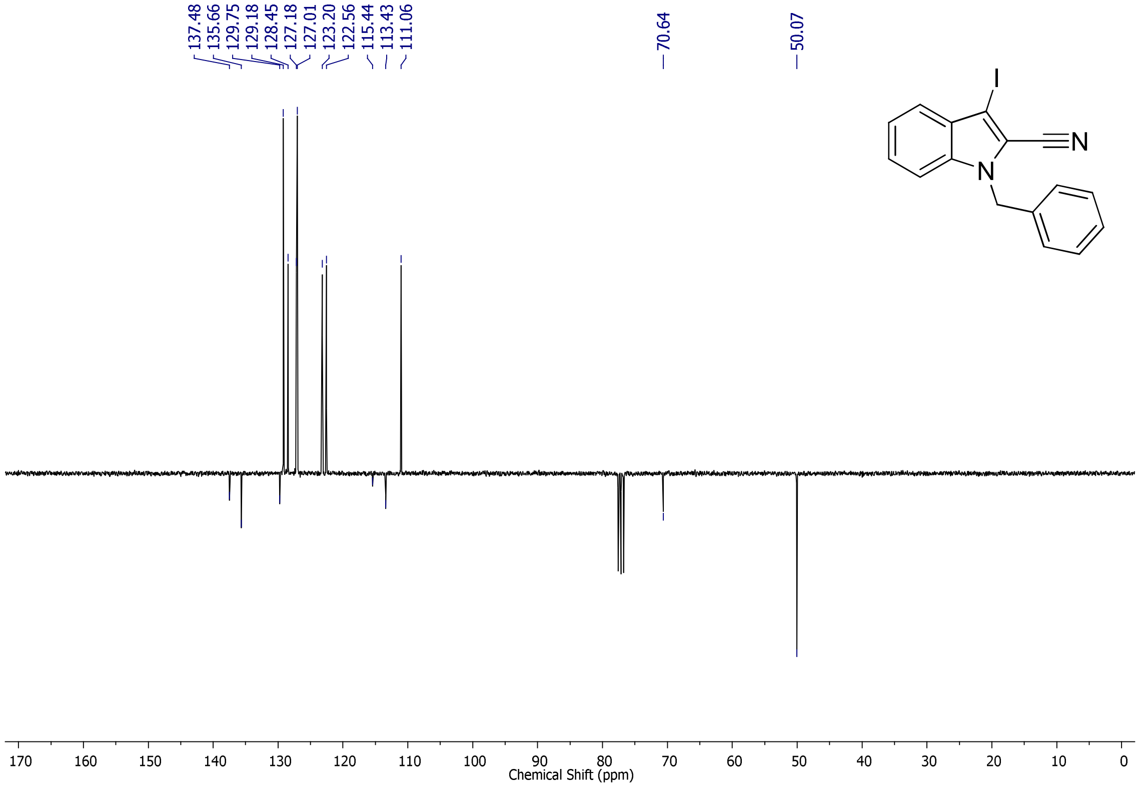

1-Benzyl-3-iodo-5-methoxy-1*H*-indole-2-carbonitrile (7b)

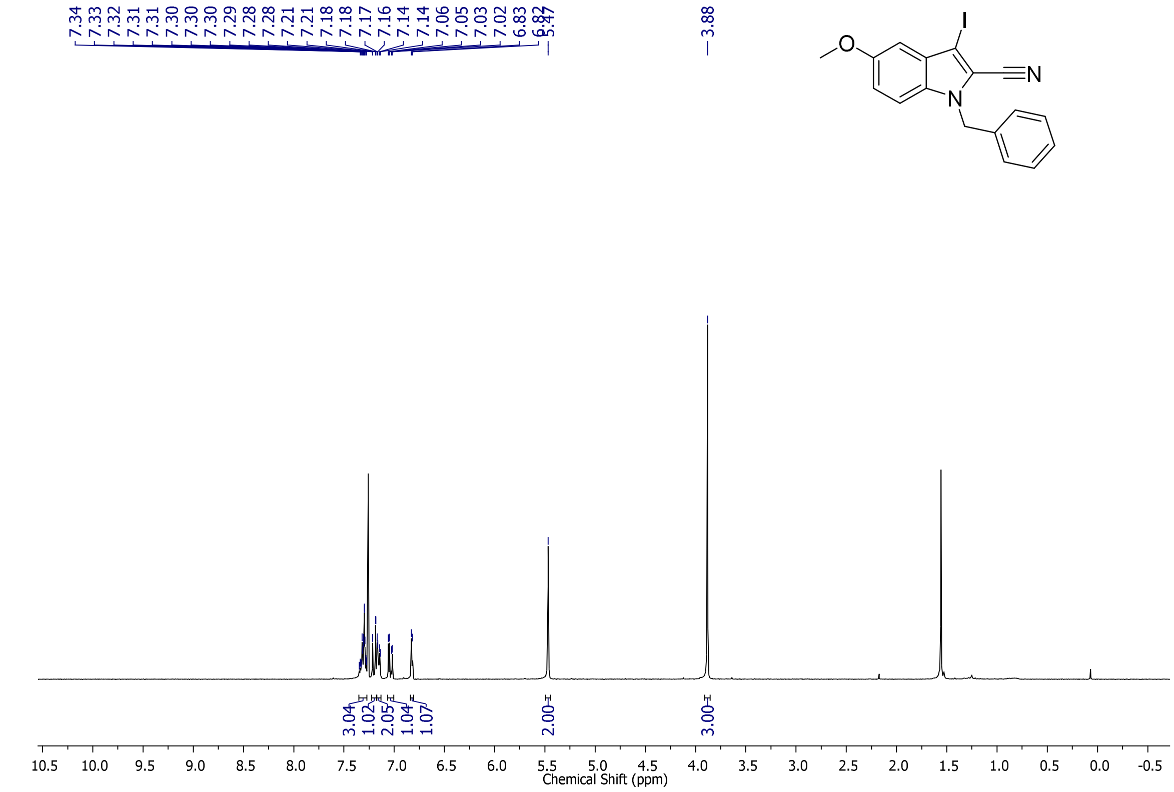

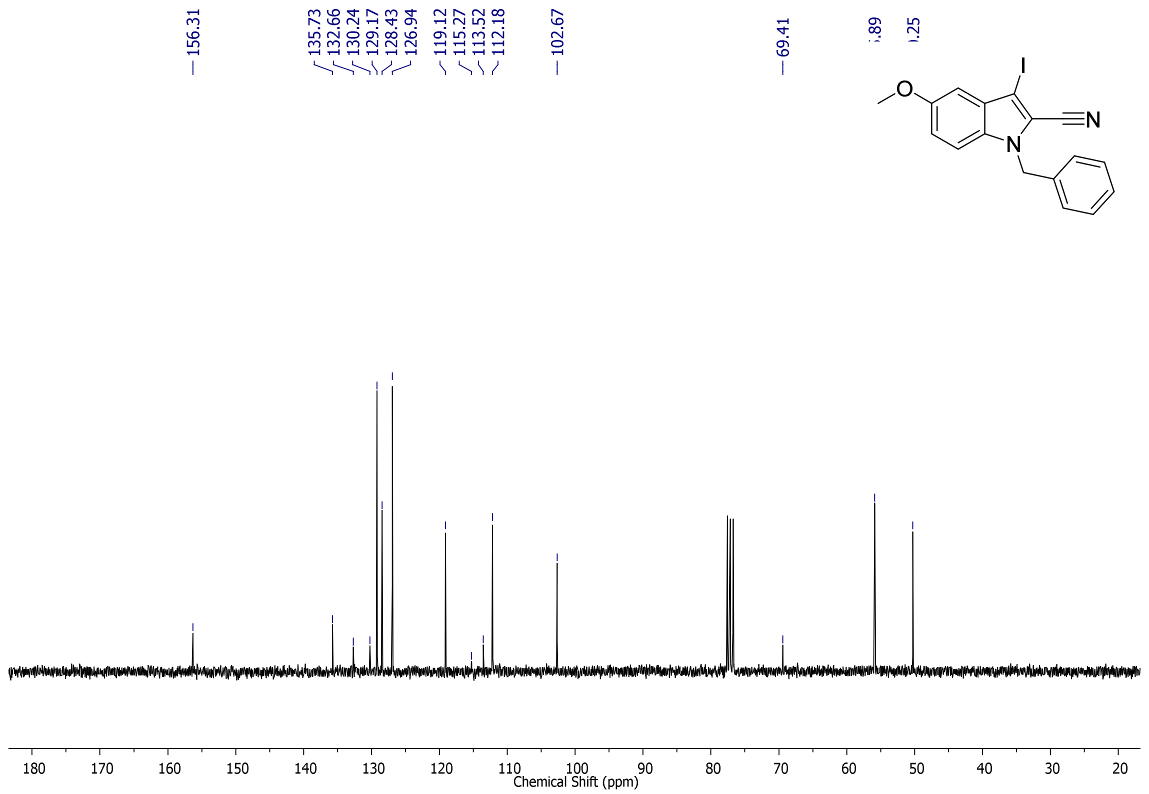

1-Benzyl-3-iodo-6-methoxy-1*H*-indole-2-carbonitrile (7c)

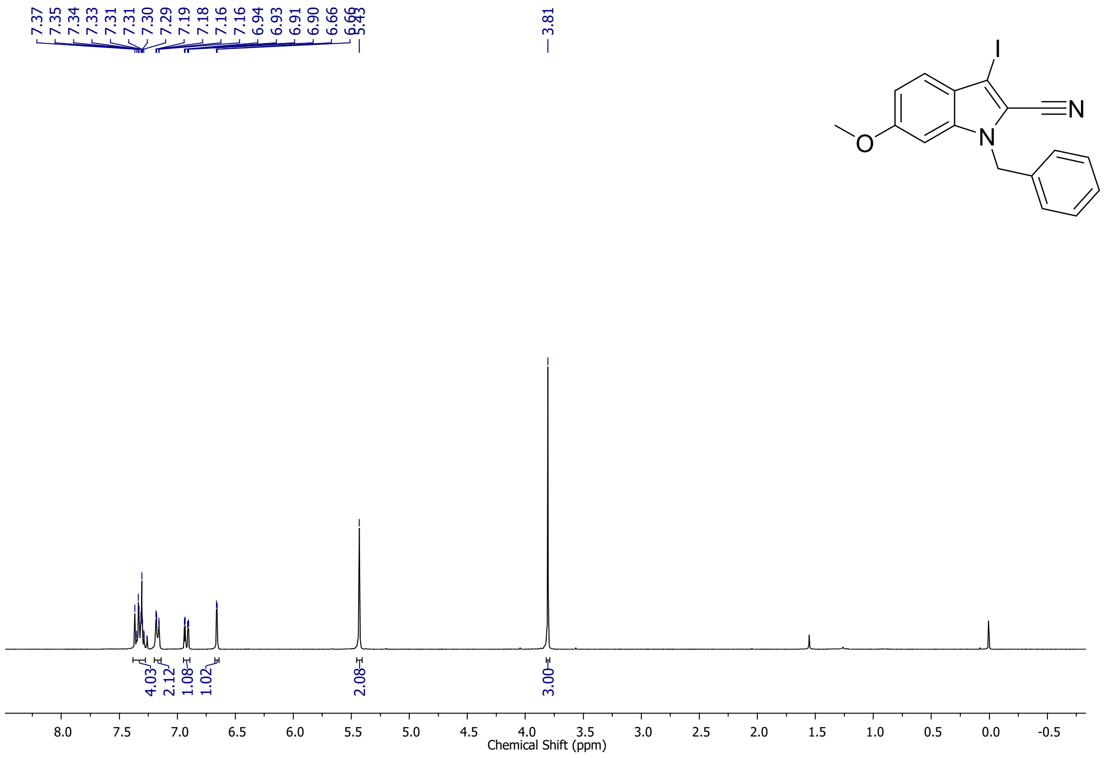

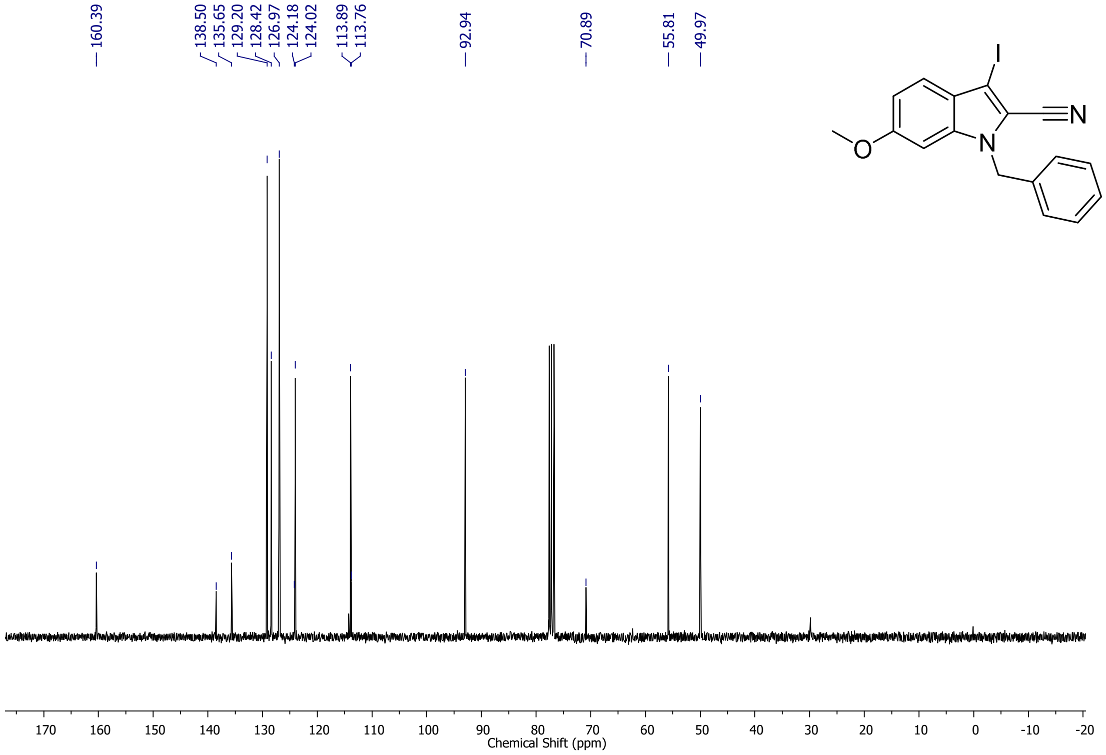

1-Benzyl-5-fluoro-3-iodo-1*H*-indole-2-carbonitrile (7d)

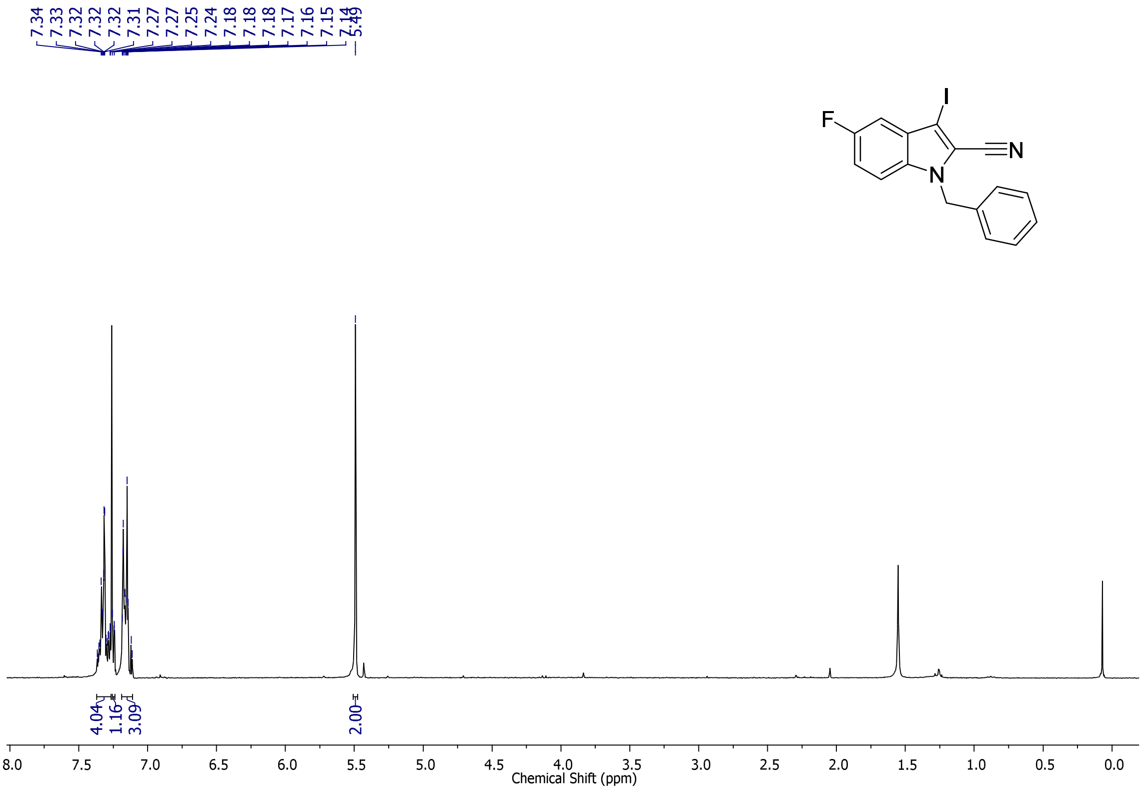

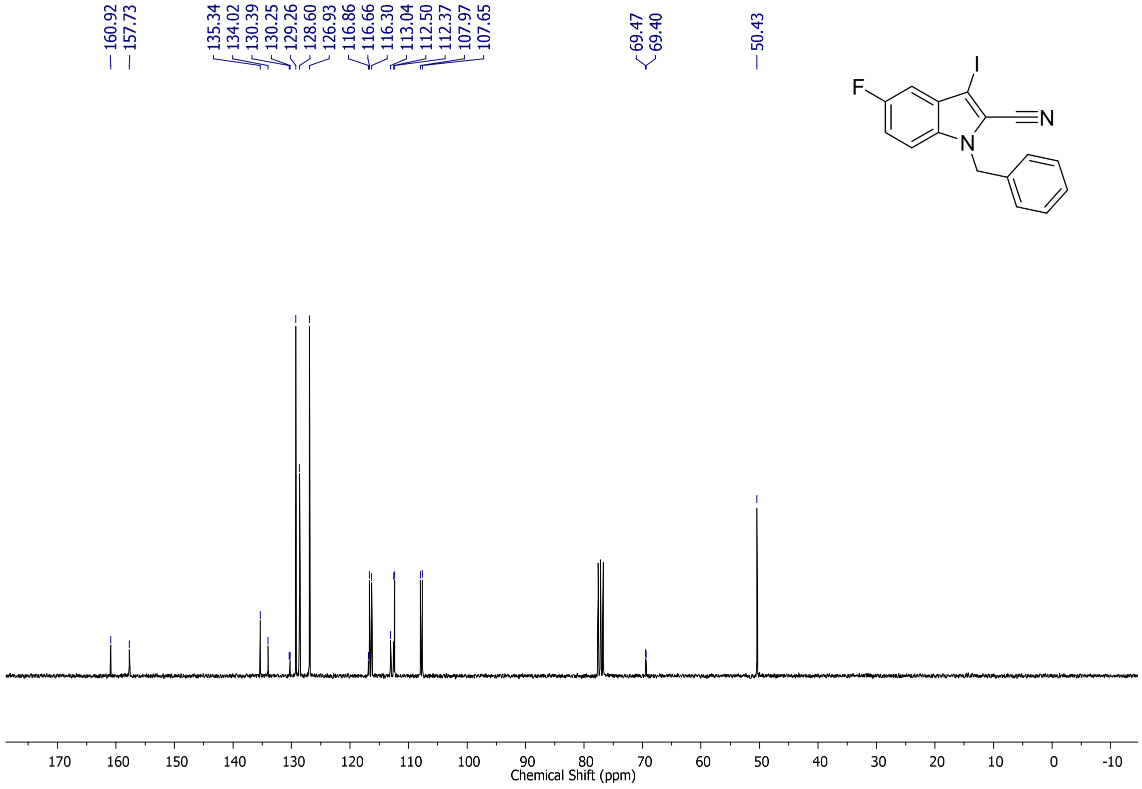

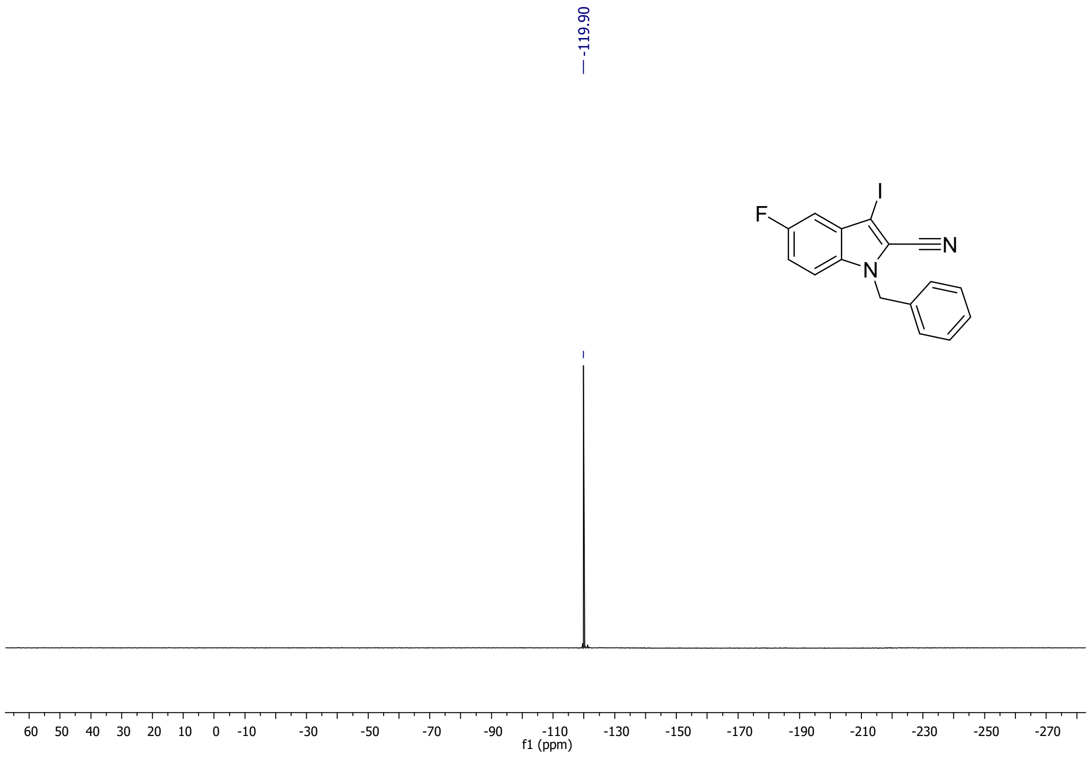
-119.90
F
I
N
N
60 50 40 30 20 10 0 -10 -30 -50 -70 -90 -110 -130 -150 -170 -190 -210 -230 -250 -270
f1 (ppm)

1-Benzyl-3-(phenylethynyl)-1*H*-indole-2-carbonitrile (8a)

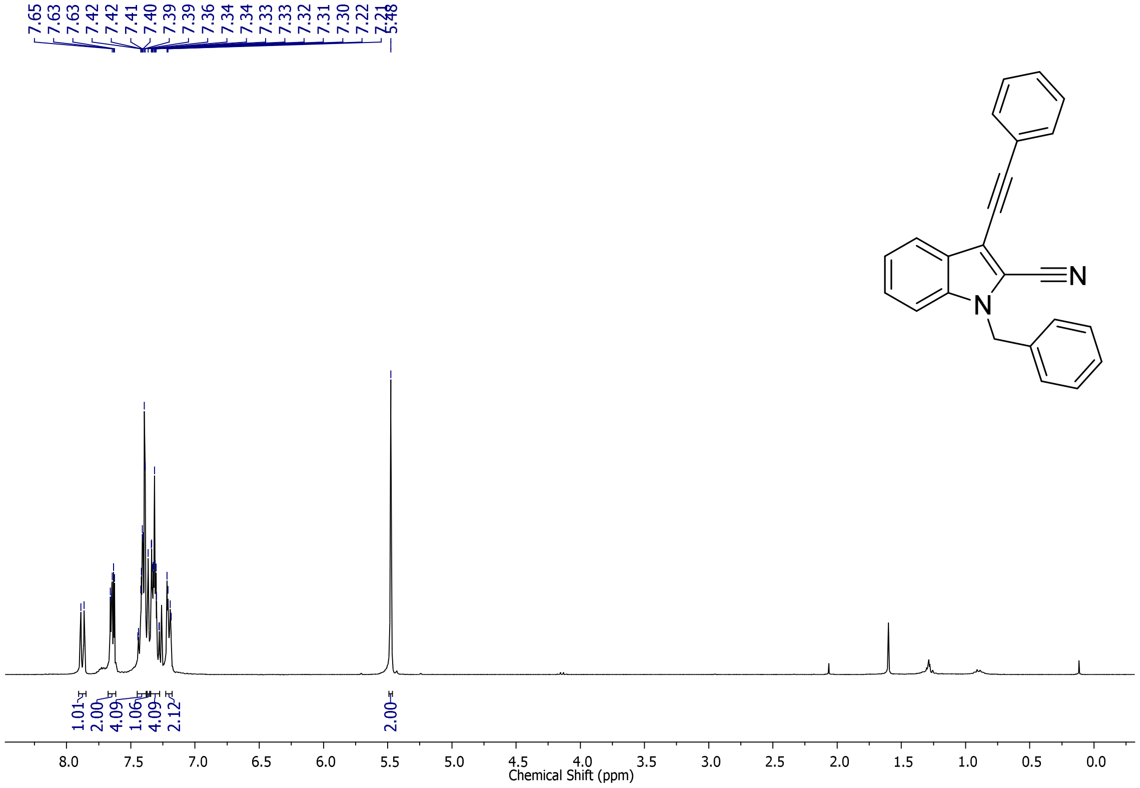

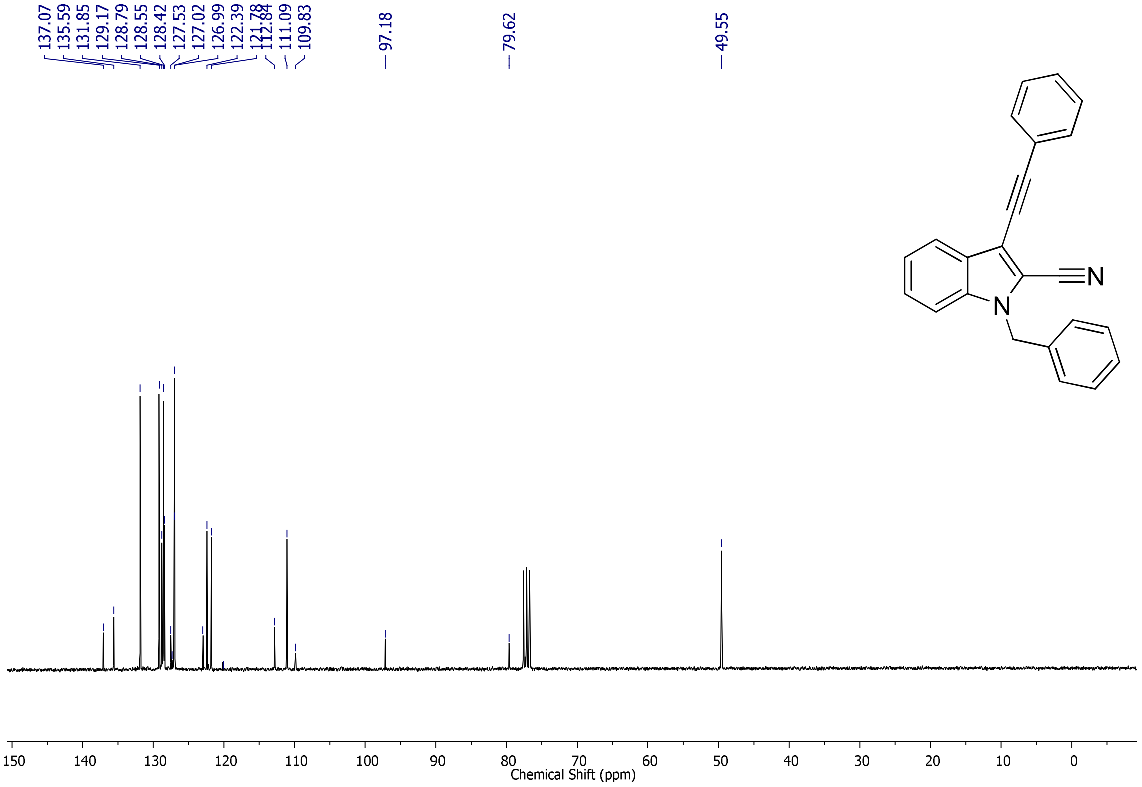

1-Benzyl-3-(*p*-tolylethynyl)-1*H*-indole-2-carbonitrile (8b)

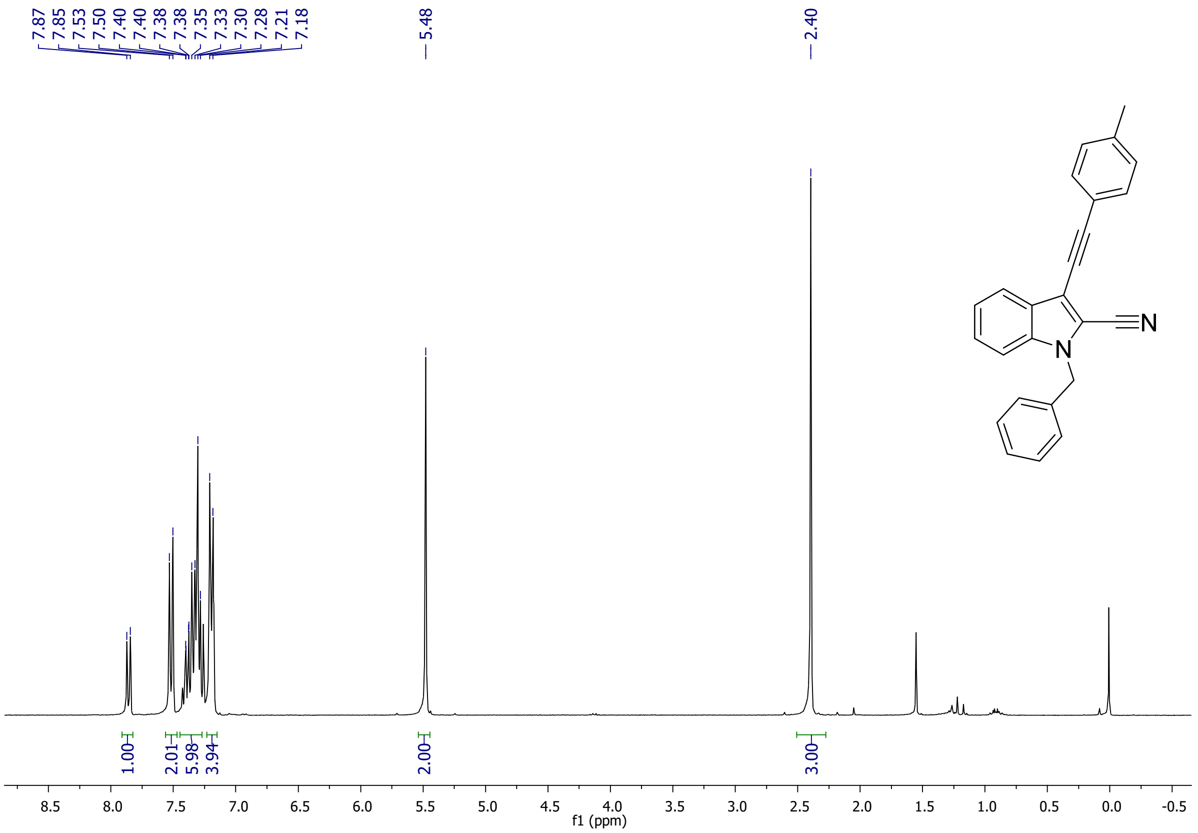

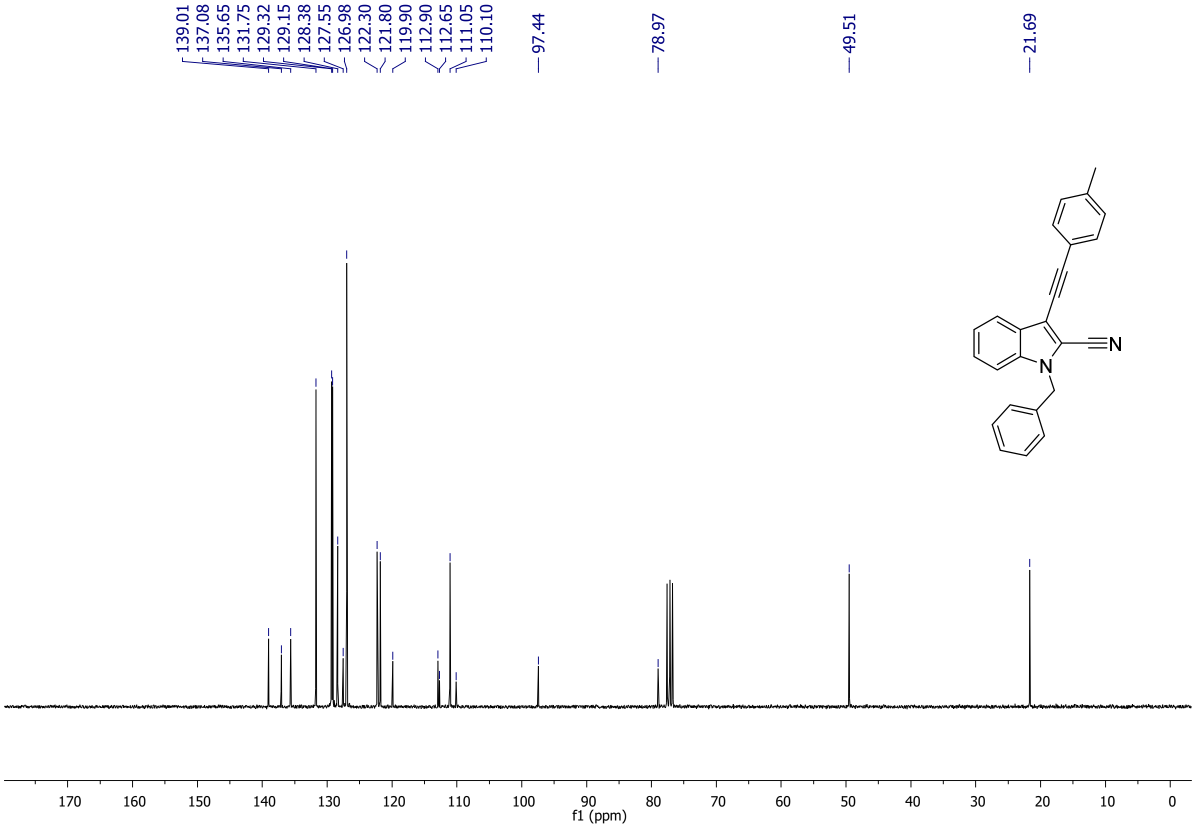

1-Benzyl-3-((4-fluorophenyl)ethynyl-1*H*-indole-2-carbonitrile (8c)

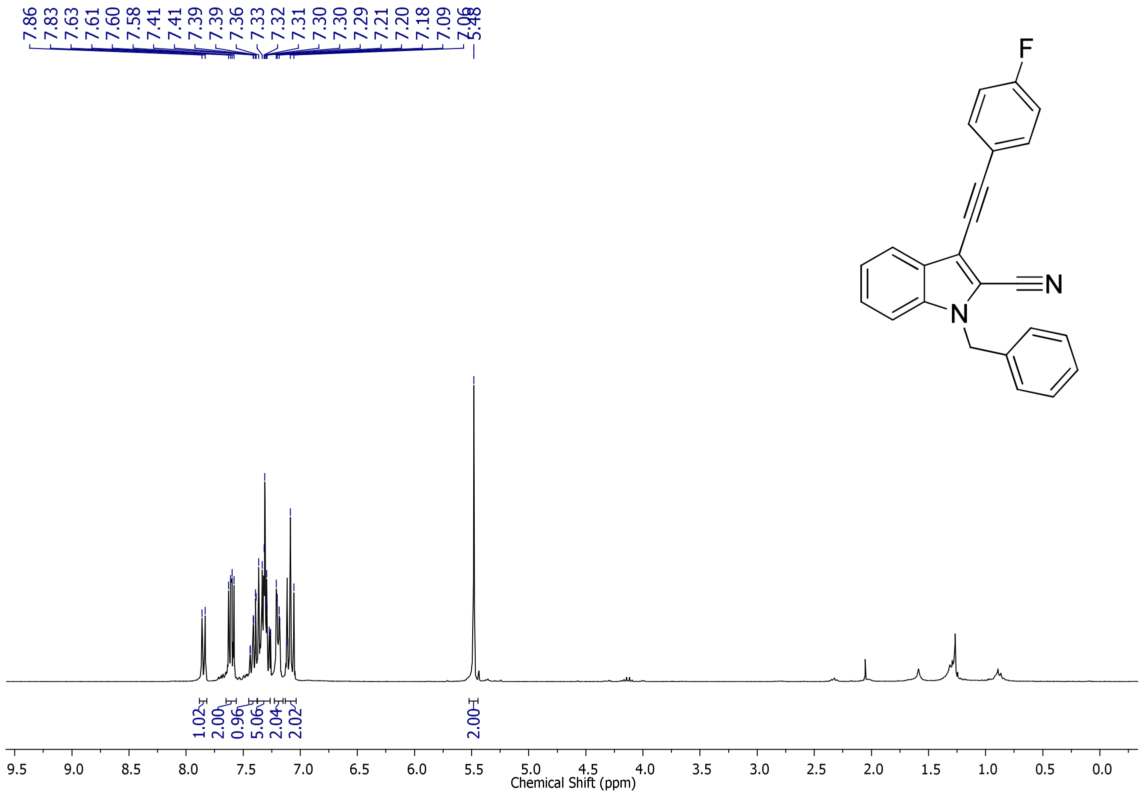

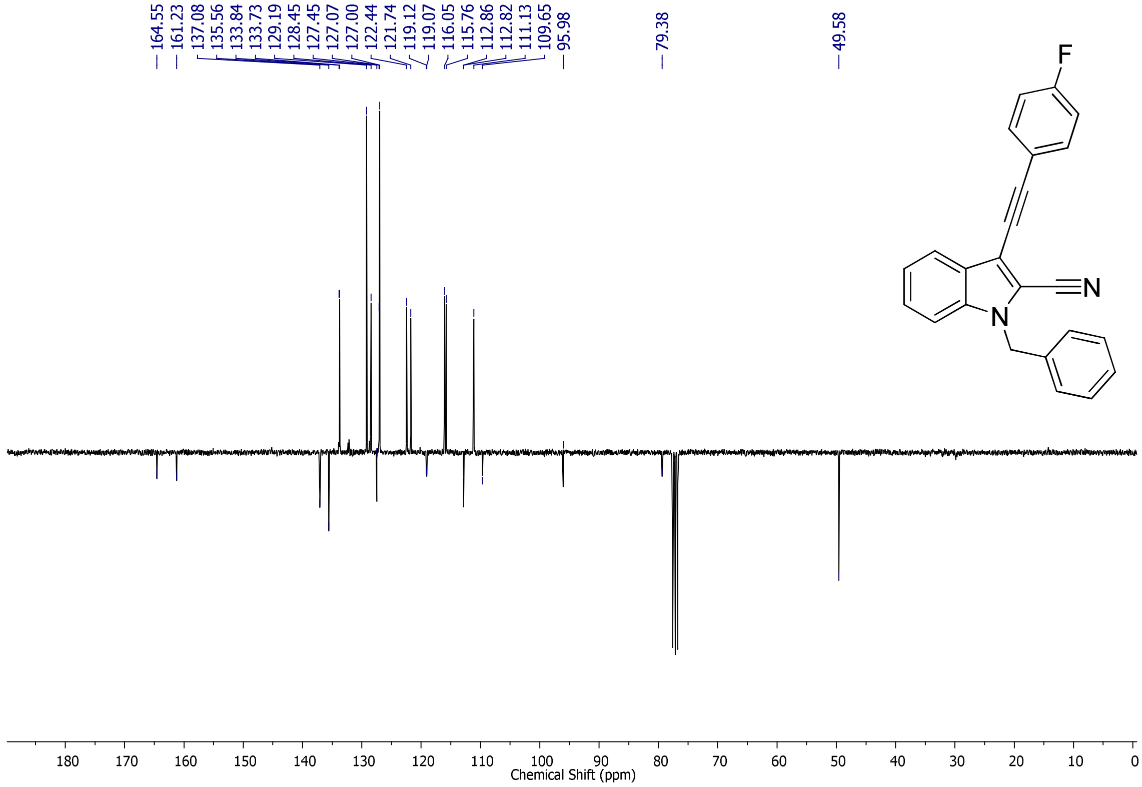

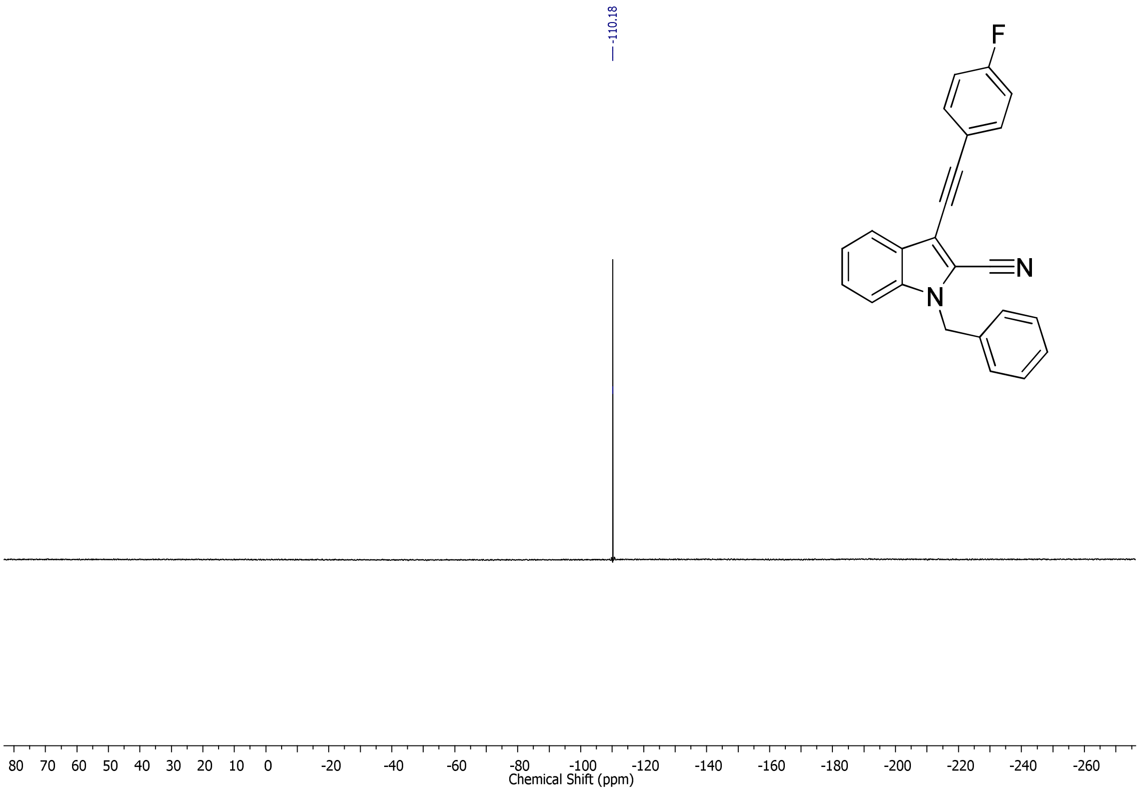

—-110.18
F
N
N
80 70 60 50 40 30 20 10 0 -20 -40 -60 -80 -100 -120 -140 -160 -180 -200 -220 -240 -260
Chemical Shift (ppm)

1-Benzyl-3-((4-methoxyphenyl)ethynyl)-1*H*-indole-2-carbonitrile (8d)

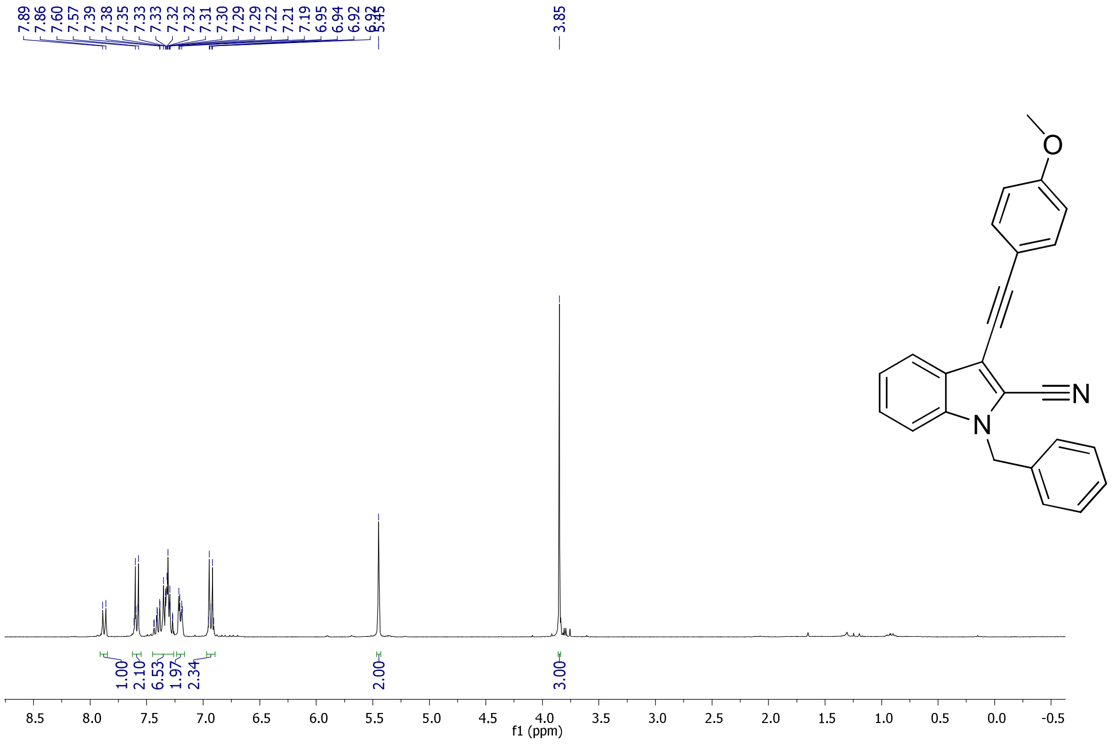

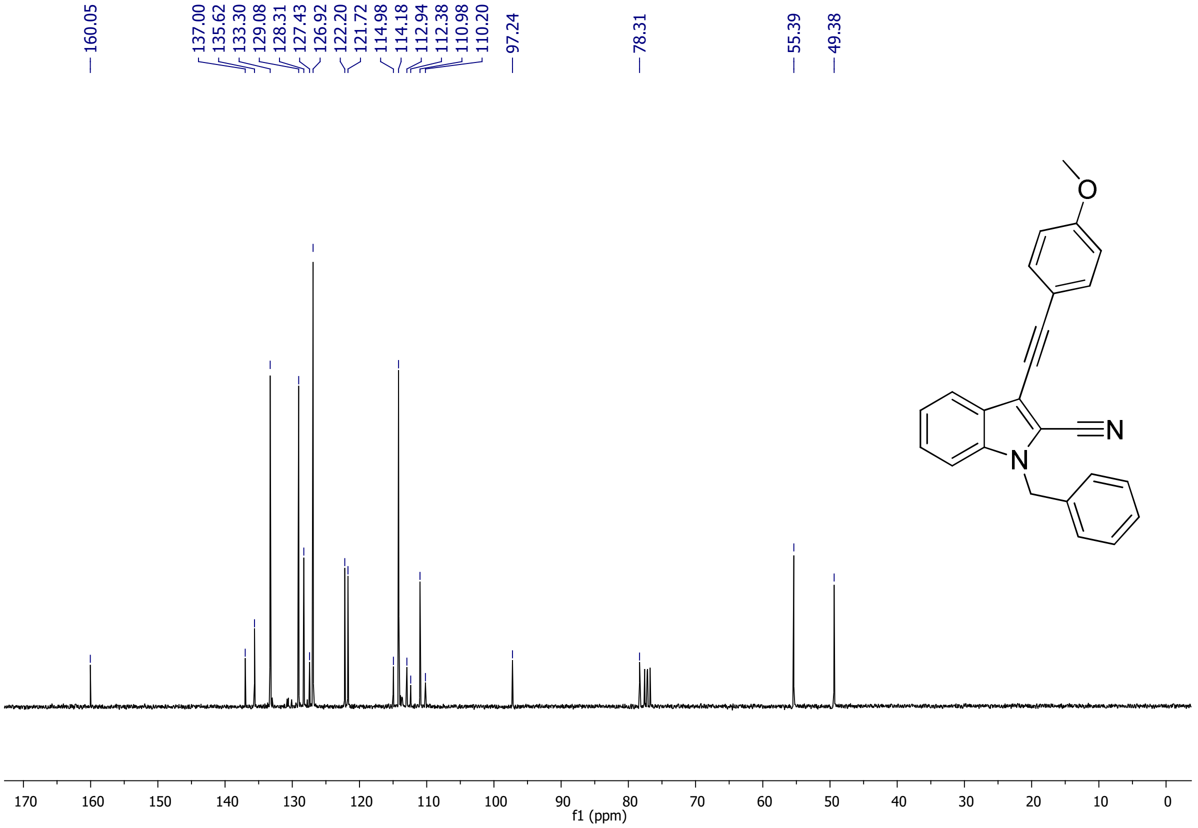

1-Benzyl-3-((4-ethylphenyl)ethynyl)-1*H*-Indole-2-Carbonitrile (8e)

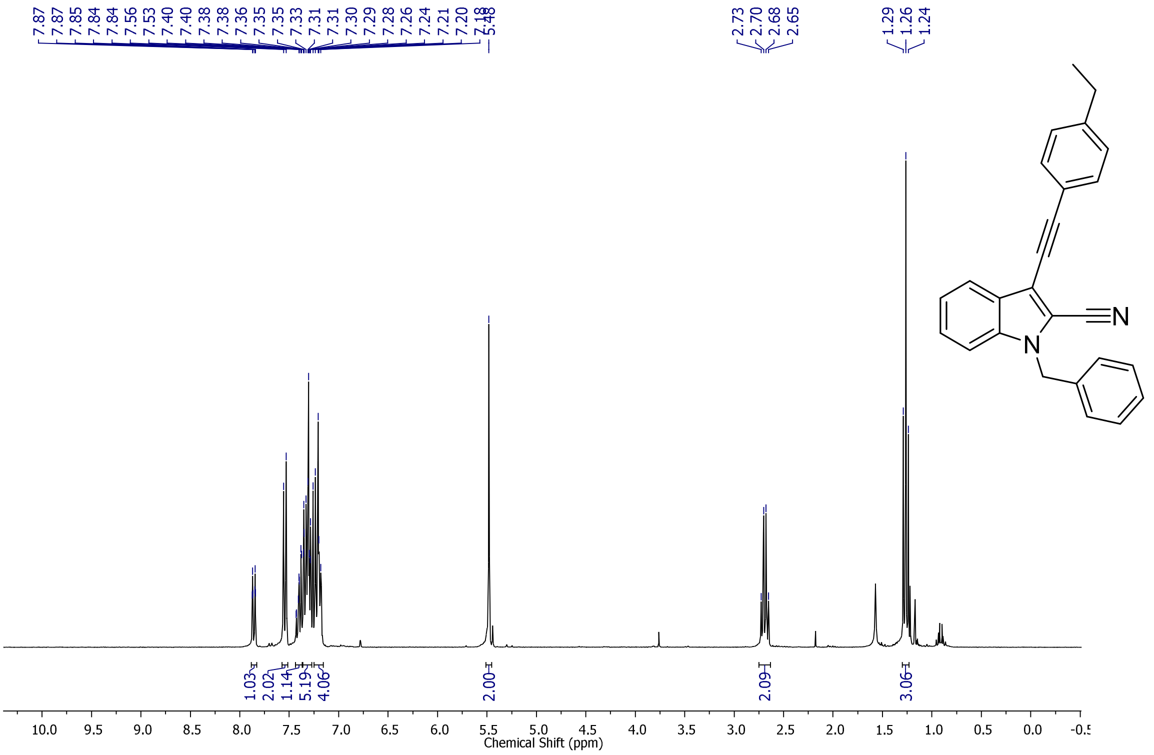

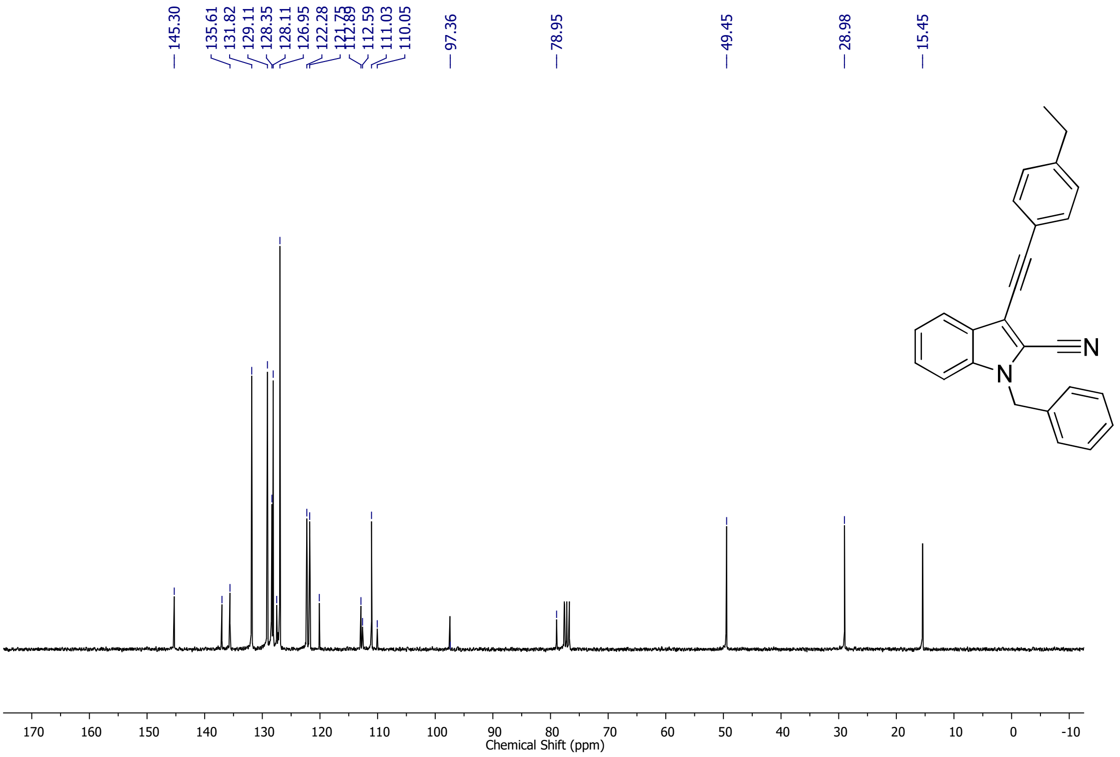

1-Benzyl-3-(pyridin-2-yl-ethynyl)-1*H*-Indole-2-Carbonitrile (8f)

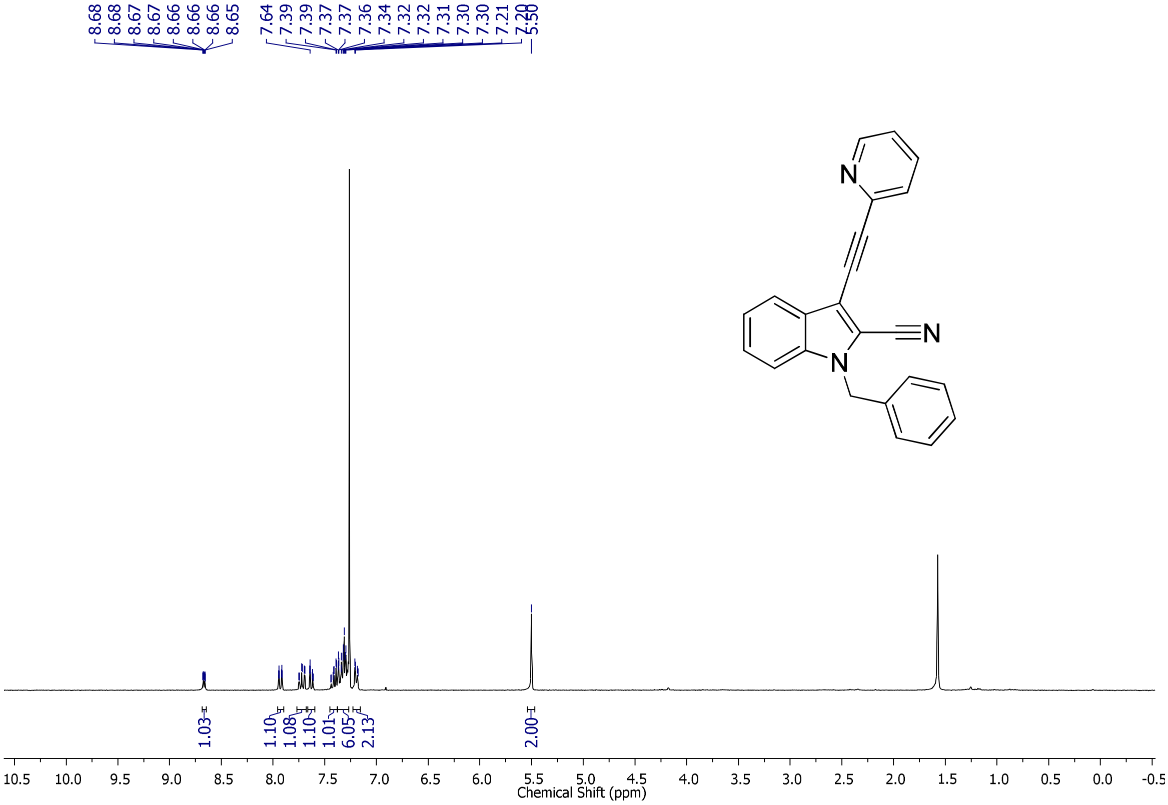

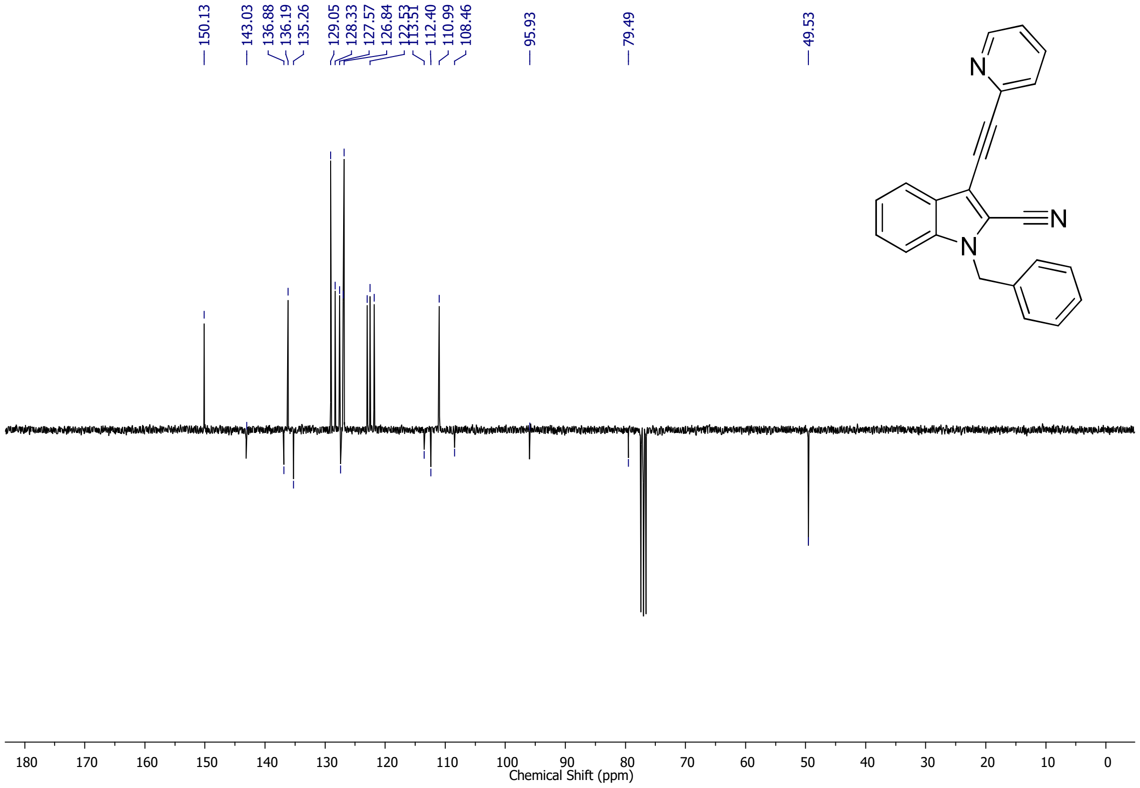

1-Benzyl-3-((2-fluorophenyl)ethynyl)-1*H*-indole-2-carbonitrile (8g)

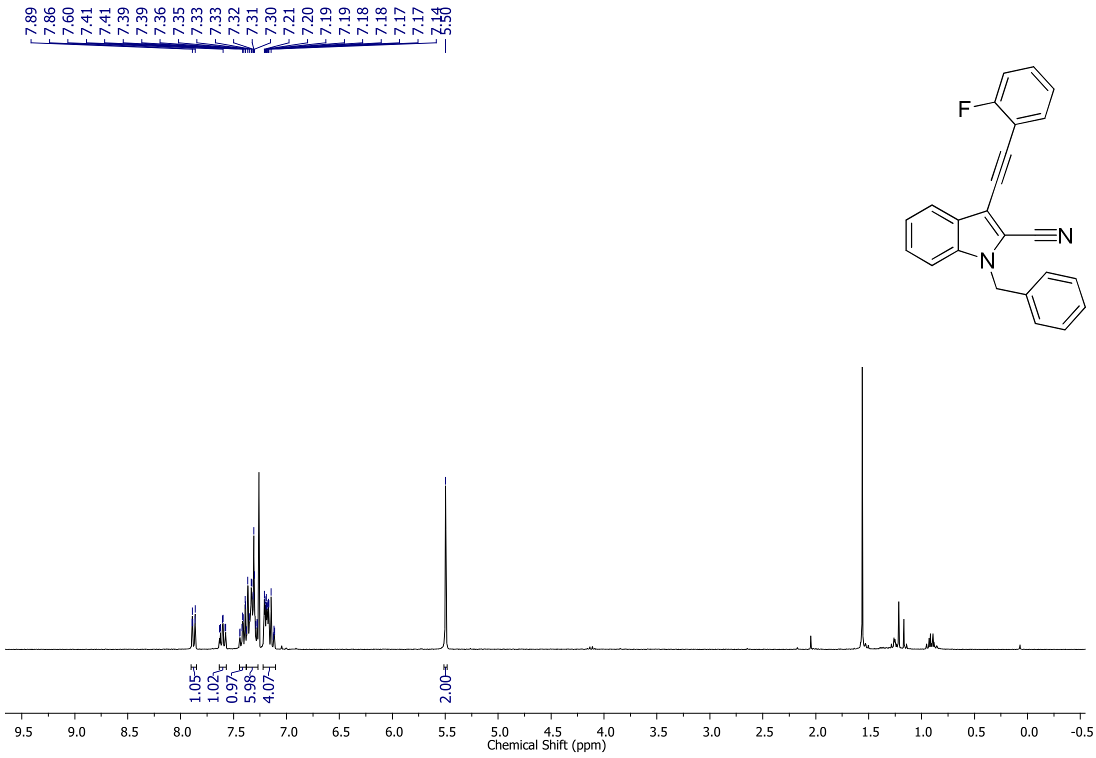

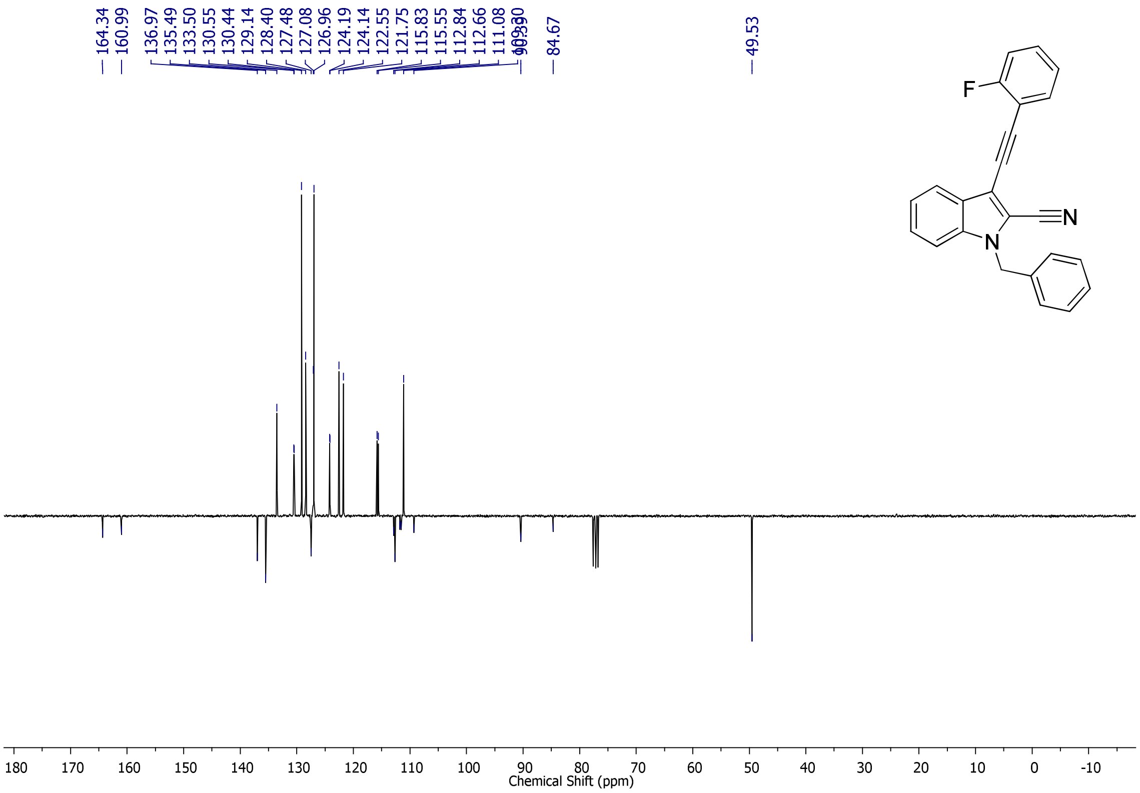

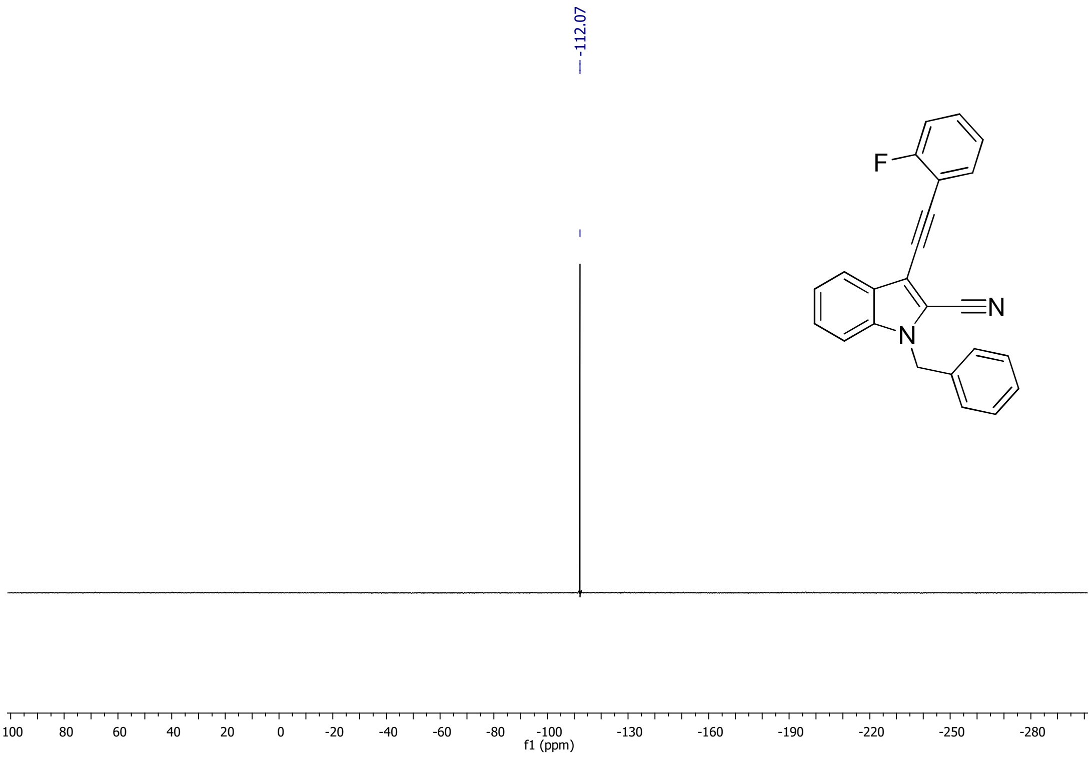
-112.07
F
N
N
100
80
60
40
20
0
-20
-40
-60
-80
-100
-130
-160
-190
-220
-250
-280
f1 (ppm)

1-Benzyl-5-methoxy-3-((4-methoxyphenyl)ethynyl)-1*H*-indole-2-carbonitrile (8h)

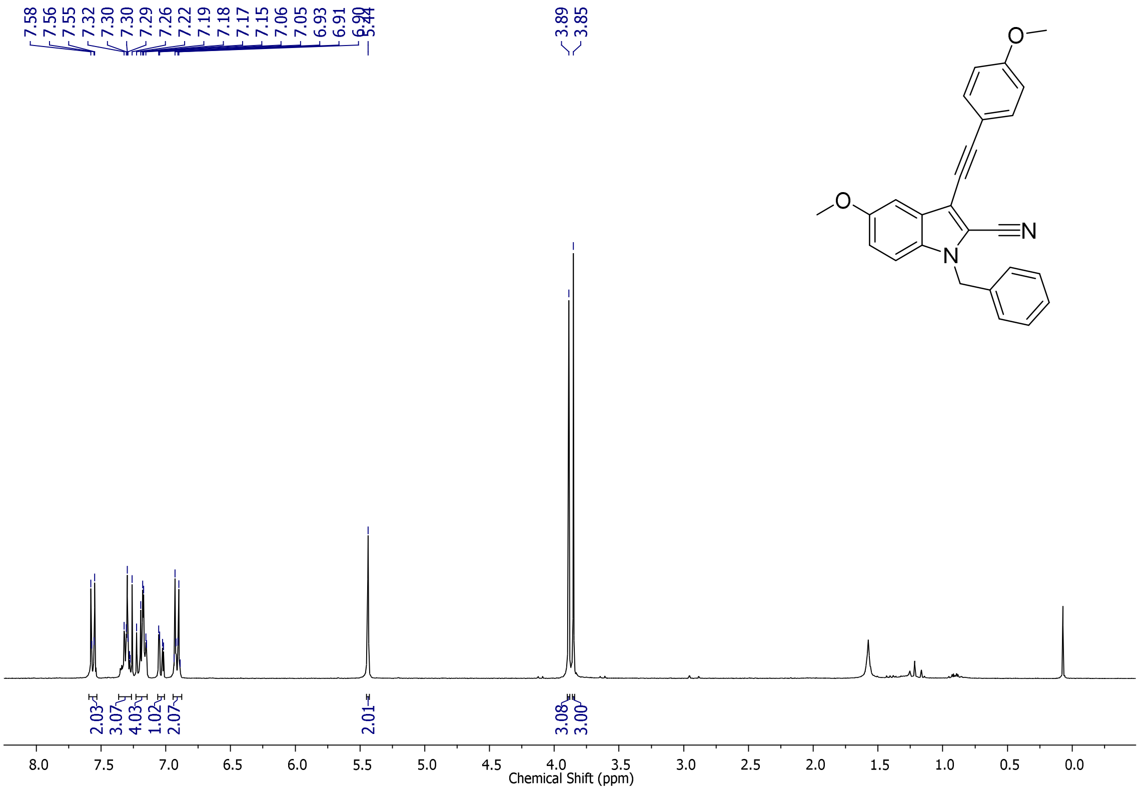

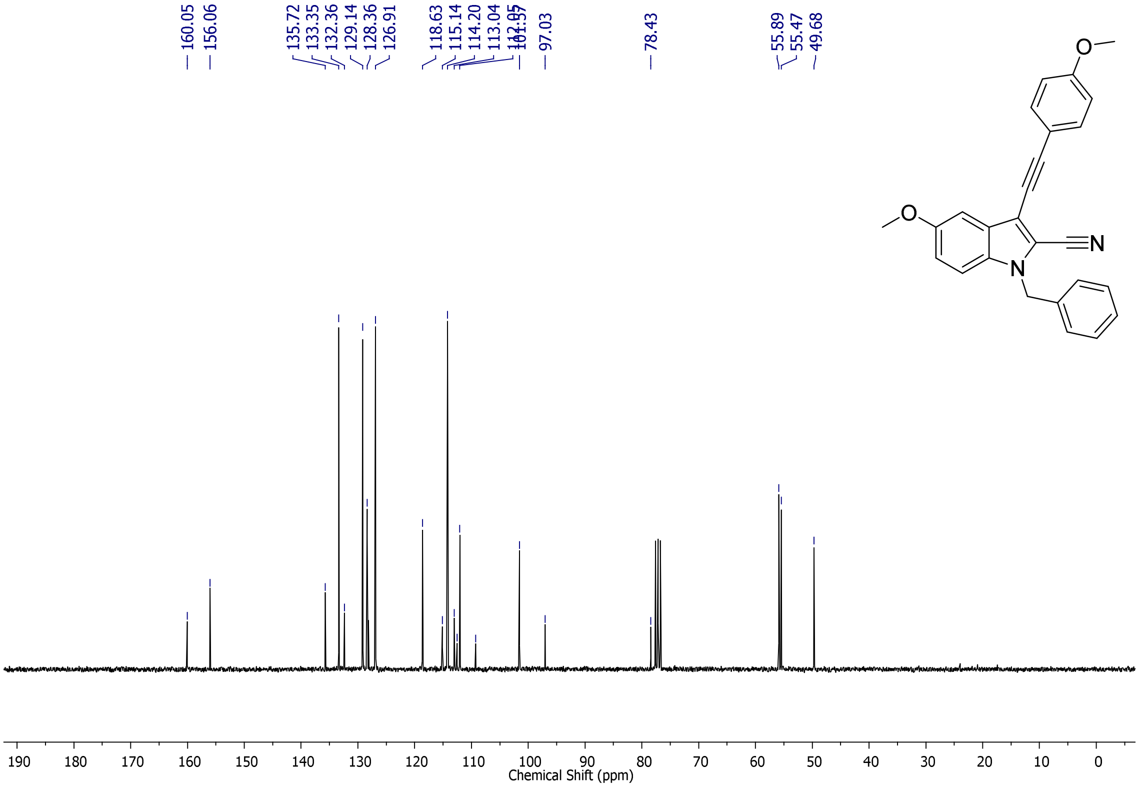

1-Benzyl-6-methoxy-3-((2-methoxyphenyl)ethynyl)-1*H*-indole-2-carbonitrile (8i)

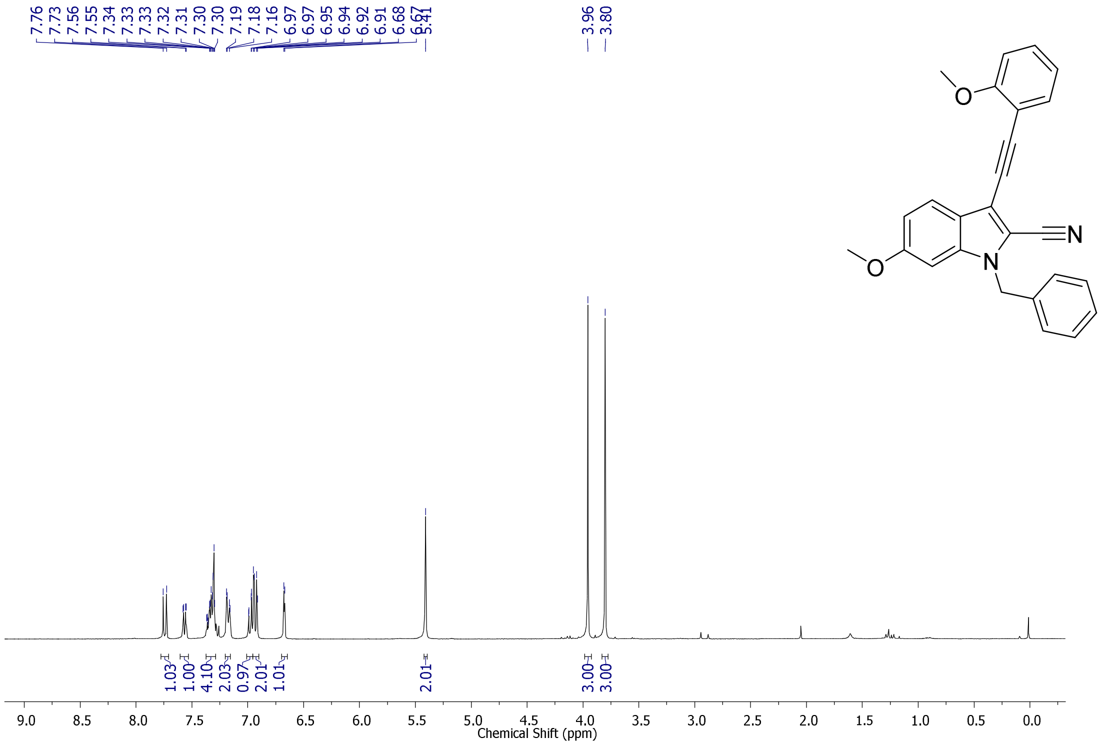

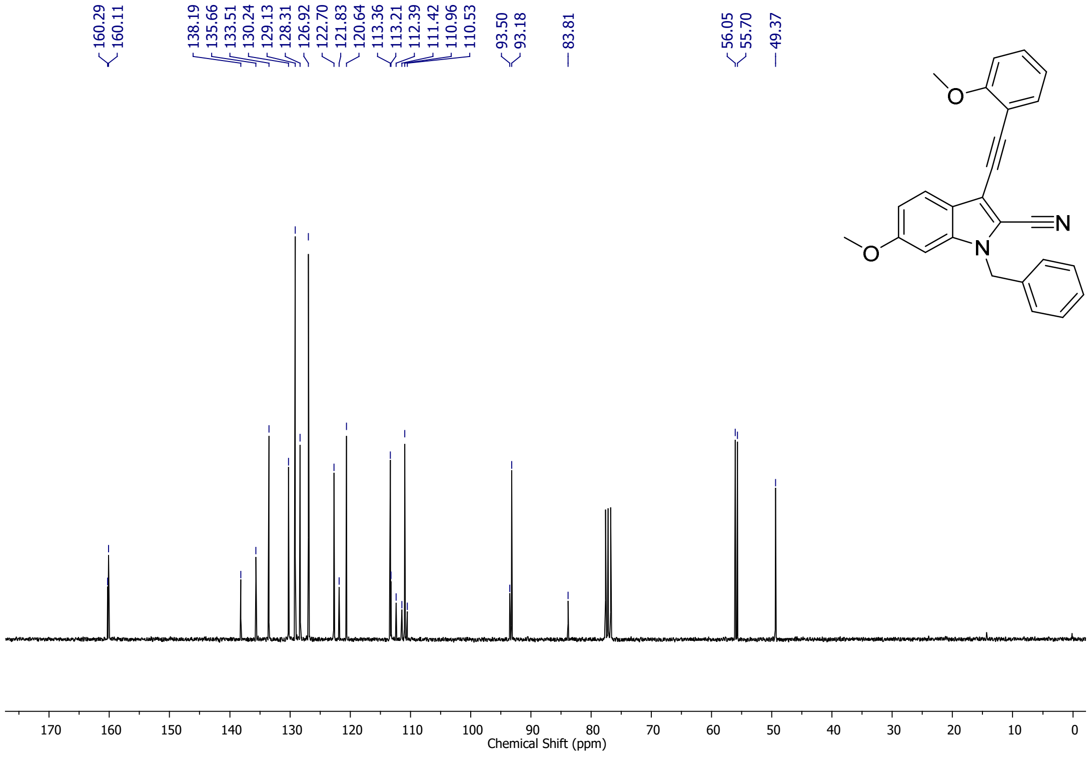

1-Benzyl-5-fluoro-3-(*m*-tolylethynyl)-1*H*-indole-2-carbonitrile (8j)

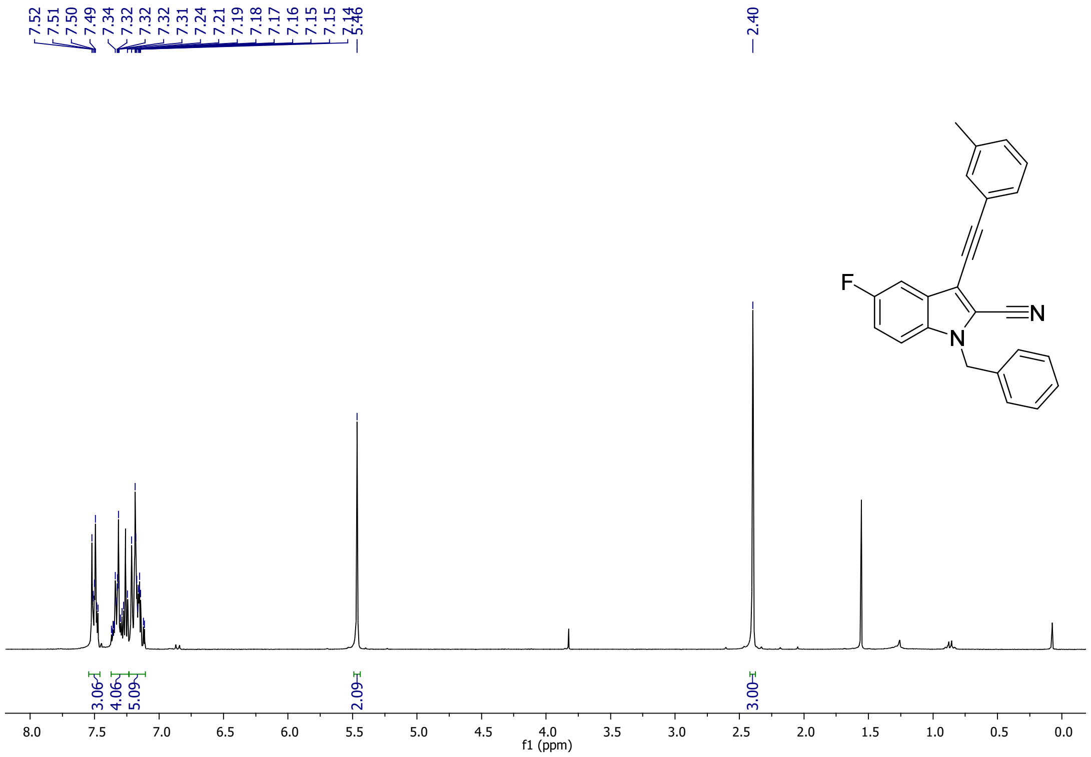

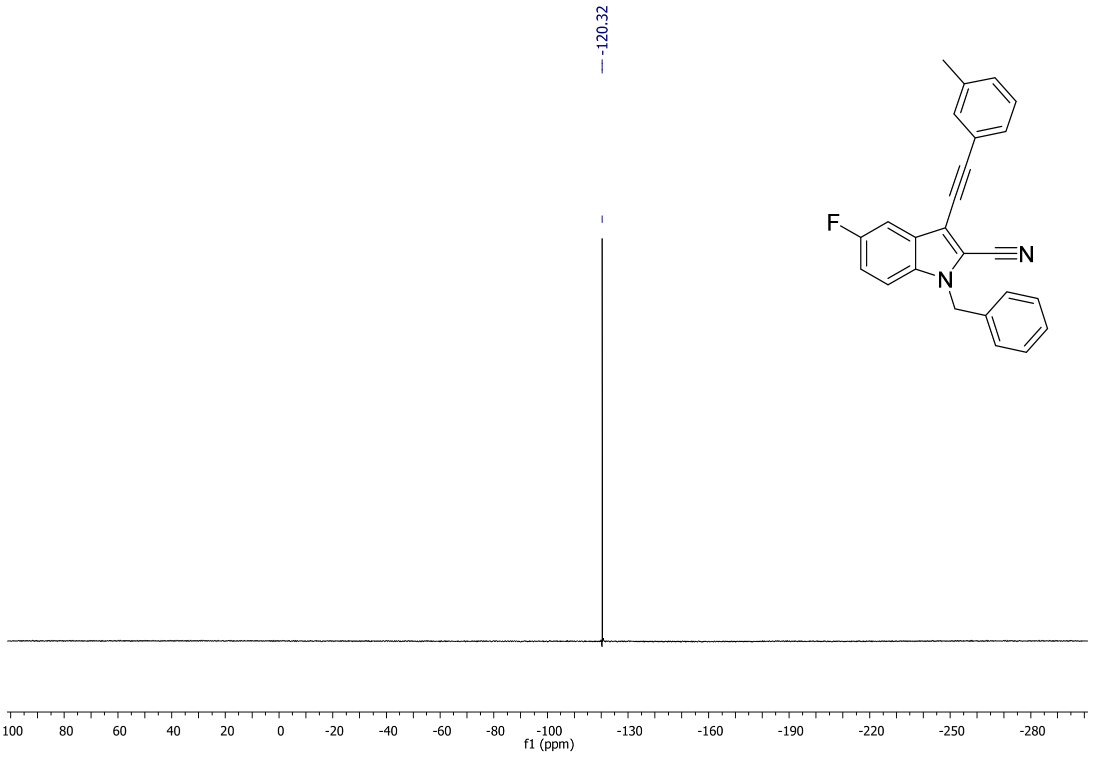

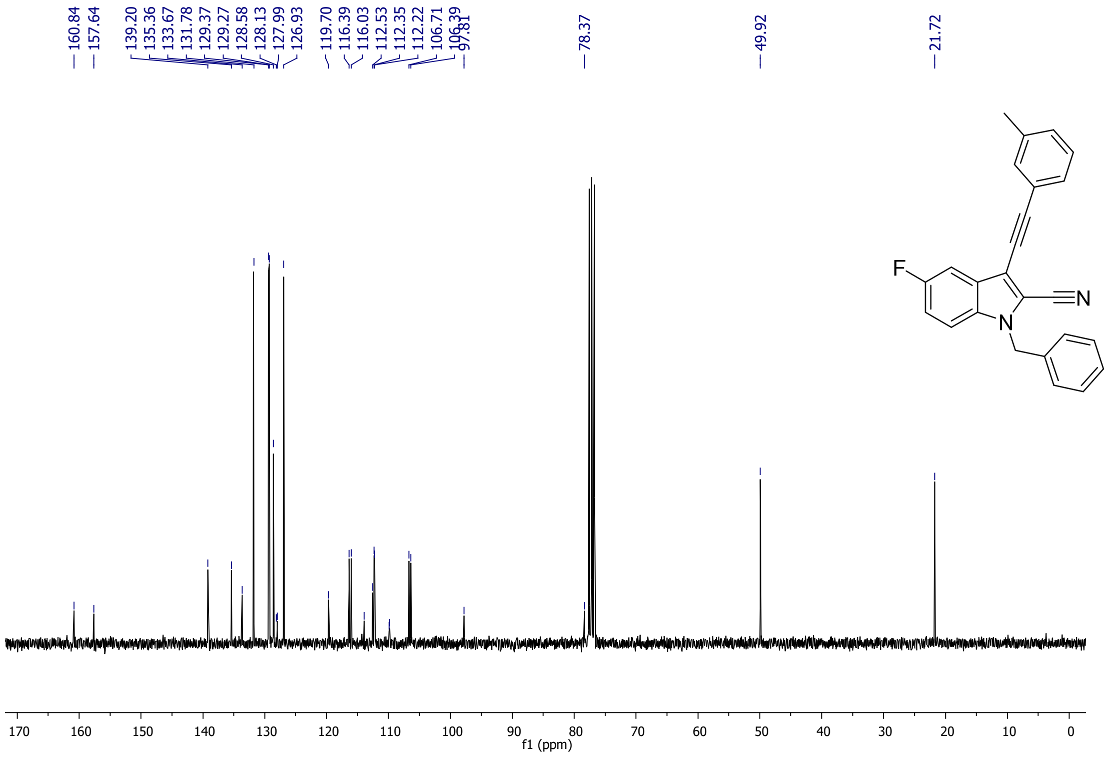
160.84
157.64
139.20
135.36
133.67
131.78
129.37
129.27
128.58
128.13
127.99
126.93
119.70
116.39
116.03
112.53
112.35
112.22
106.71
106.39
97.81
78.37
49.92
21.72
170
160
150
140
130
120
110
100
90
80
70
60
50
40
30
20
10
0
f1 (ppm)
F
N
N

1-Benzyl-3-(4-methoxyphenyl)-1*H*-indole-2-carbonitrile (9a)

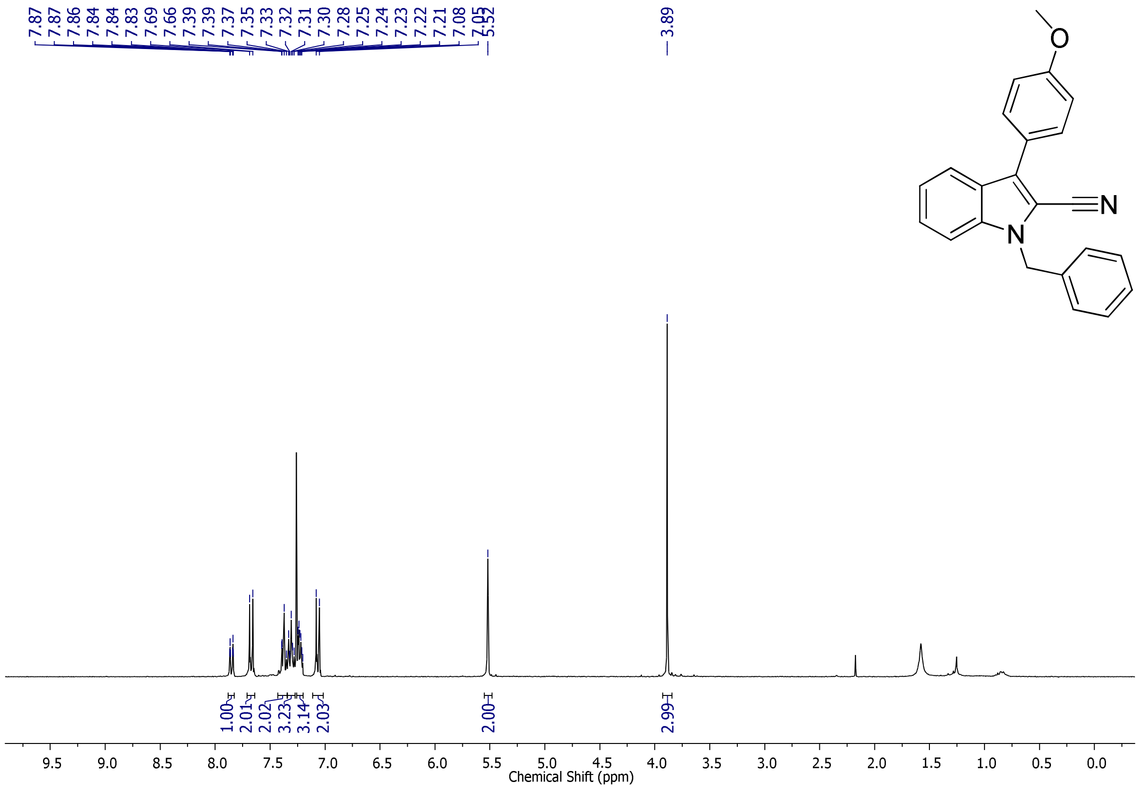

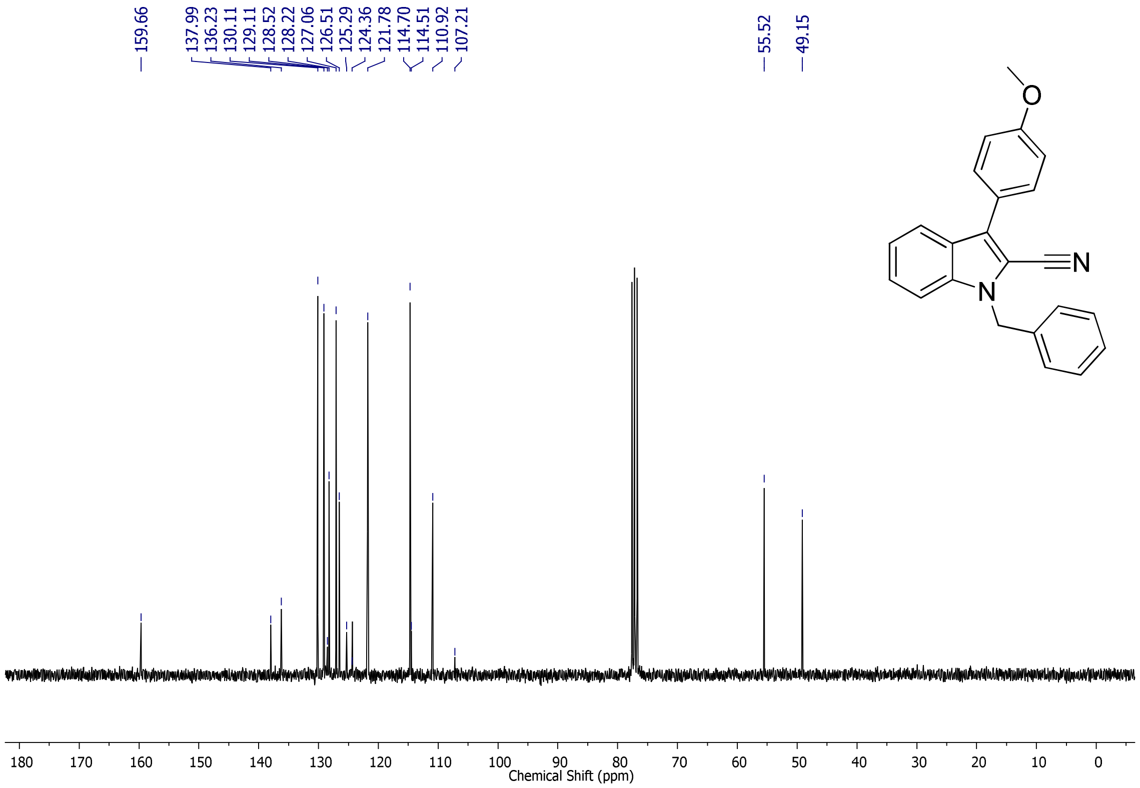

1-Benzyl-3-(4-fluorophenyl)-1*H*-indole-2-carbonitrile (9b)

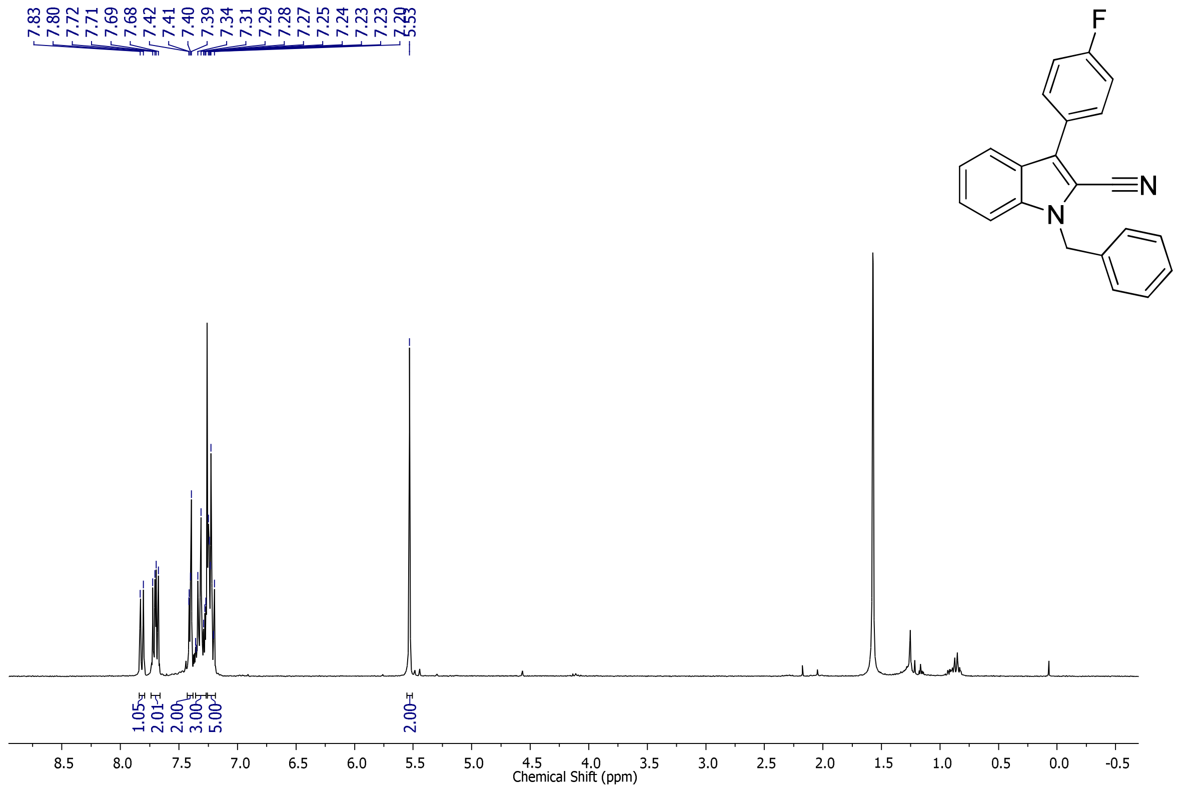

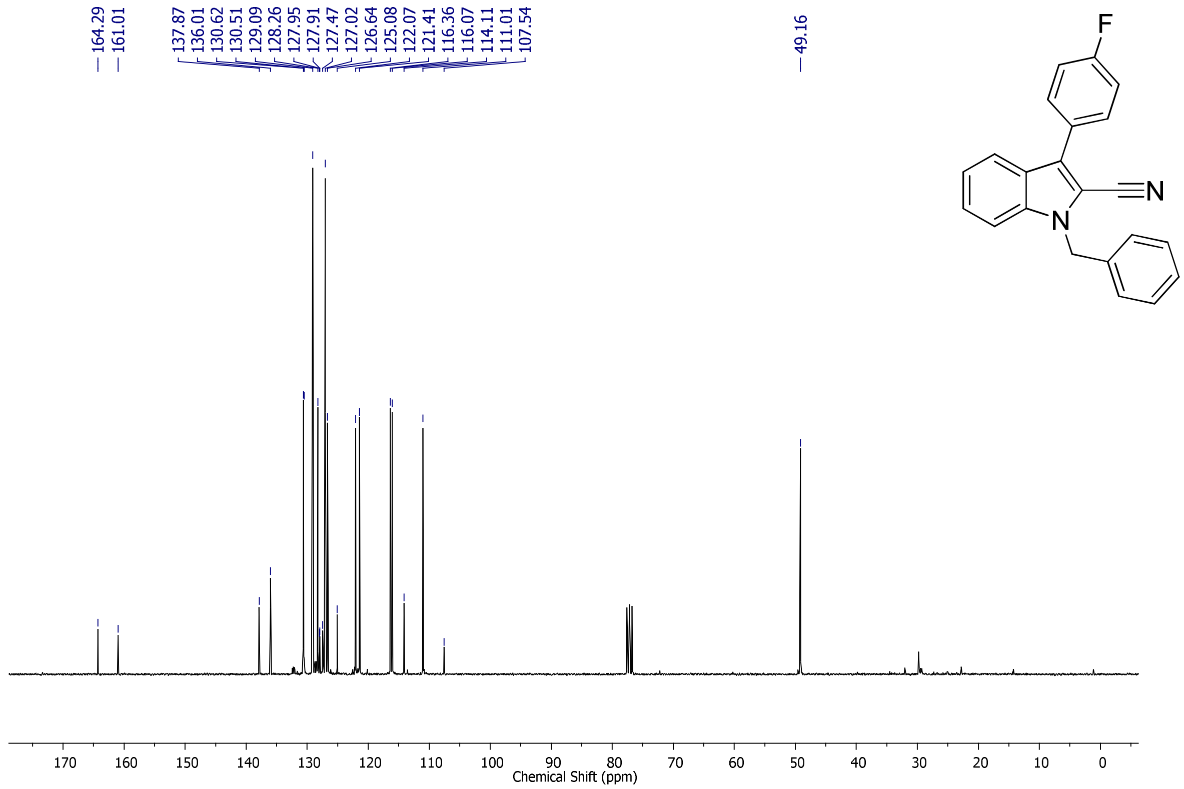

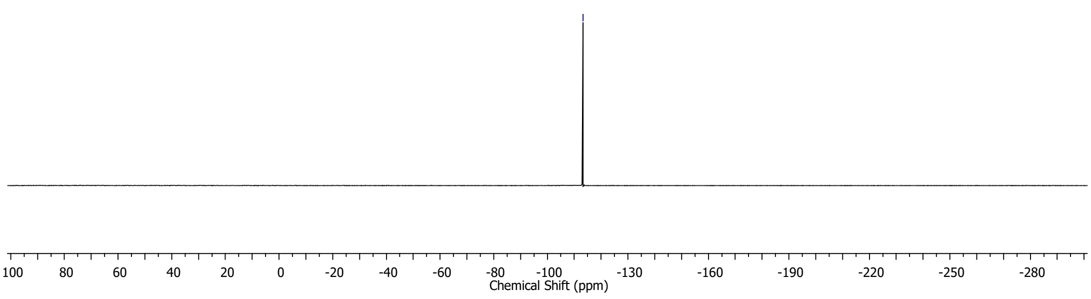
-113.24
100 80 60 40 20 0 -20 -40 -60 -80 -100 -130 -160 -190 -220 -250 -280
Chemical Shift (ppm)

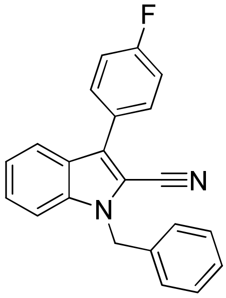
F
N
N

1-Benzyl-3-(4-*tert*-butylphenyl)-4-indole-2-carbonitrile (9c)

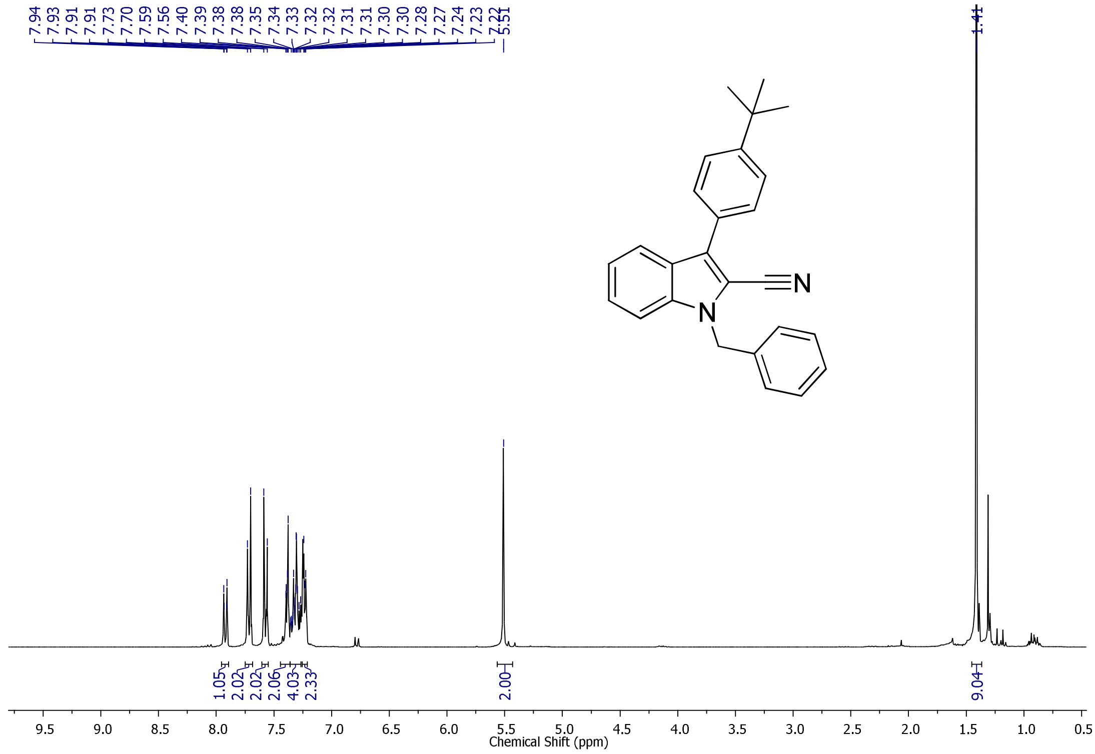

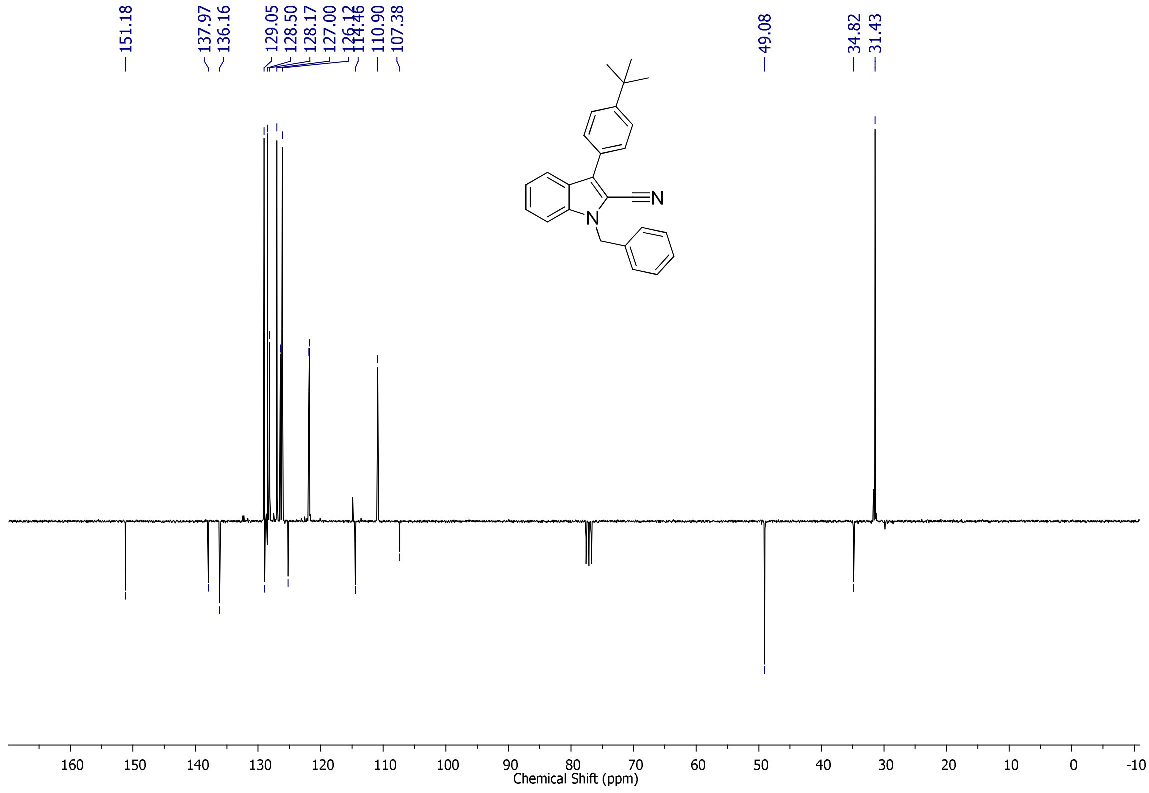

1-Benzyl-3-(4-(methylthio)phenyl)-1*H*-indole-2-carbonitrile (9d)

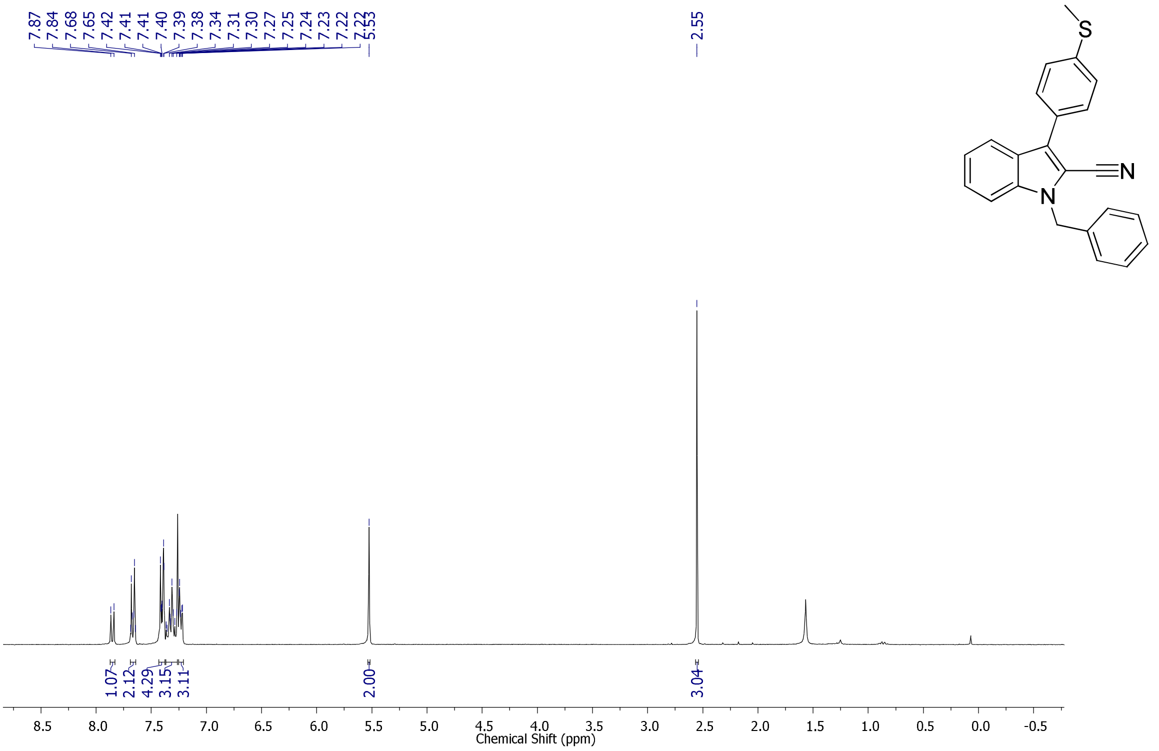

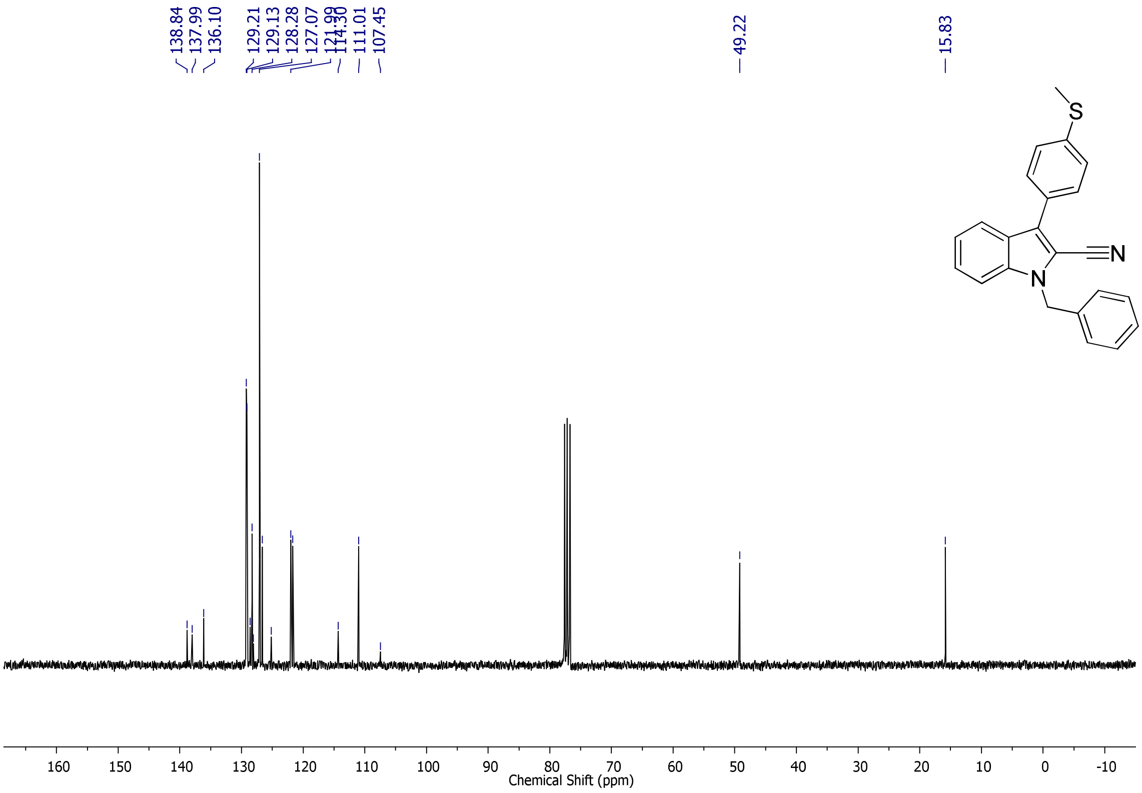

1-Benzyl-3-(4-(trifluoromethyl)phenyl)-1*H*-indole-2-carbonitrile (9e)

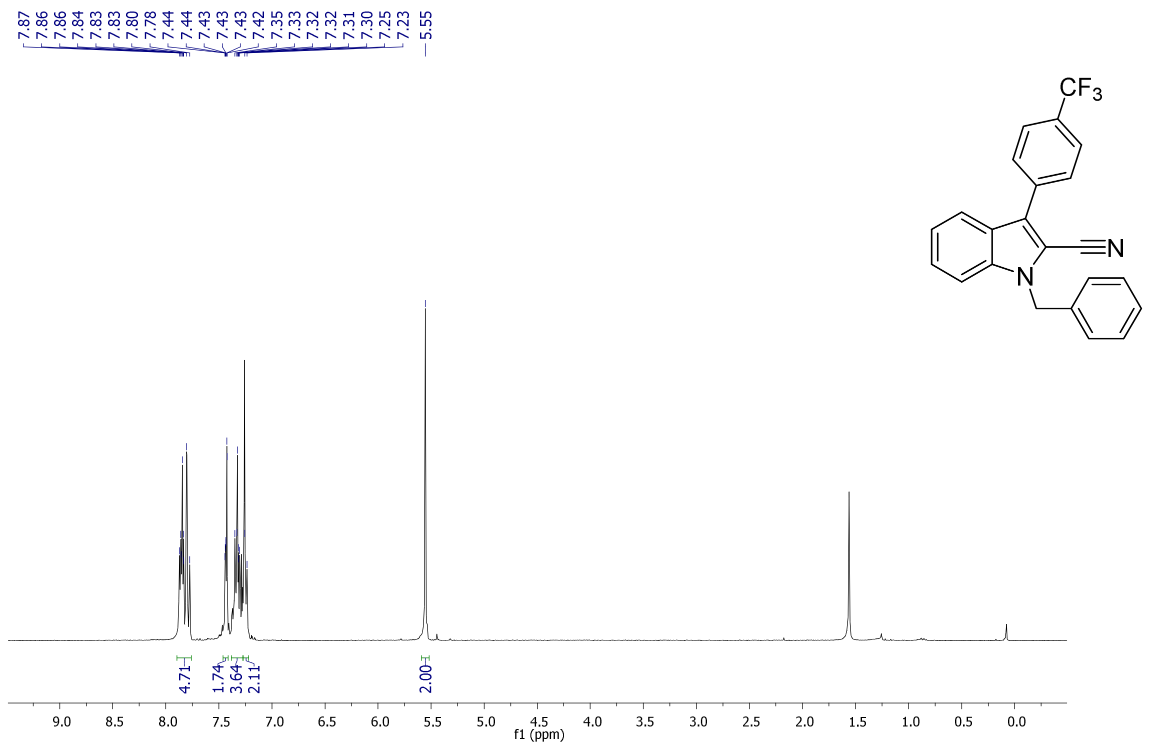

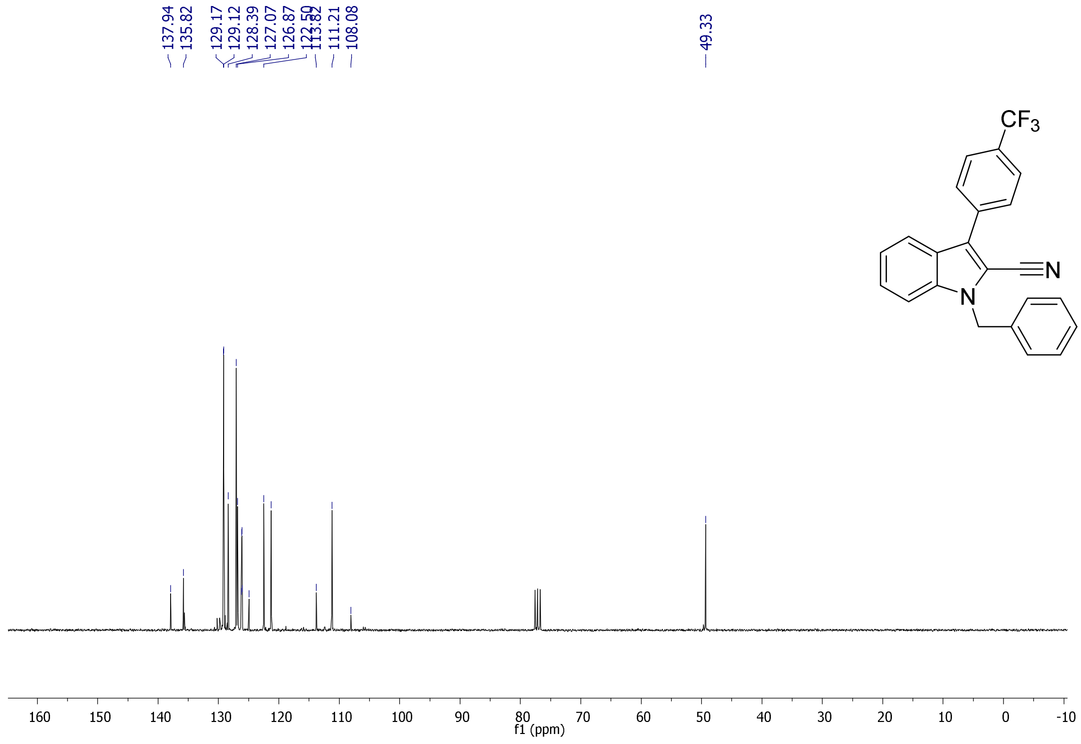

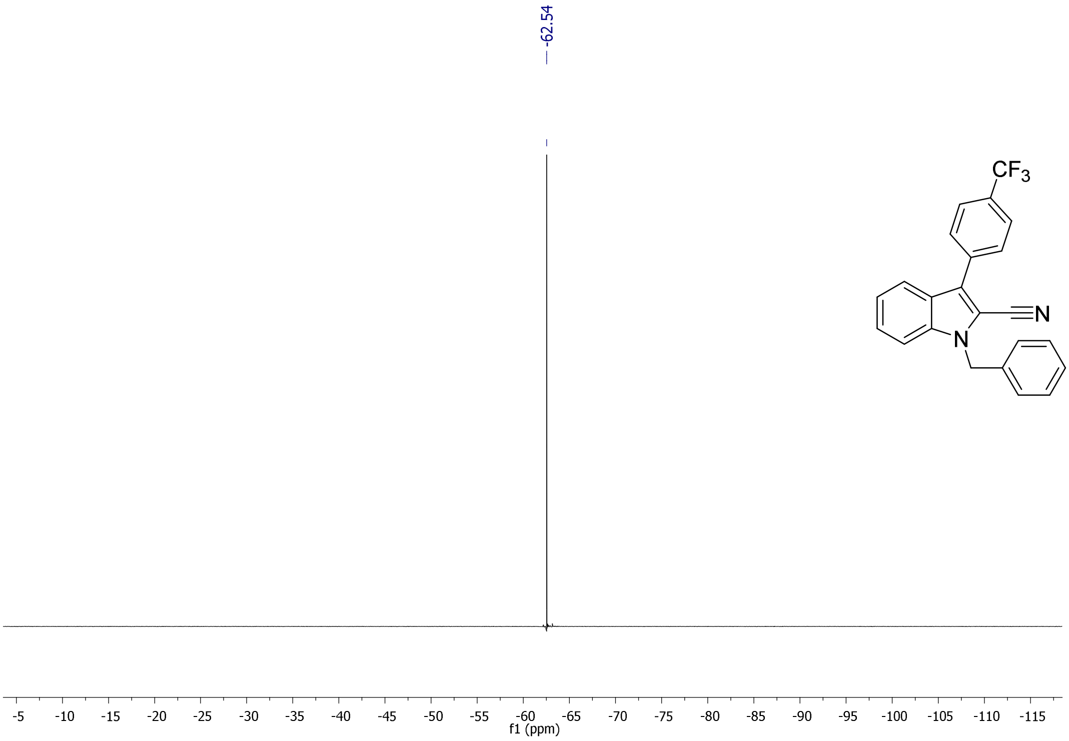
-62.54
CF3
N
N
-5 -10 -15 -20 -25 -30 -35 -40 -45 -50 -55 -60 -65 -70 -75 -80 -85 -90 -95 -100 -105 -110 -115
f1 (ppm)

1-Benzyl-3-(2-methoxyphenyl)-1*H*-indole-2-carbonitrile (9f)

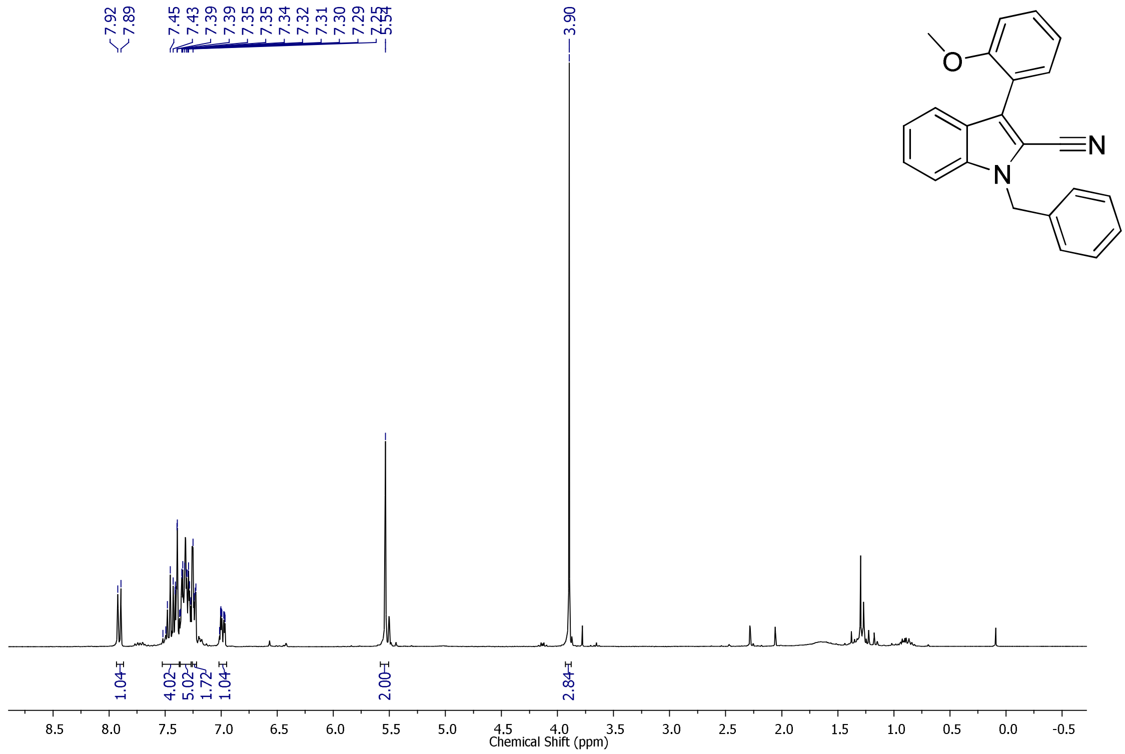

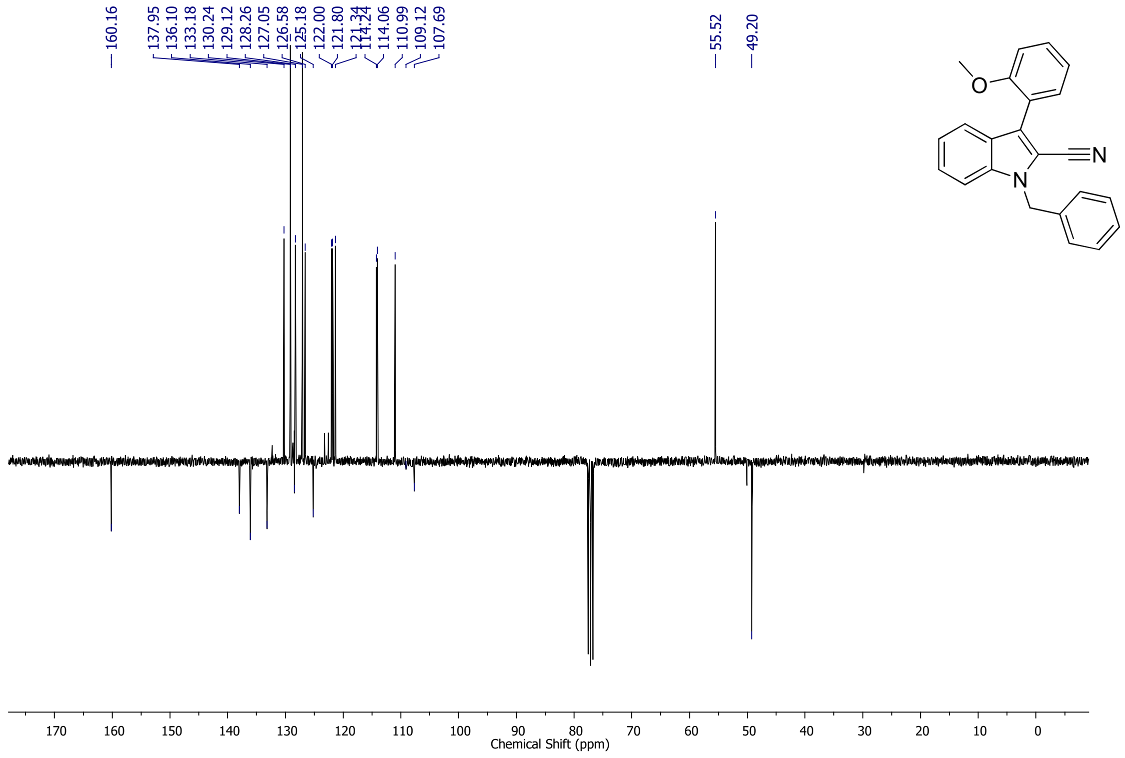

1-Benzyl-3-(2-fluorophenyl)-1*H*-indole-2-carbonitrile (9g)

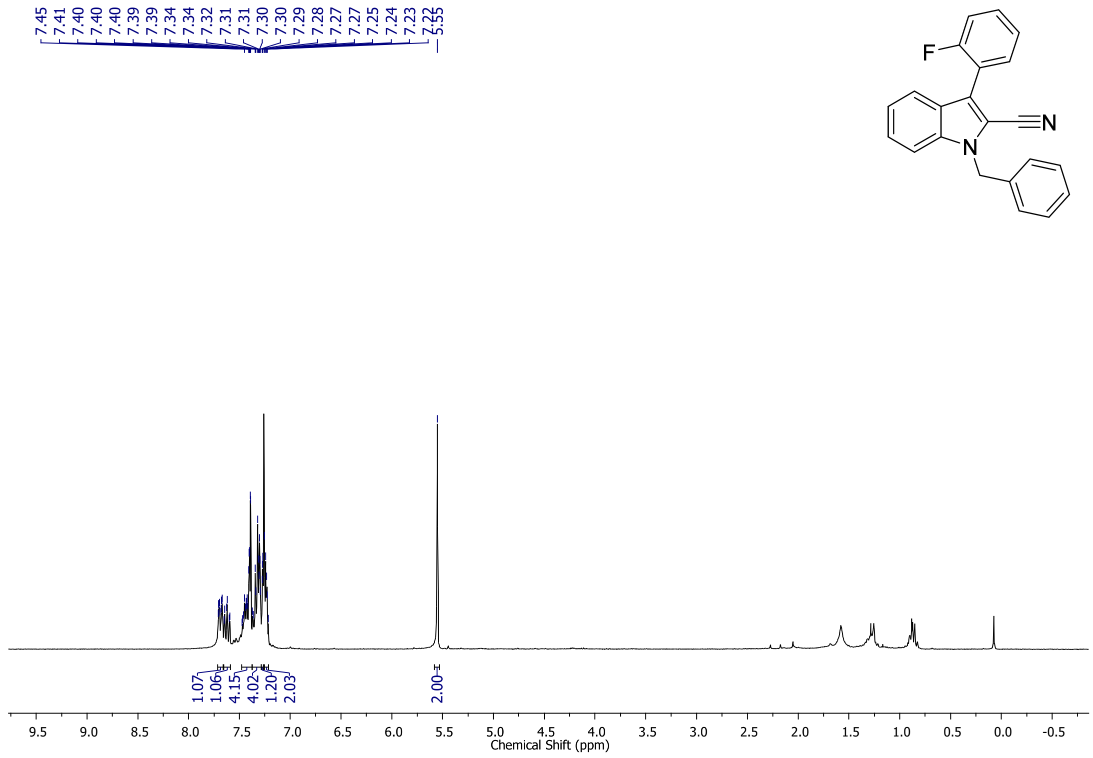

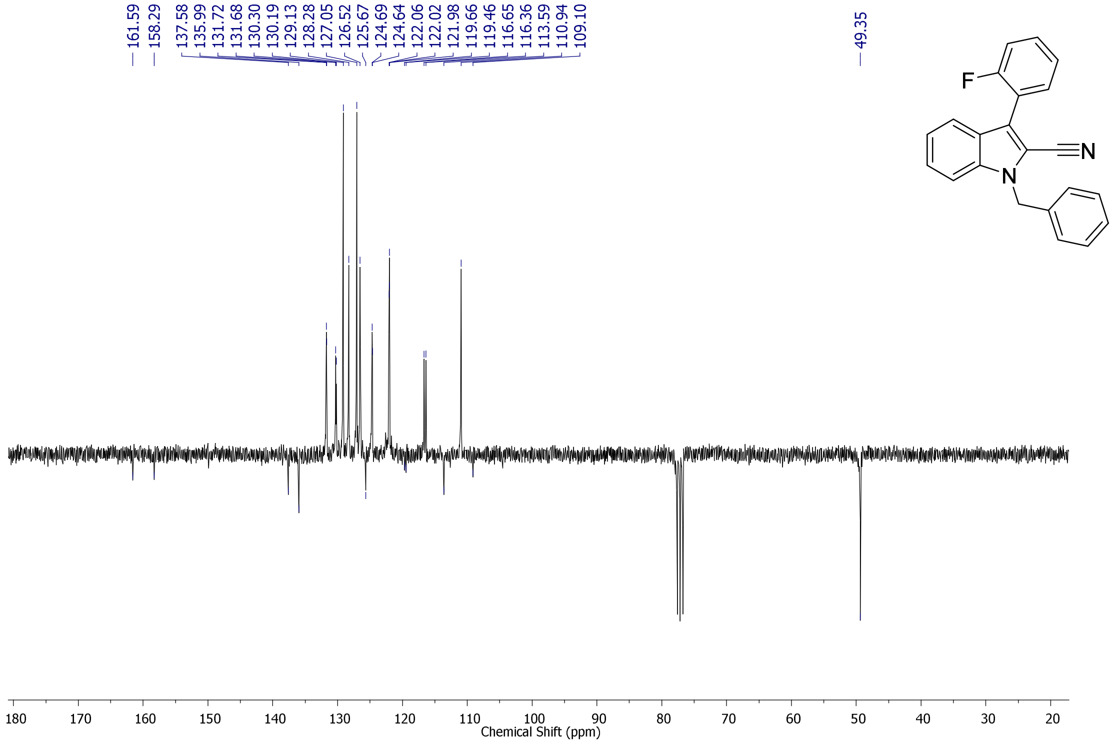

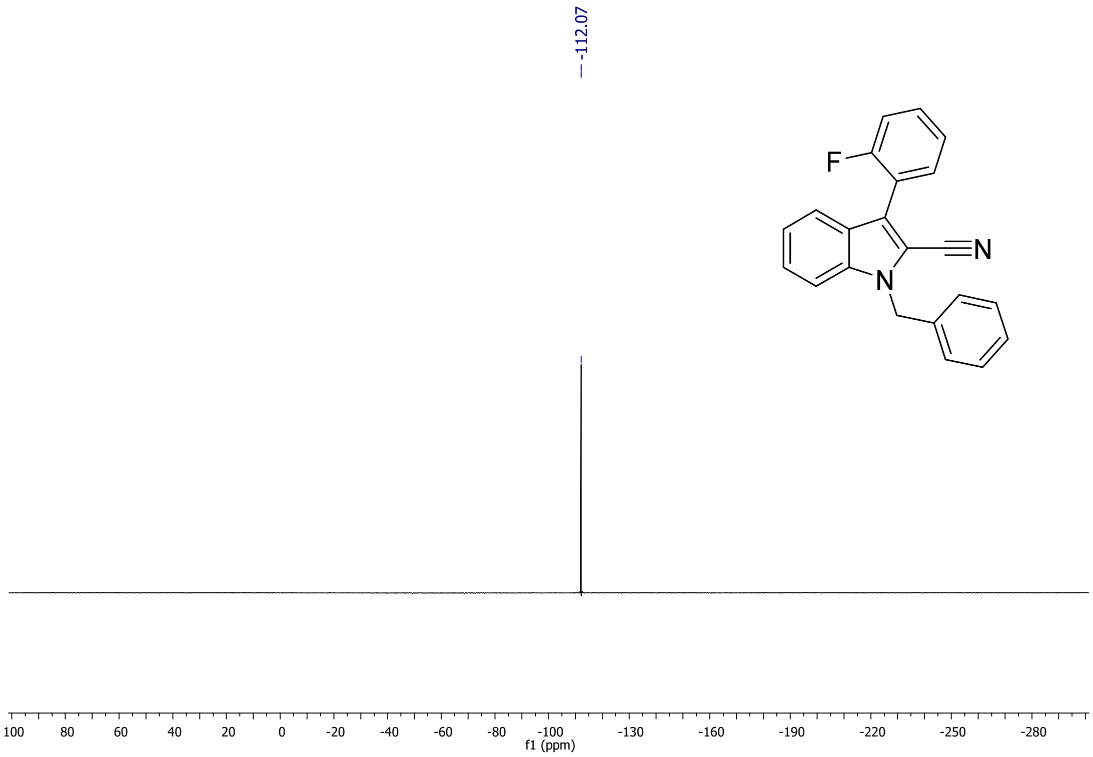
-112.07
F
N
N
100
80
60
40
20
0
-20
-40
-60
-80
-100
-130
-160
-190
-220
-250
-280
f1 (ppm)

1-Benzyl-3-(*o*-tolyl)-1*H*-indole-2-carbonitrile (9h)

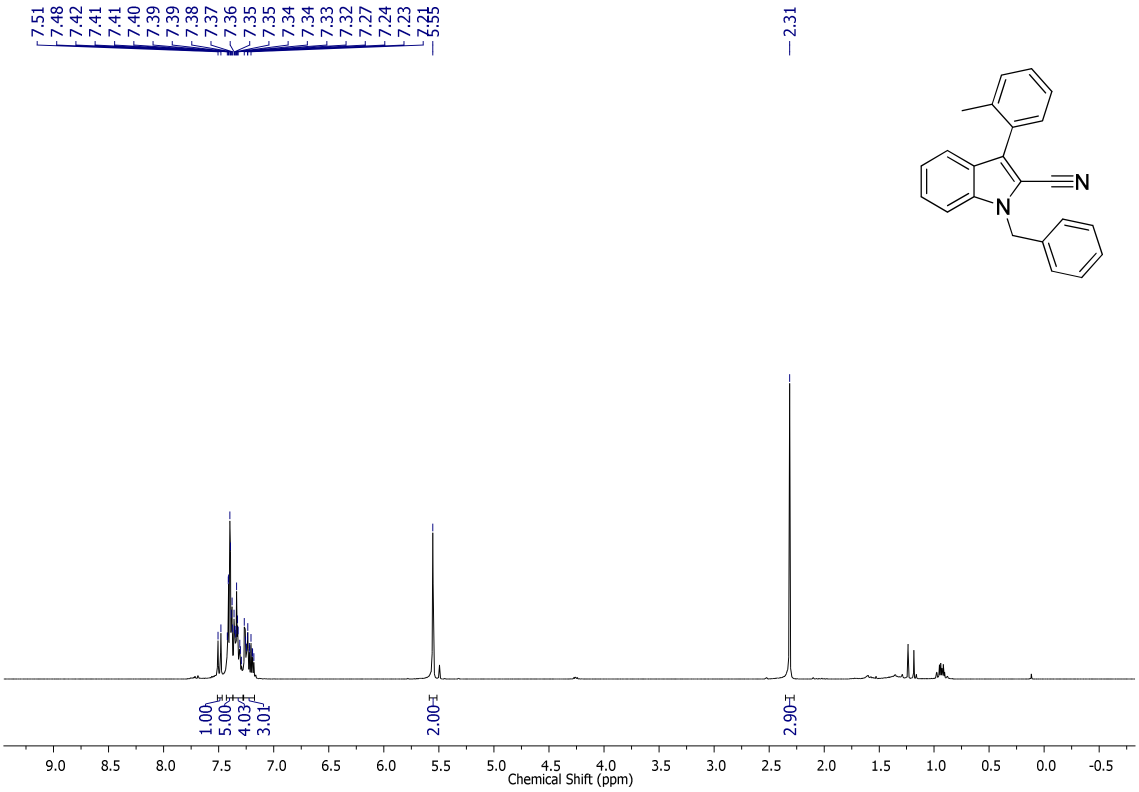

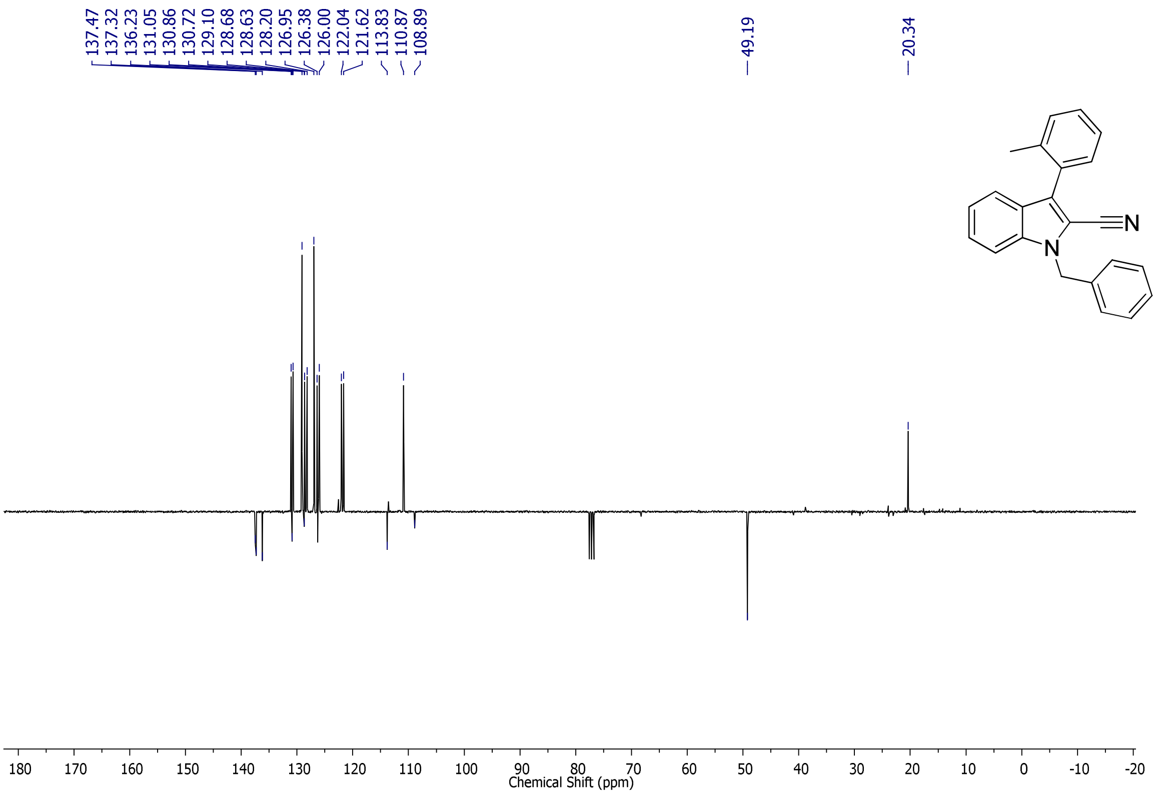

1-Benzyl-3-(3-methoxyphenyl)-1*H*-indole-2-carbonitrile (9i)

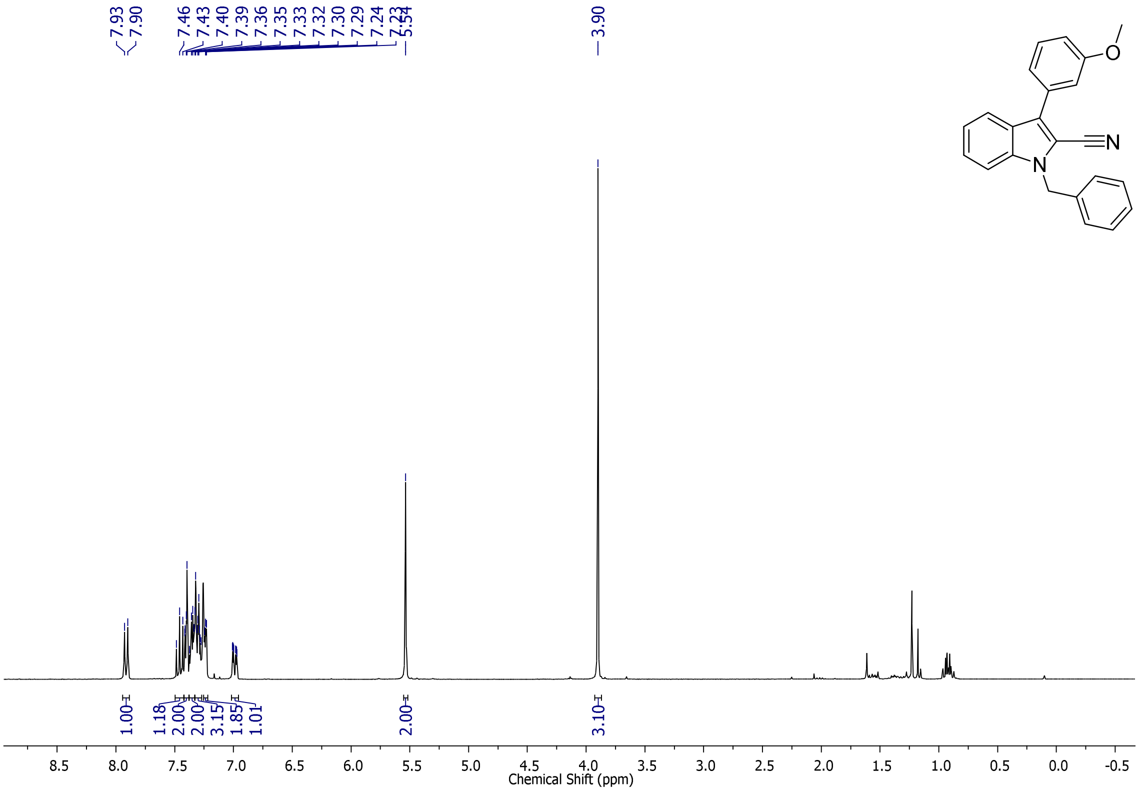

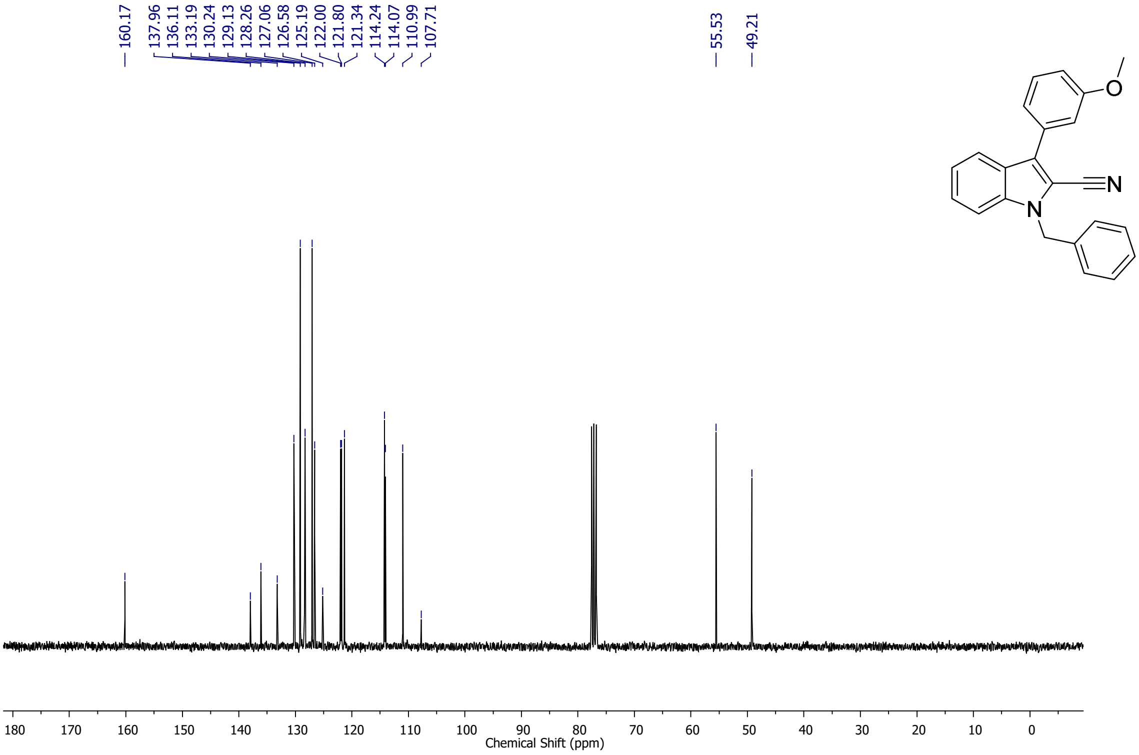

1-Benzyl-3-(2,4-dimethoxyphenyl)-1*H*-indole-2-carbonitrile (9j)

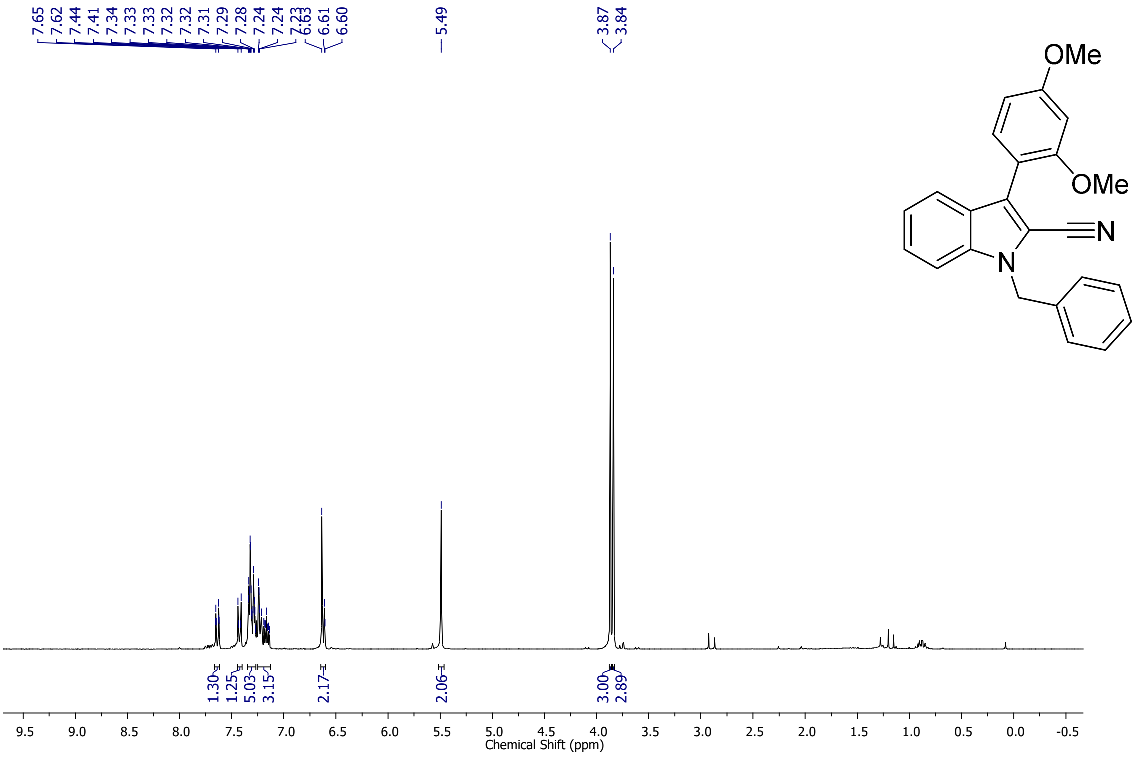

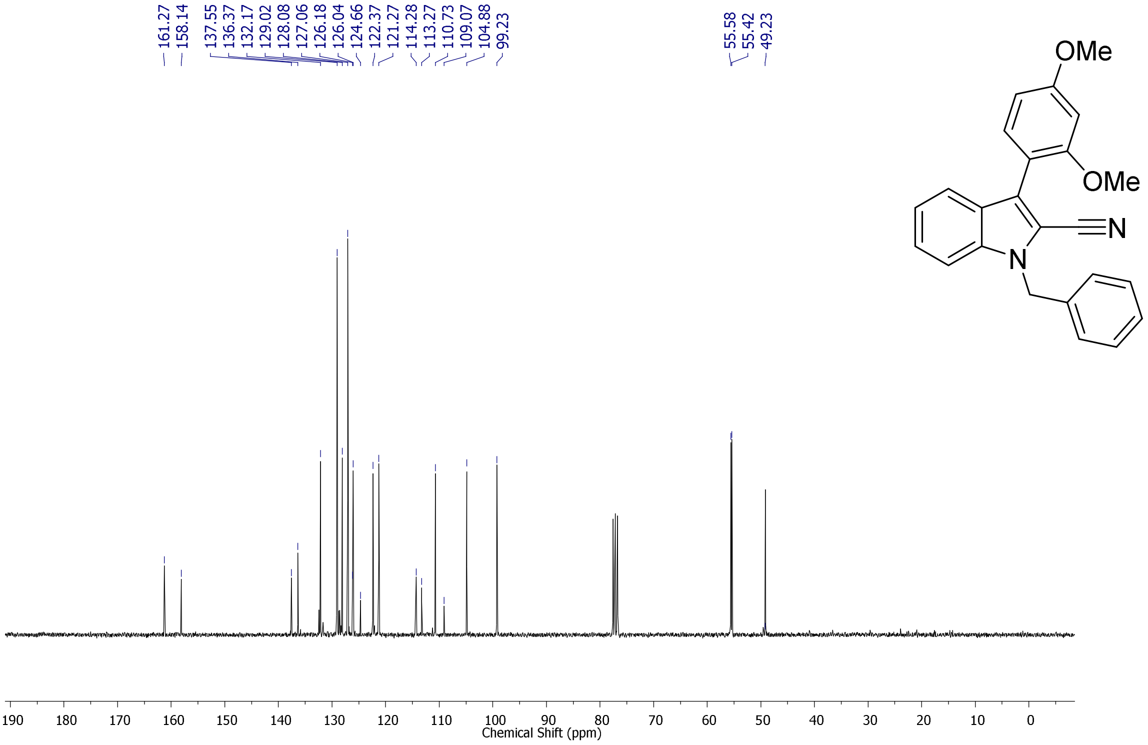

1-Benzyl-3-(2,3-dihydrobenzo[*b*][1,4]dioxin-6-yl)-1*H*-indole-2-carbonitrile (9k)

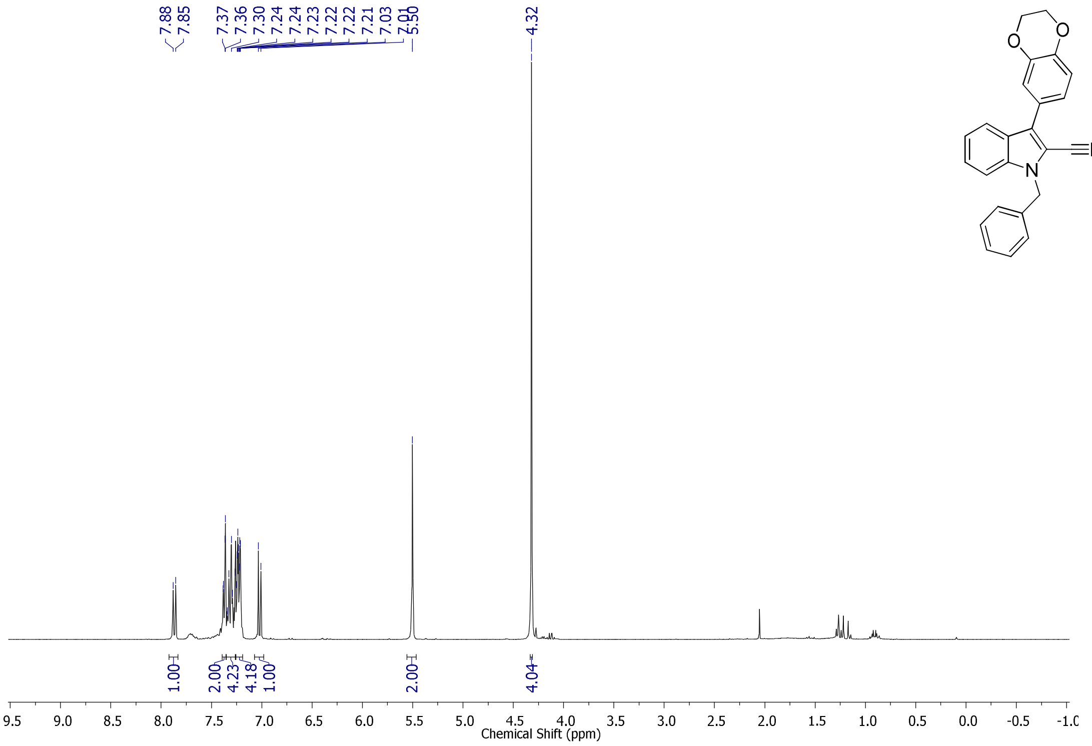

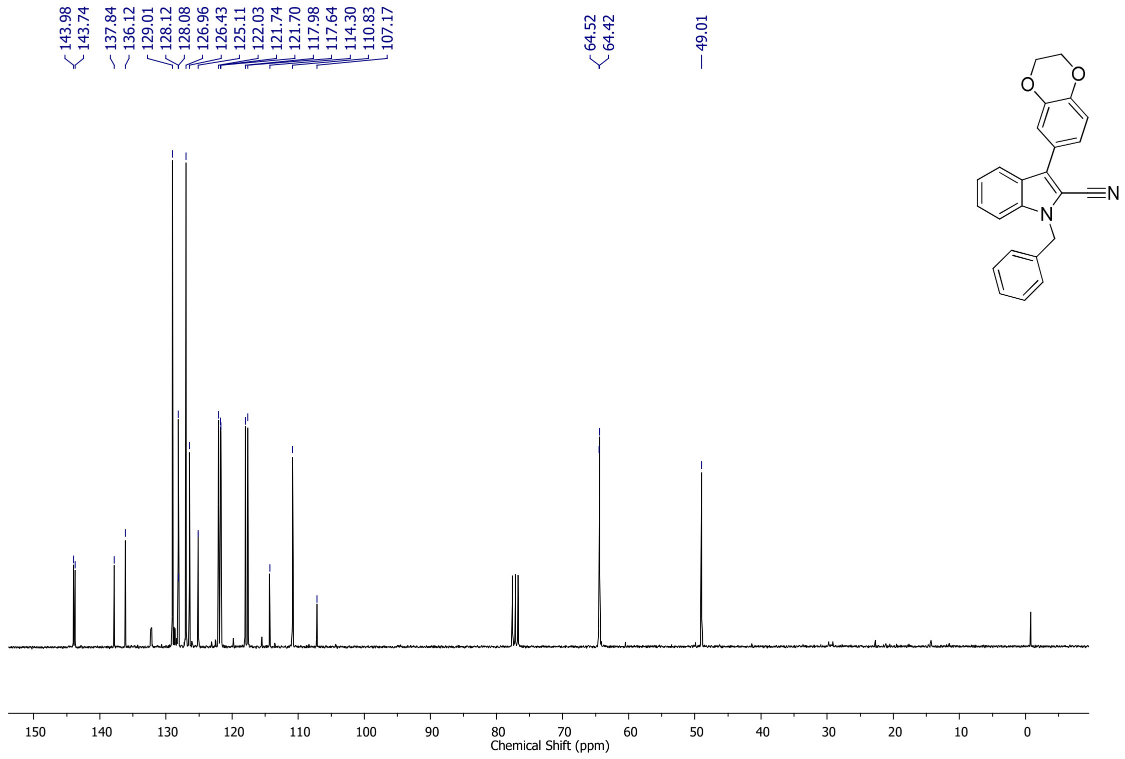

1-Benzyl-3-(6-methoxynaphthalen-2-yl)-1*H*-indole-2-carbonitrile (9l)

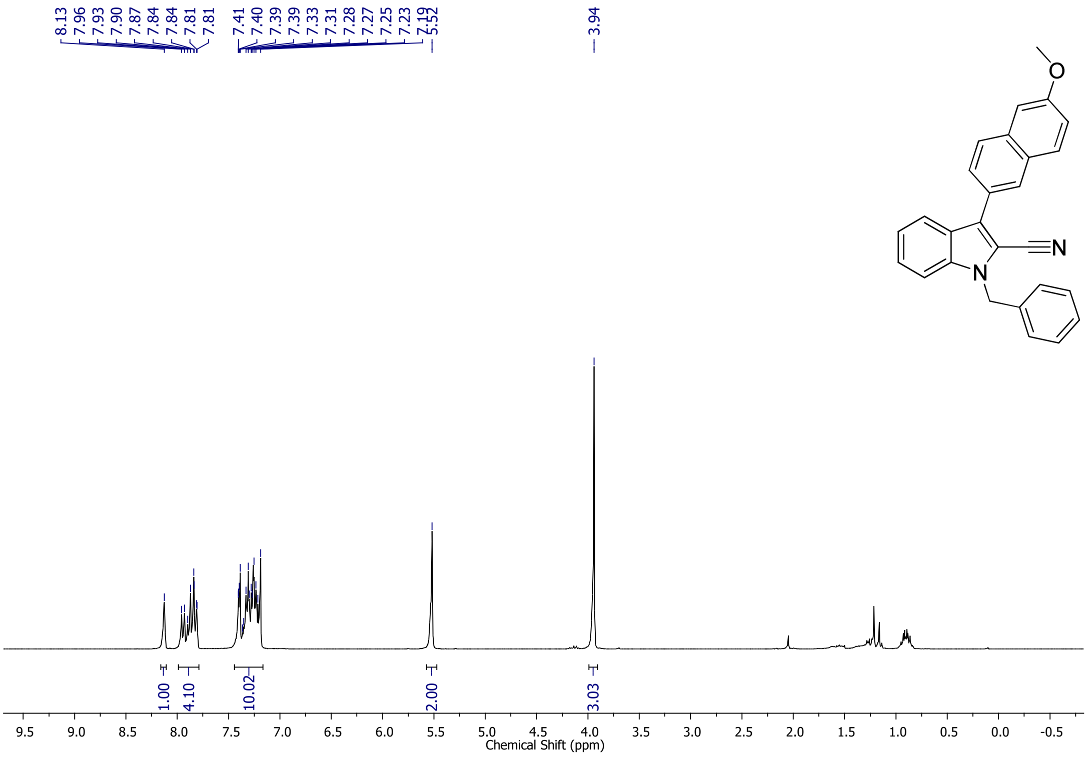

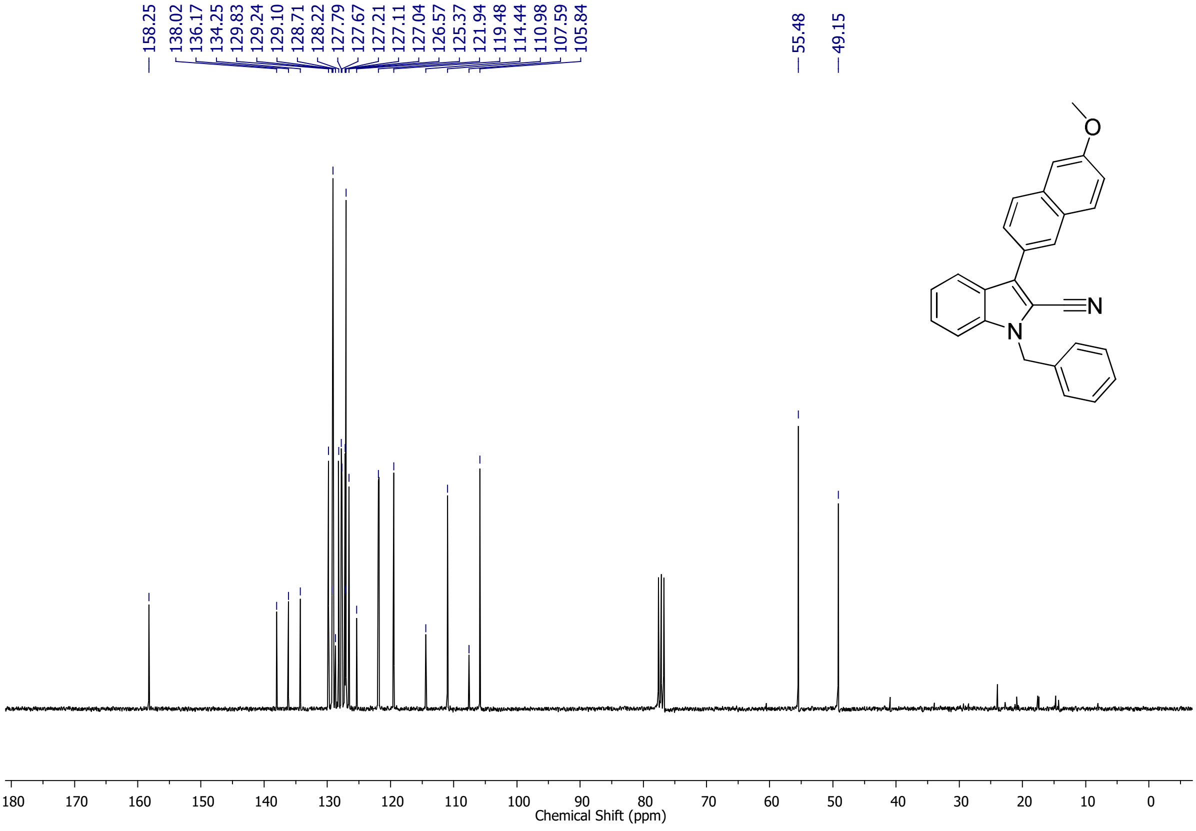

1-Benzyl-3-(2-methoxypyridin-3-yl)-1*H*-indole-2-carbonitrile (9m)

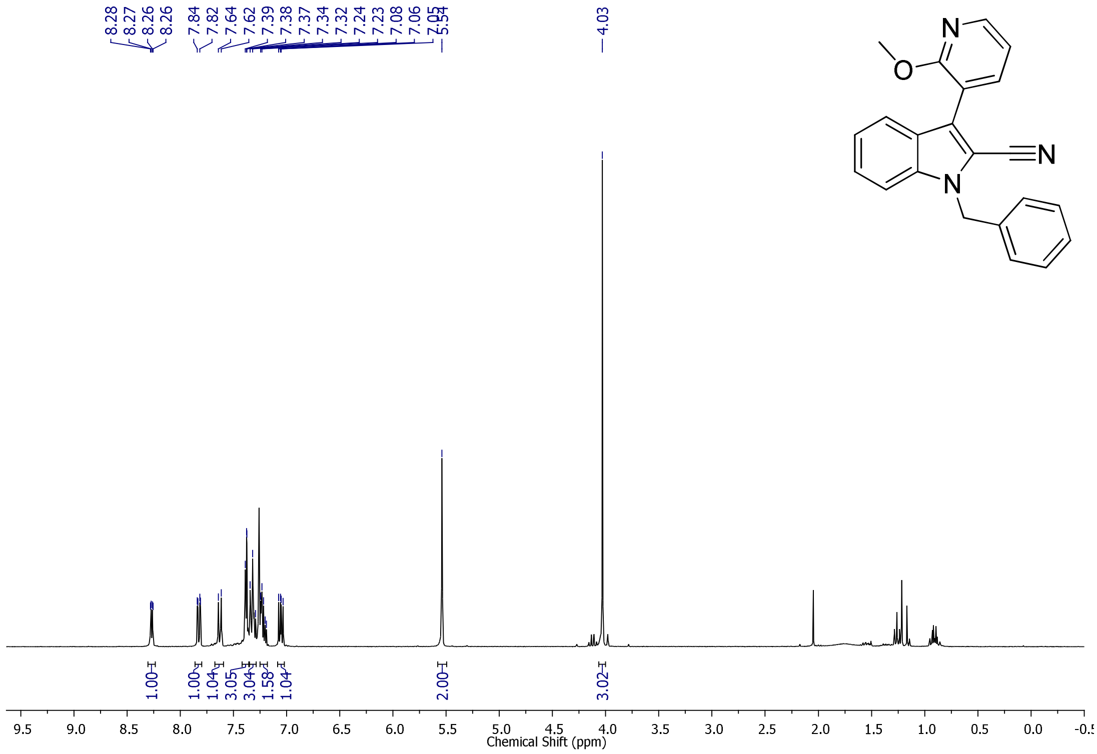

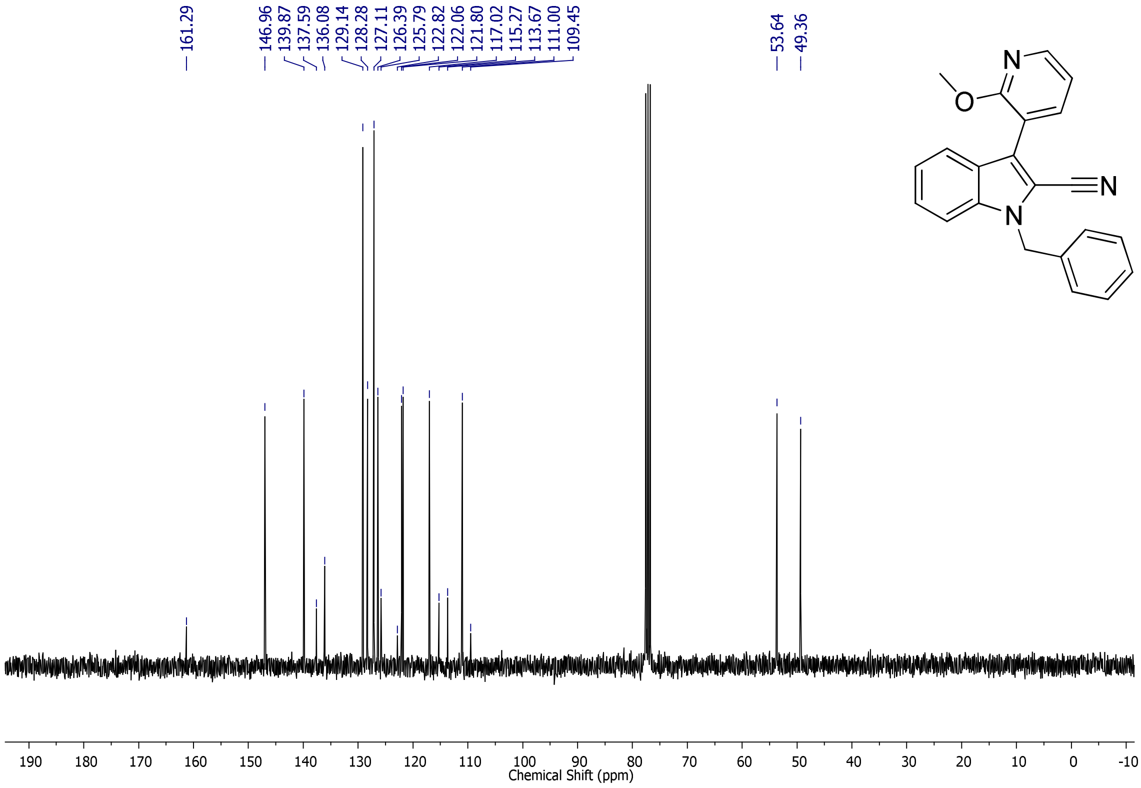

1-Benzyl-5-methoxy-3-(4-methoxyphenyl)-1*H*-indole-2-carbonitrile (9n)

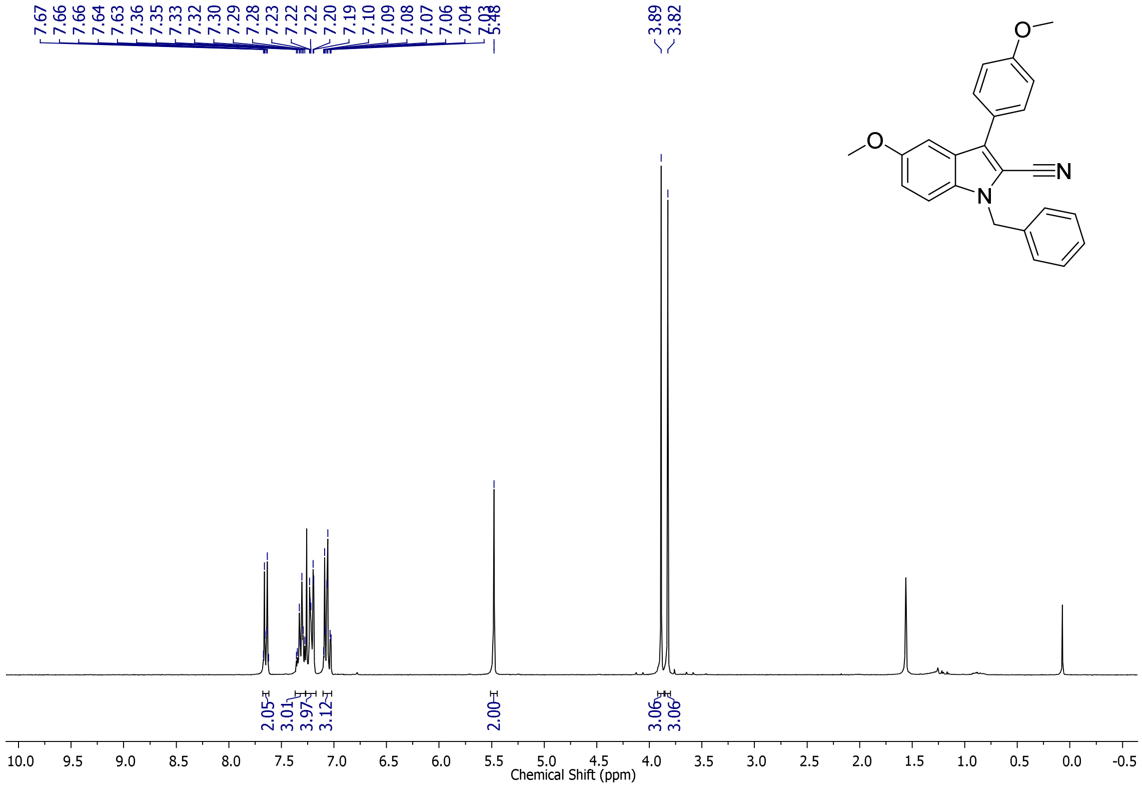

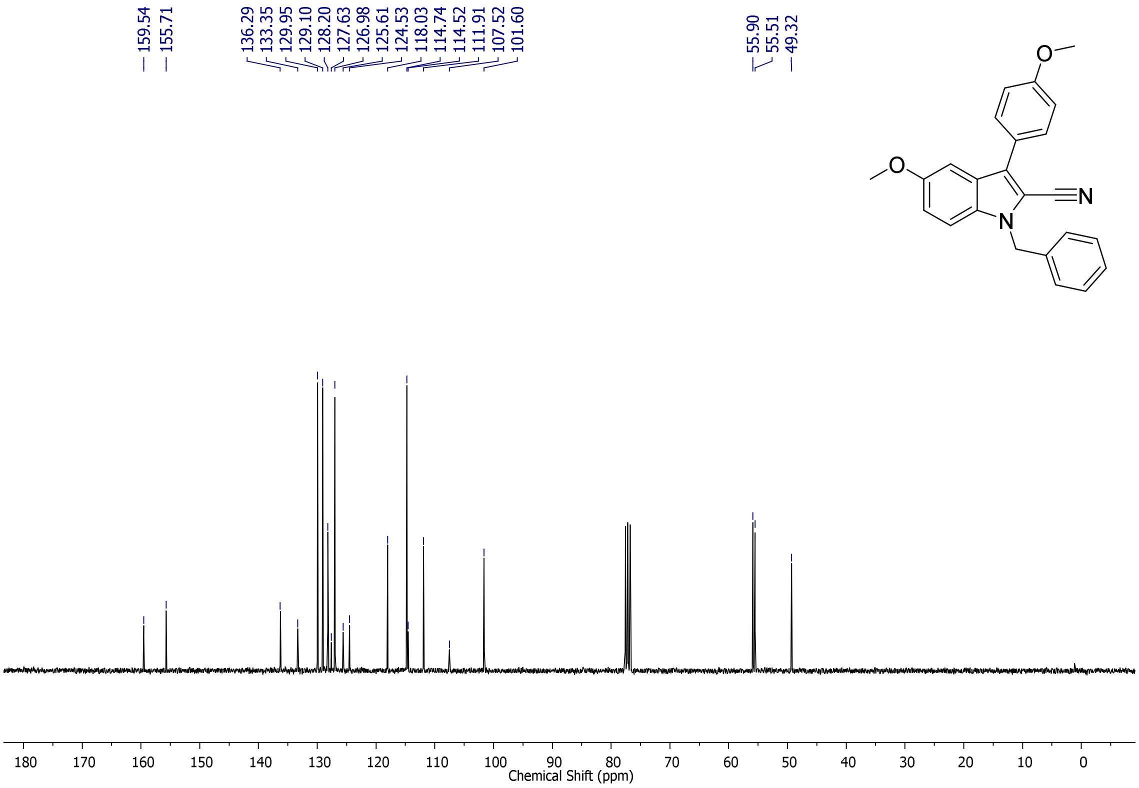

1-Benzyl-6-methoxy-3-(3-methoxyphenyl)-1*H*-indole-2-carbonitrile (9o)

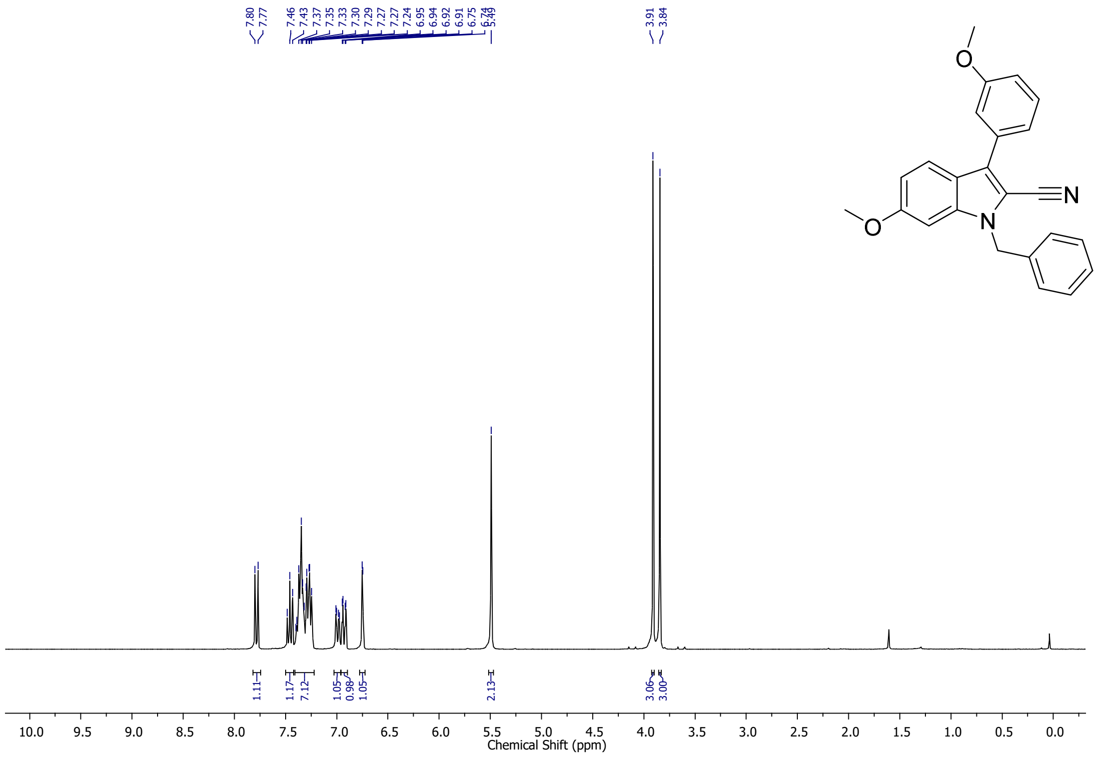

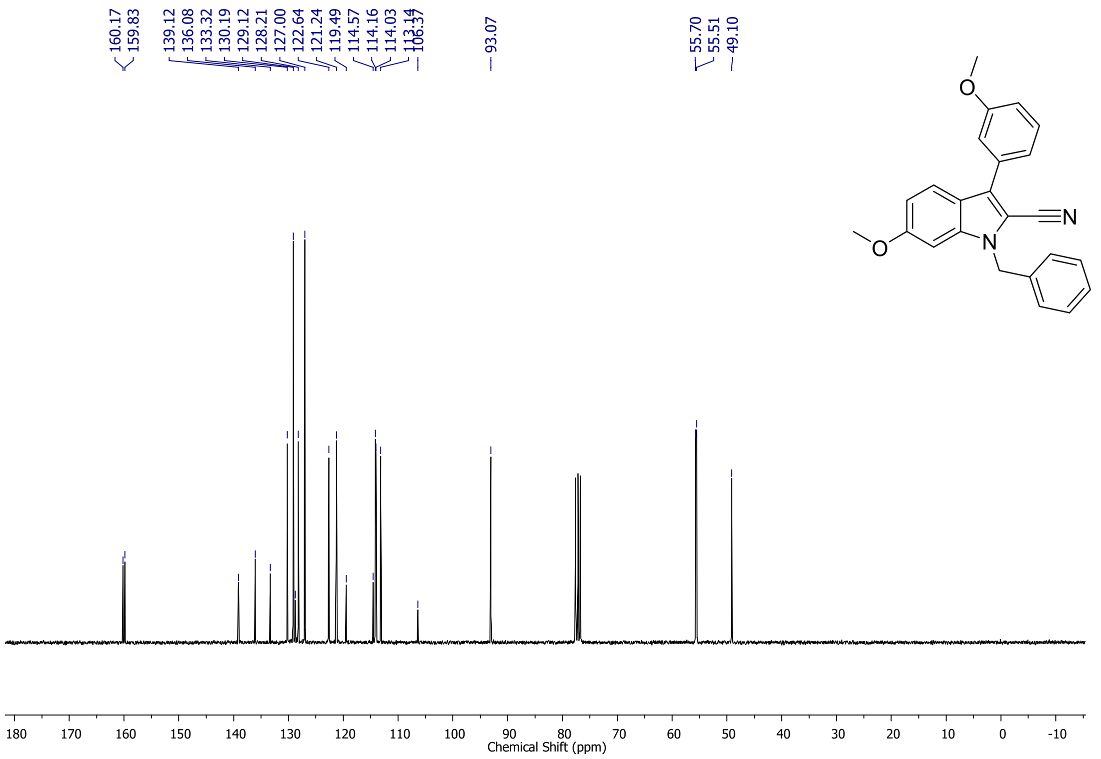

1-Benzyl-5-fluoro-3-(3-methoxyphenyl)-1*H*-indole-2-carbonitrile (9p)

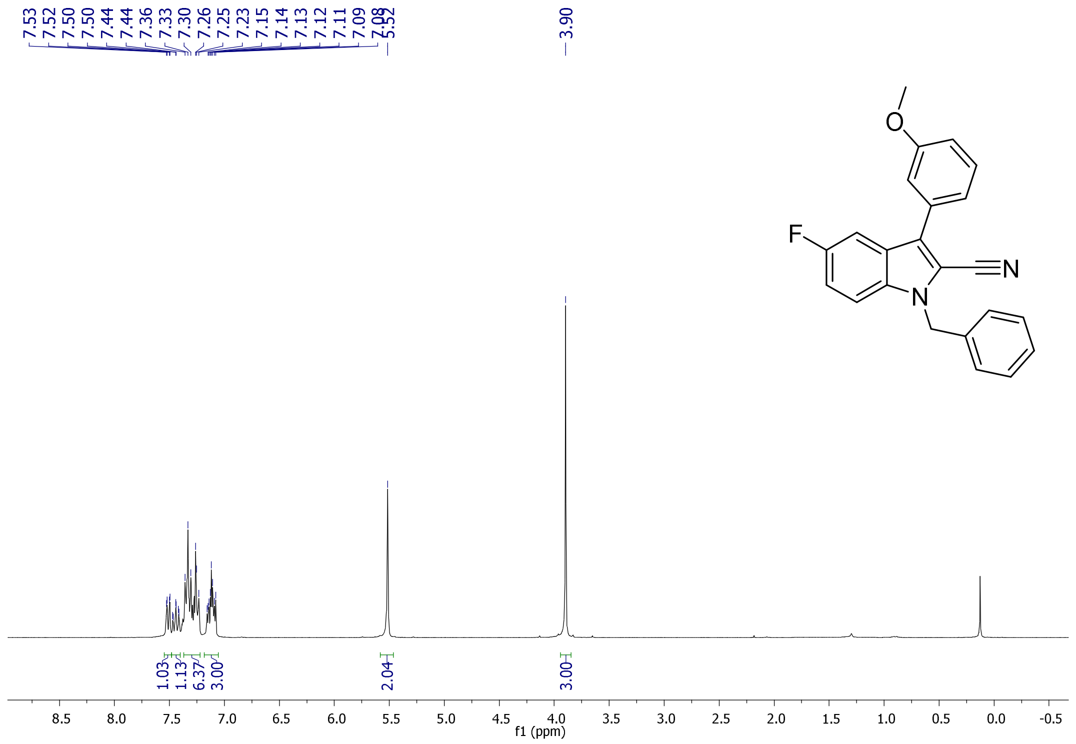

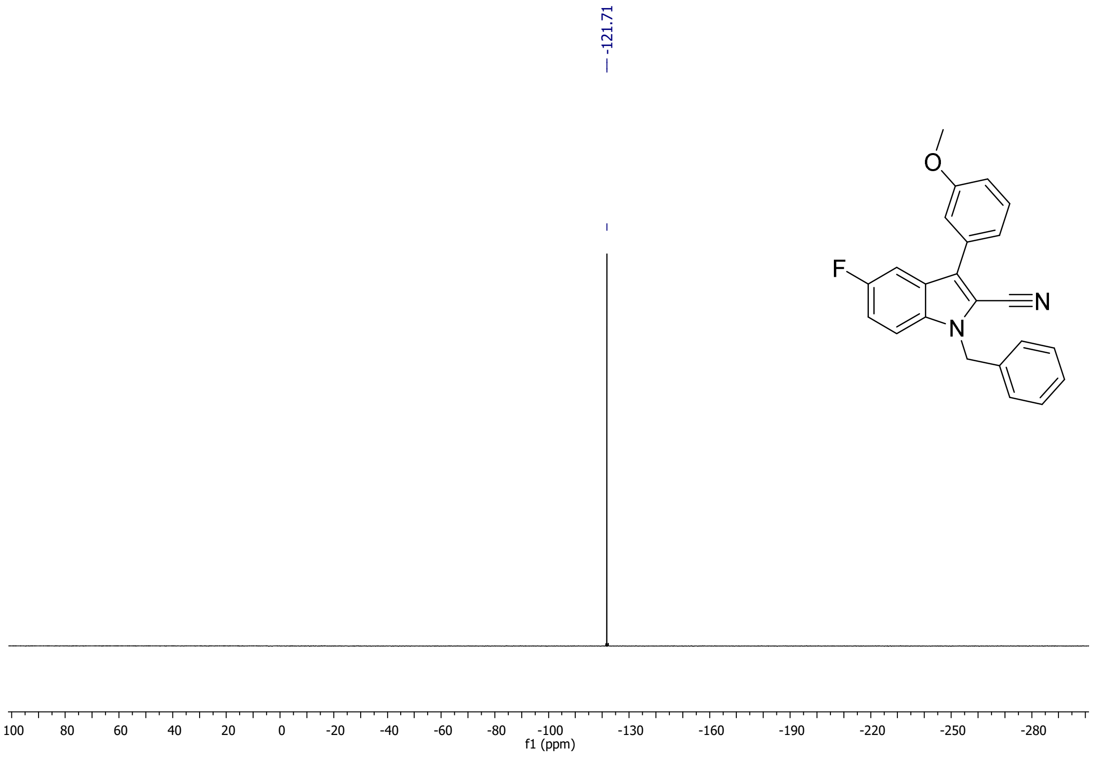

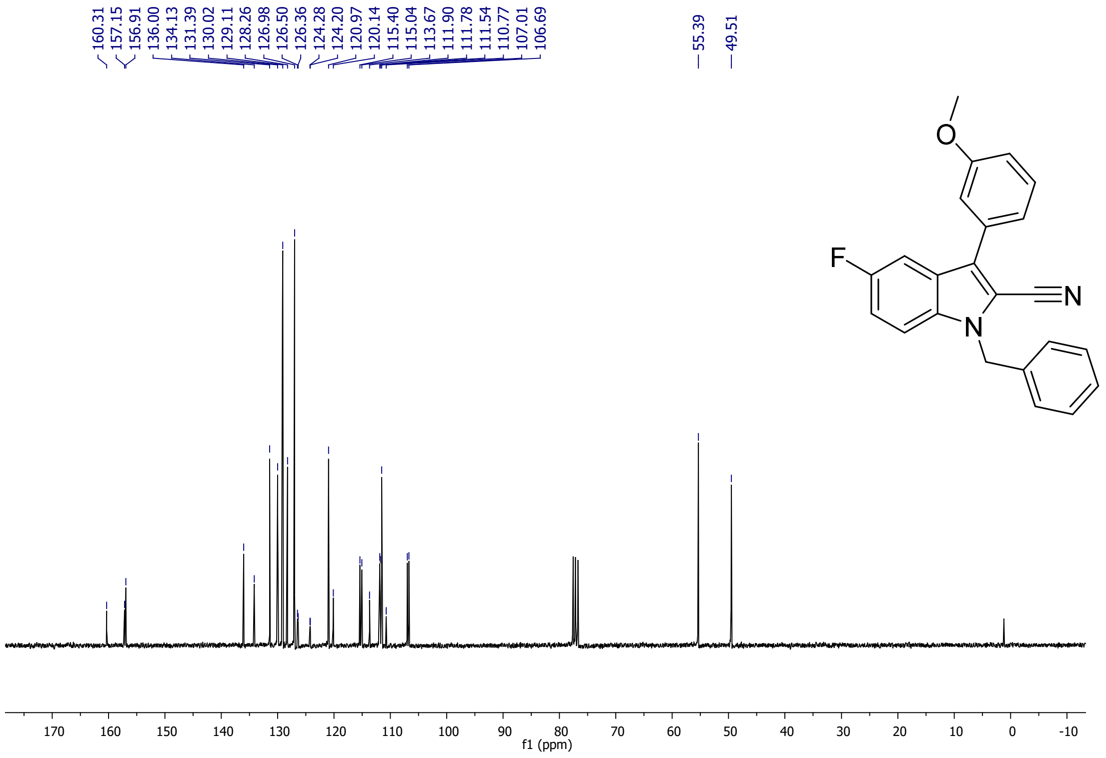

160.31
157.15
156.91
136.00
134.13
131.39
130.02
129.11
128.26
126.98
126.50
126.36
124.28
124.20
120.97
120.14
115.40
115.04
113.67
111.90
111.78
111.54
110.77
107.01
106.69
55.39
49.51
F
O
N
N
170
160
150
140
130
120
110
100
90
80
70
60
50
40
30
20
10
0
-10
f1 (ppm)

Methyl (*E*)-3-(1-benzyl-2-cyano-1*H*-indol-3-yl)acrylate (10a)

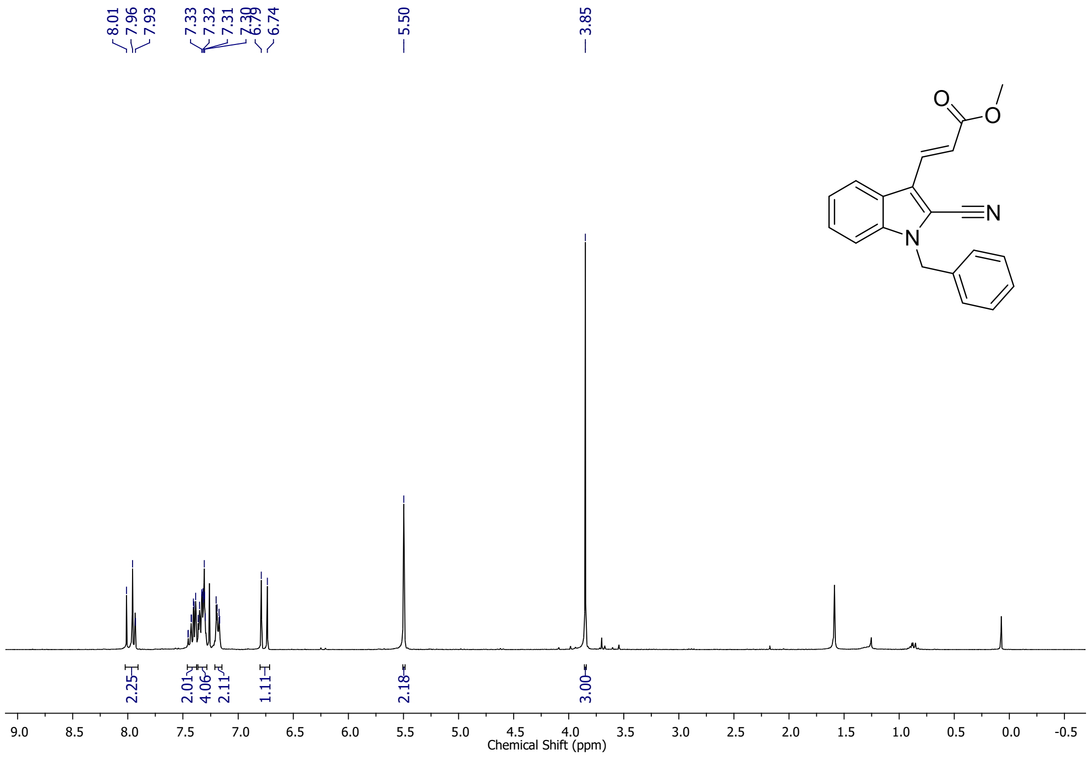

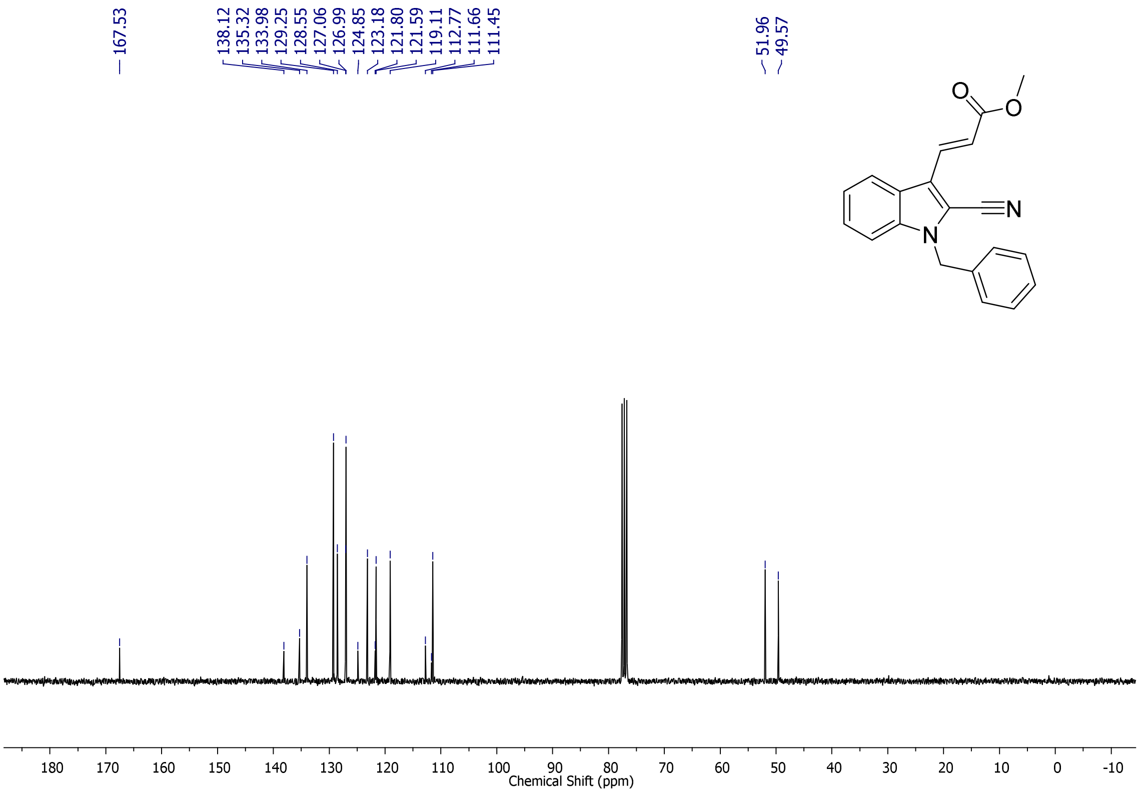

(*E*)-1-Benzyl-3-styryl-1*H*-indole-2-carbonitrile (10b)

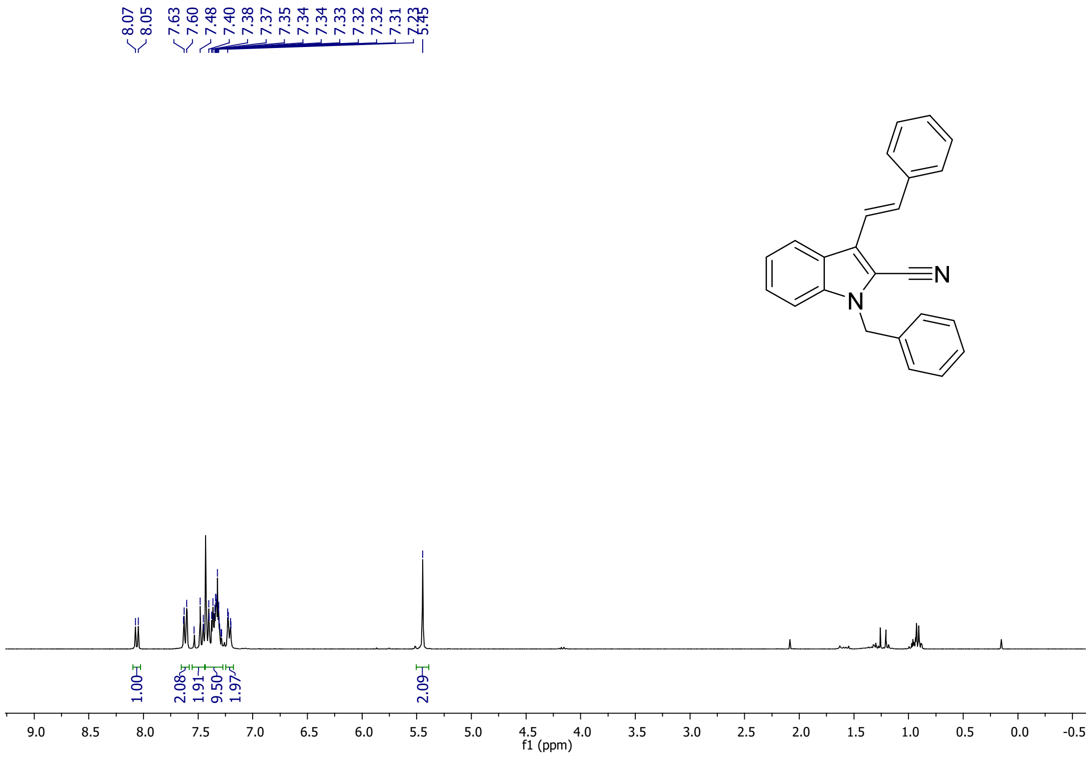

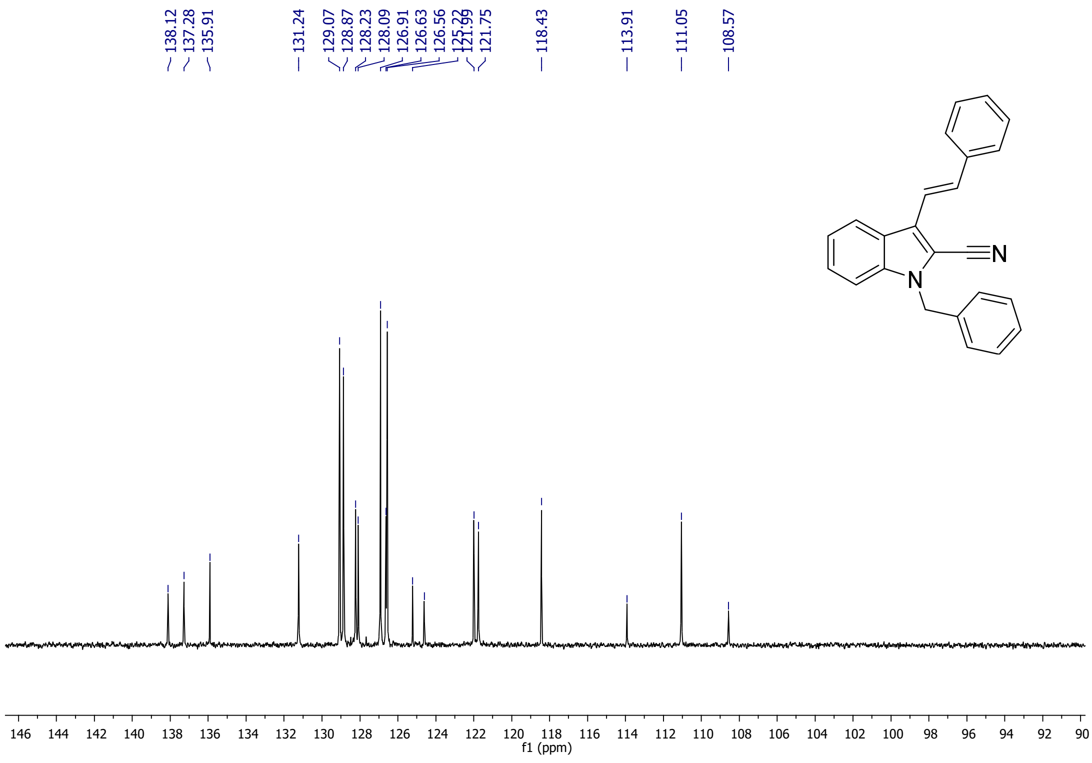

(*E*)-1-Benzyl-3-(3-cyanoprop-1-en-1-yl)-1*H*-indole-2-carbonitrile (10c)

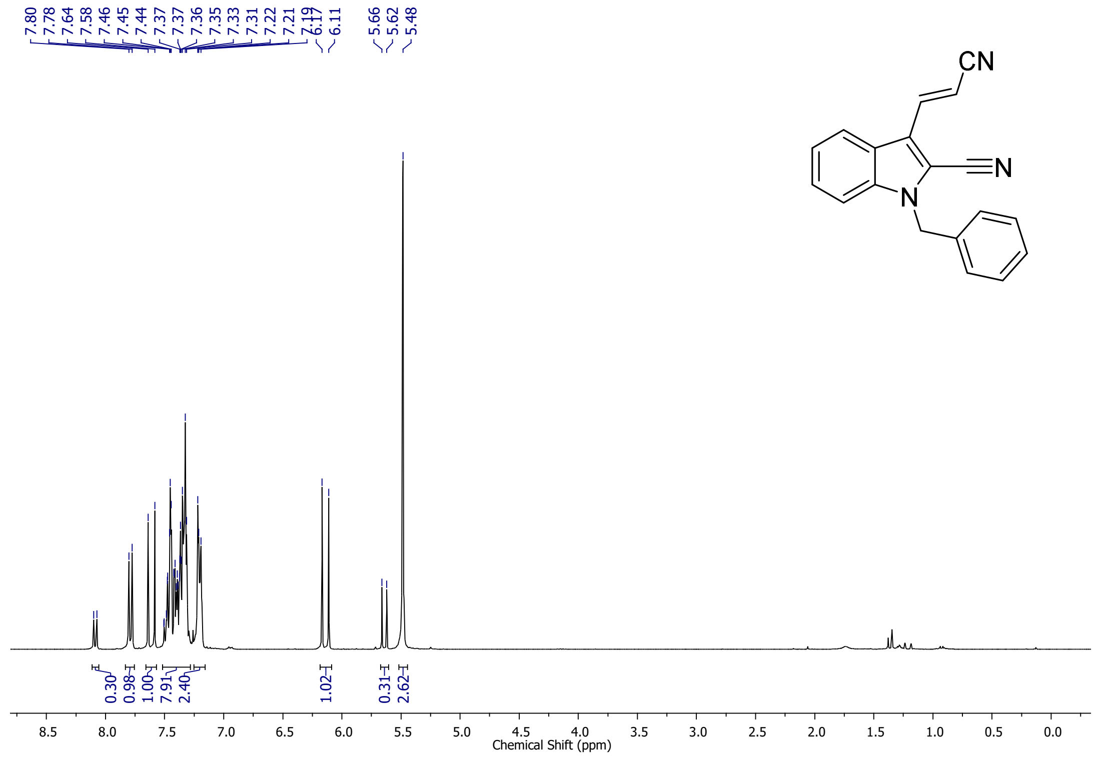

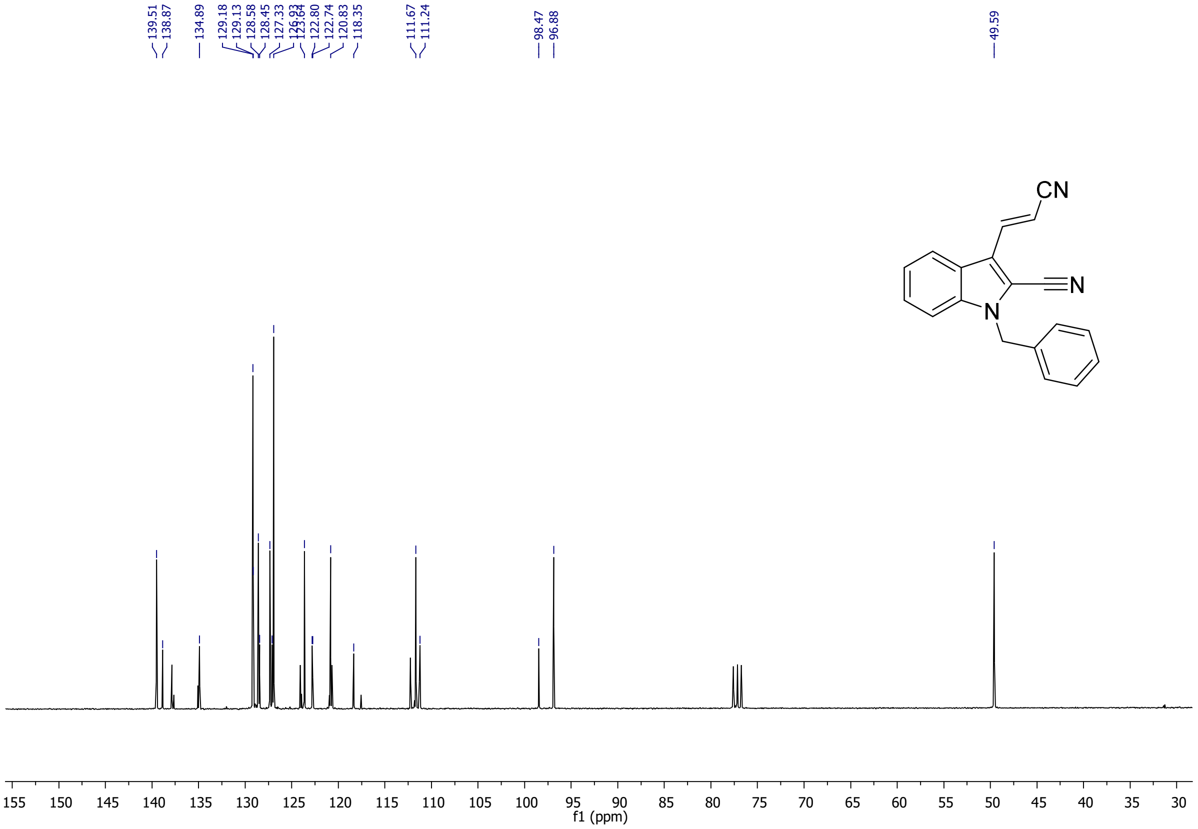

(*E*)-Methyl-3-(1-benzyl-2-cyano-5-methoxy-1*H*-indol-3-yl)acrylate (10d)

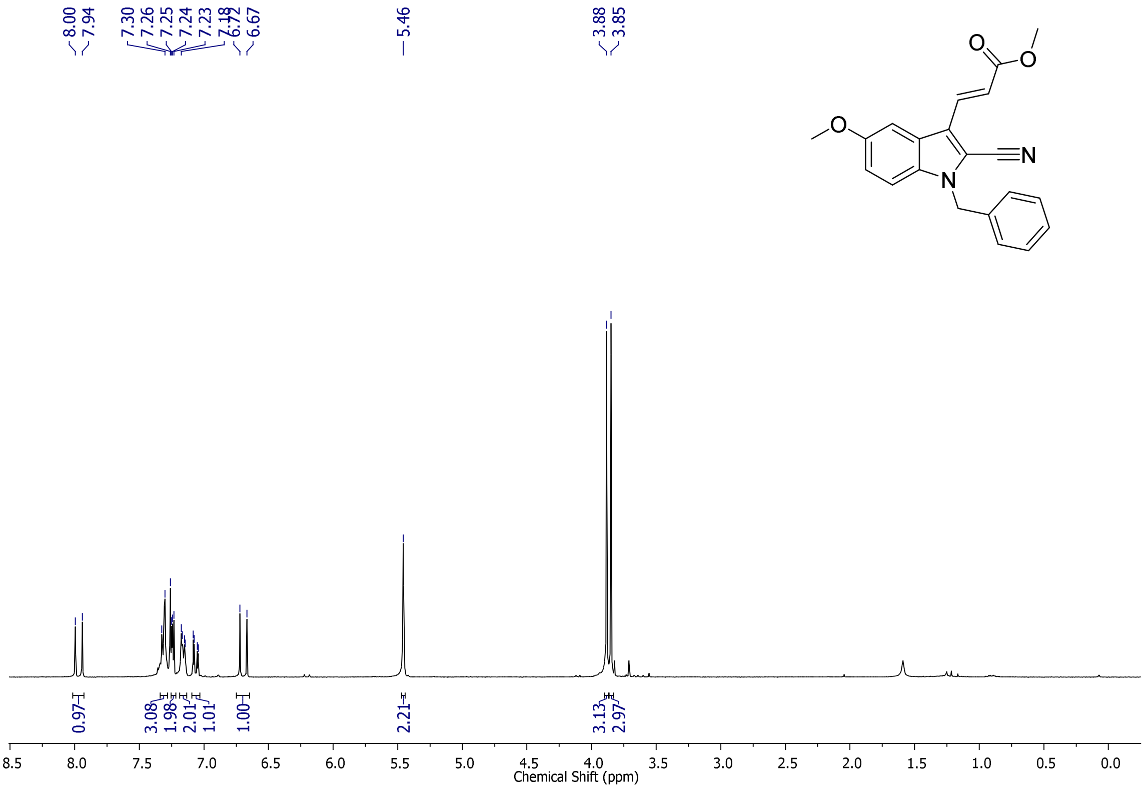

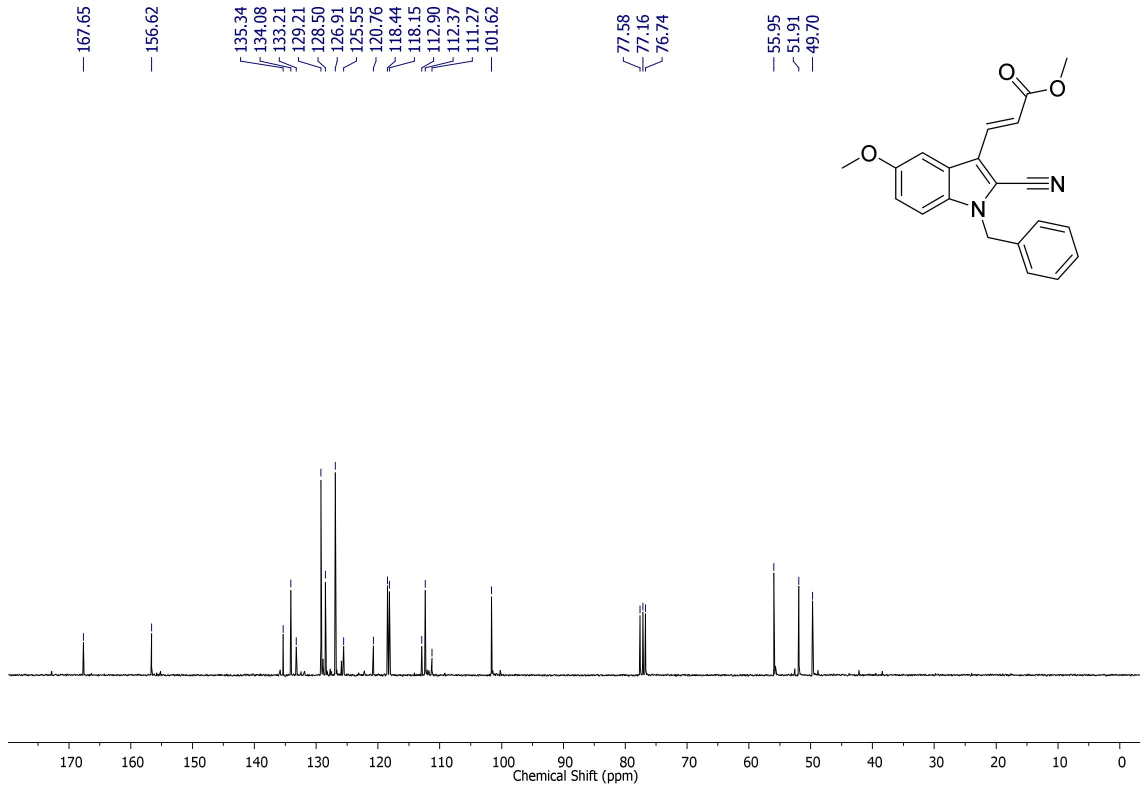

(*E*)-Methyl-3-(1-benzyl-2-cyano-6-methoxy-1*H*-indol-3-yl)acrylate (10e)

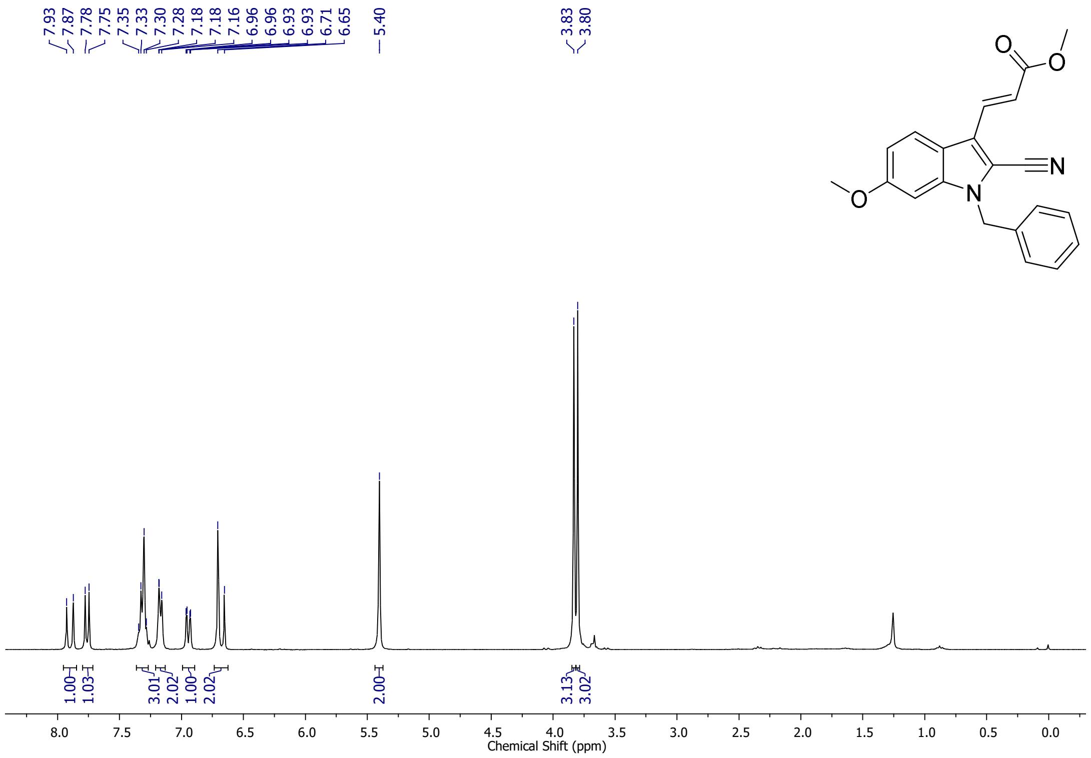

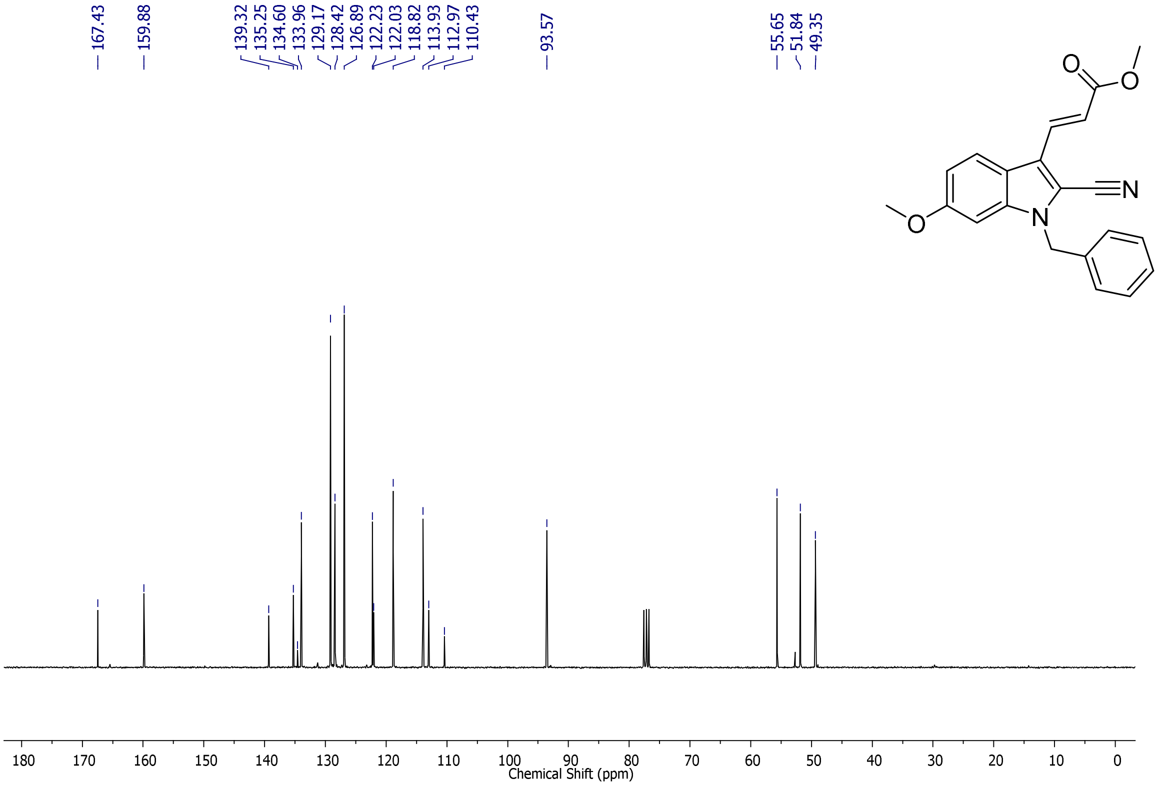

(*E*)-Methyl 3-(1-benzyl-2-cyano-5-fluoro-1*H*-indol-3-yl)acrylate (10f)

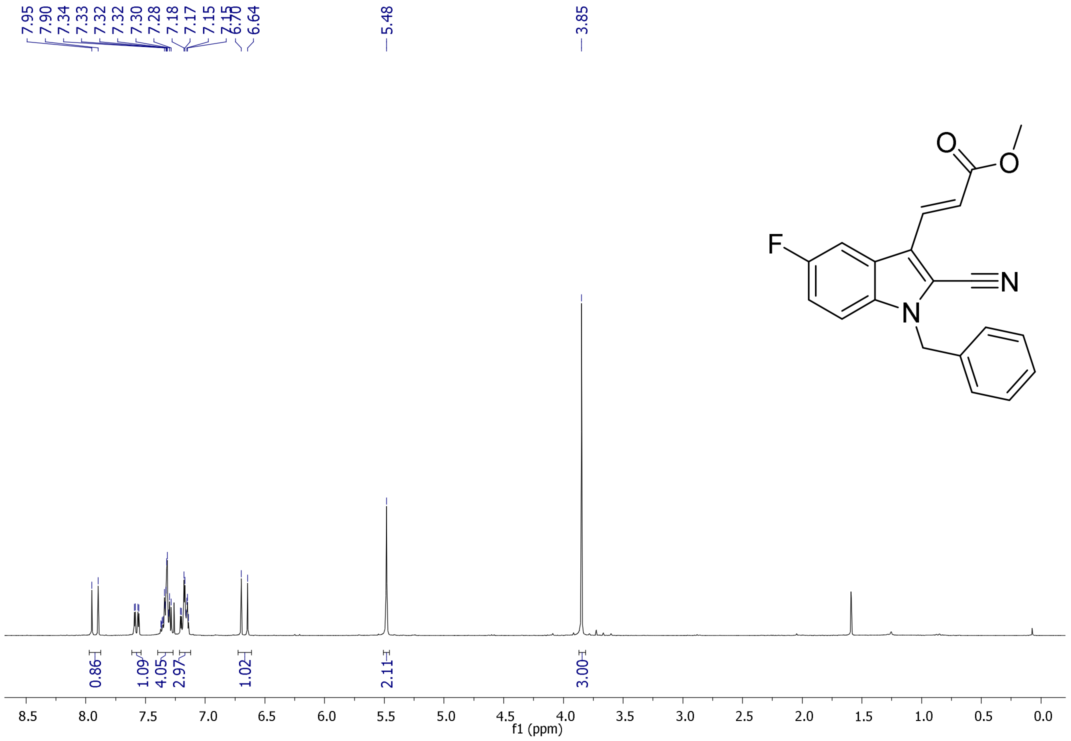

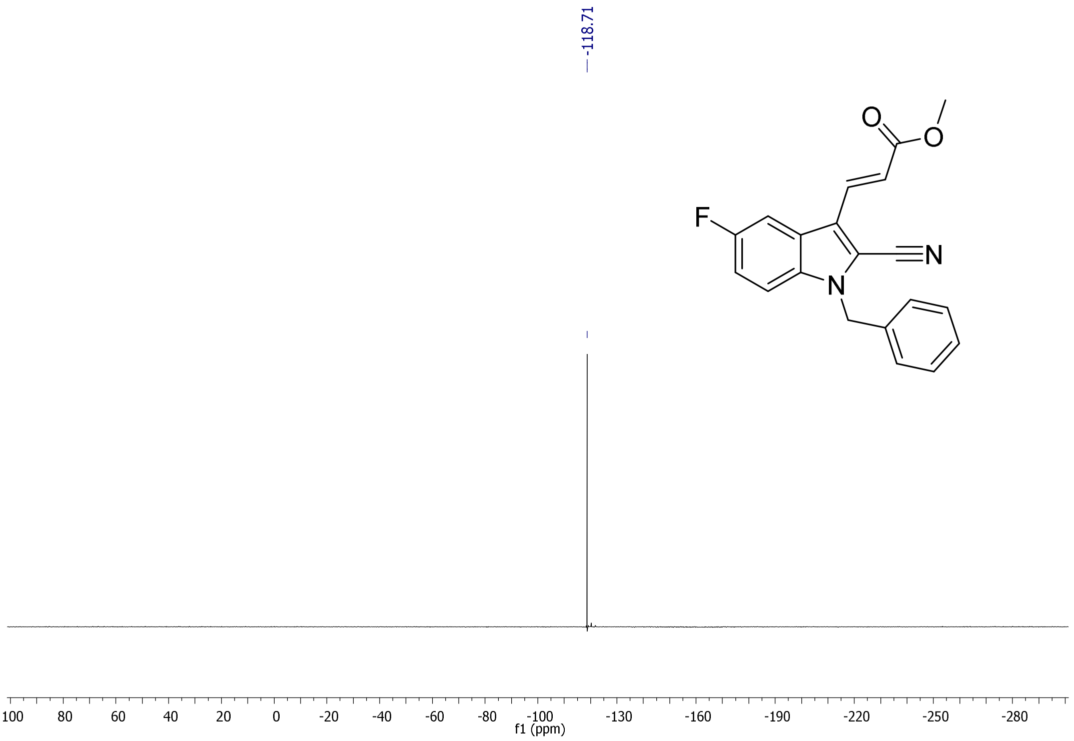

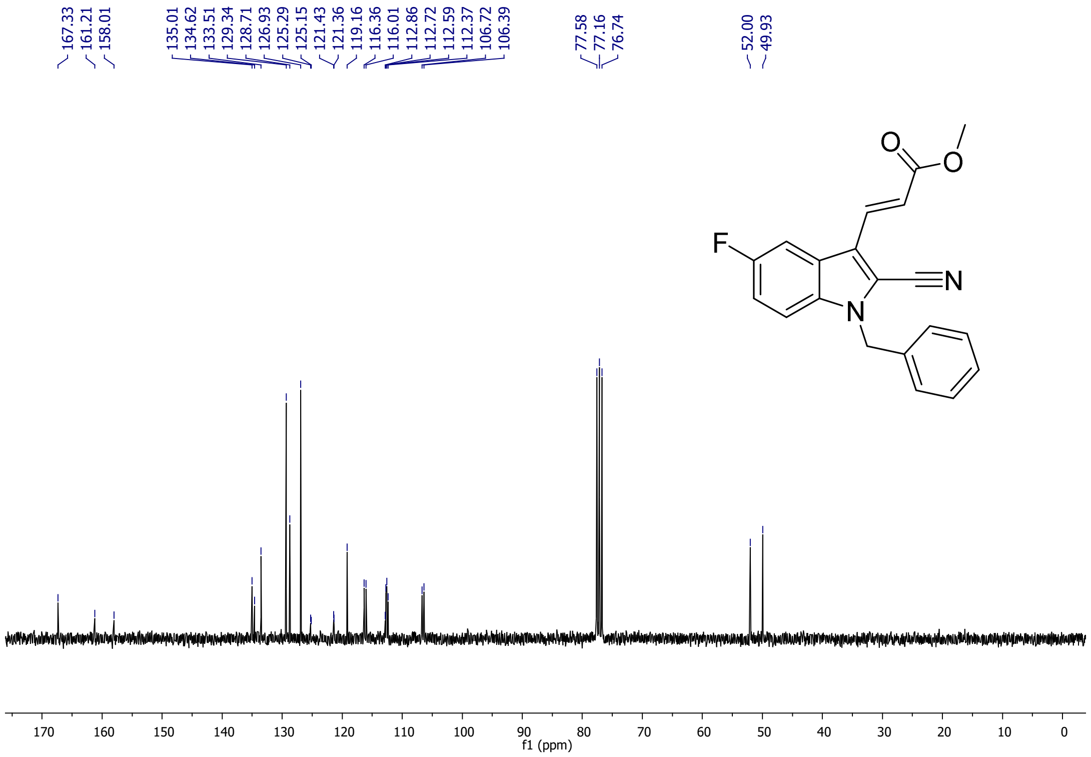
167.33
161.21
158.01
135.01
134.62
133.51
129.34
128.71
126.93
125.29
125.15
121.43
121.36
119.16
116.36
116.01
112.86
112.72
112.59
112.37
106.72
106.39
77.58
77.16
76.74
52.00
49.93
F
O
O
N
N
170 160 150 140 130 120 110 100 90 80 70 60 50 40 30 20 10 0
f1 (ppm)

(*E*)-1-Benzyl-3-(3-methylbut-1-en-1-yl)-1*H*-indole-2-carbonitrile (11b)

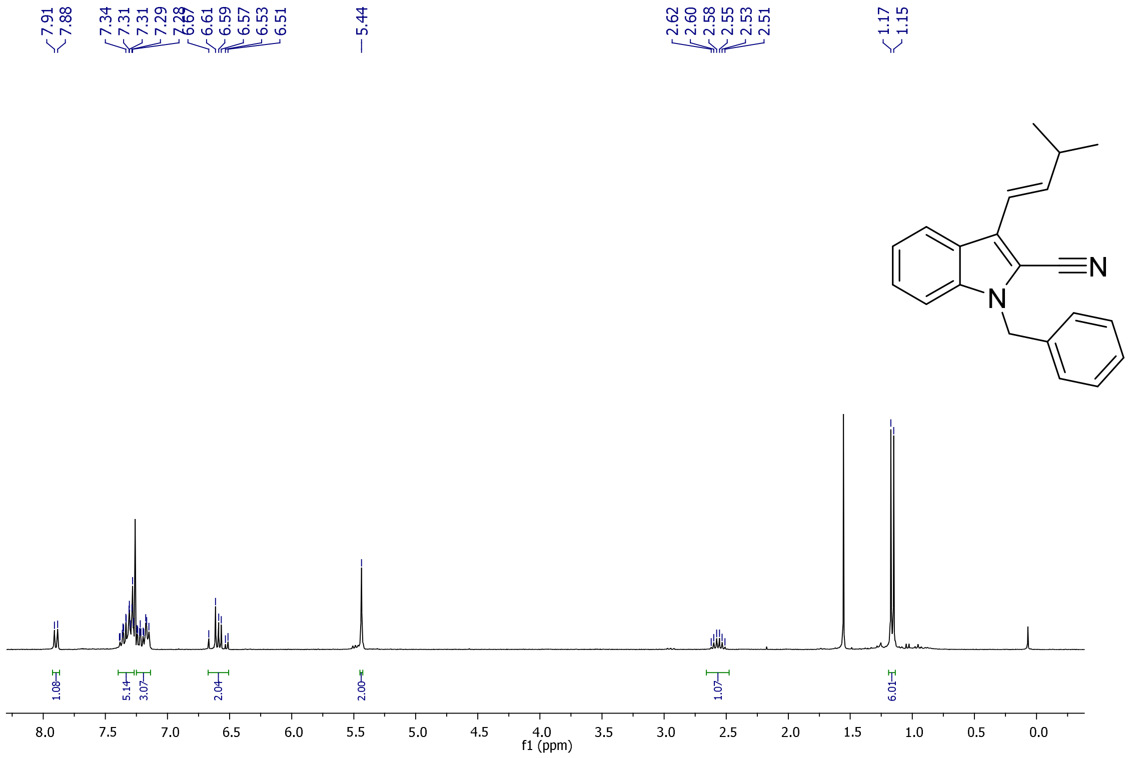

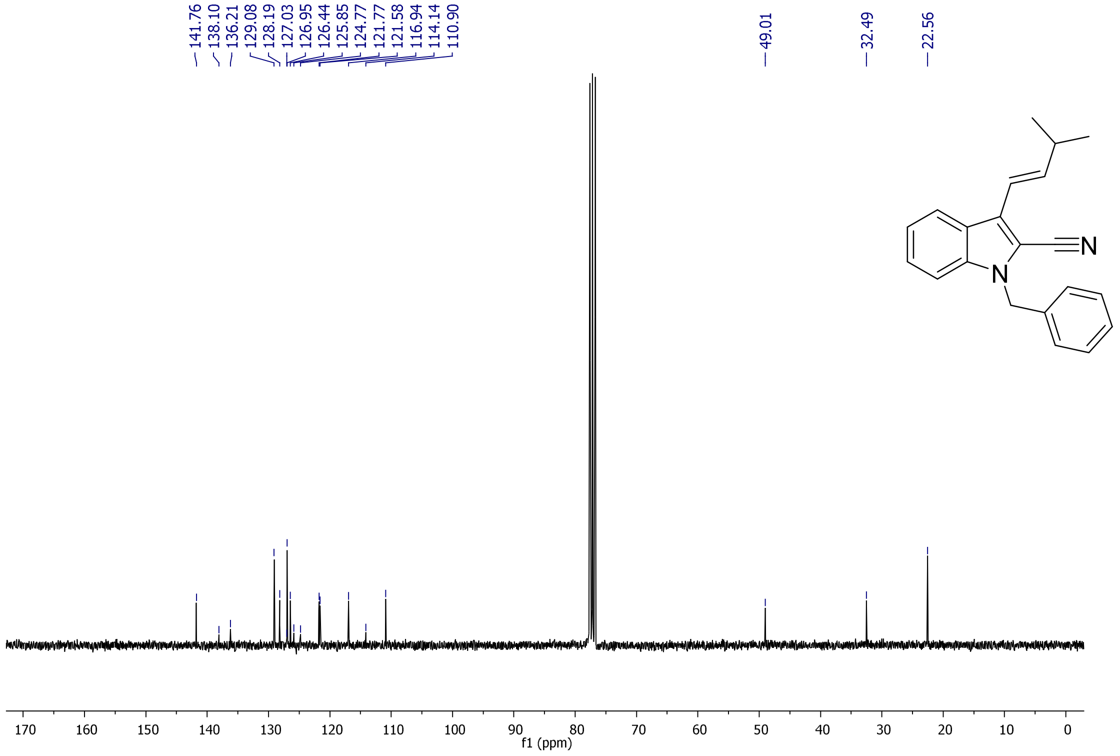

(*E*)-1-Benzyl-3-(2-(trimethylsilyl)vinyl)-1*H*-indole-2-carbonitrile (11c)

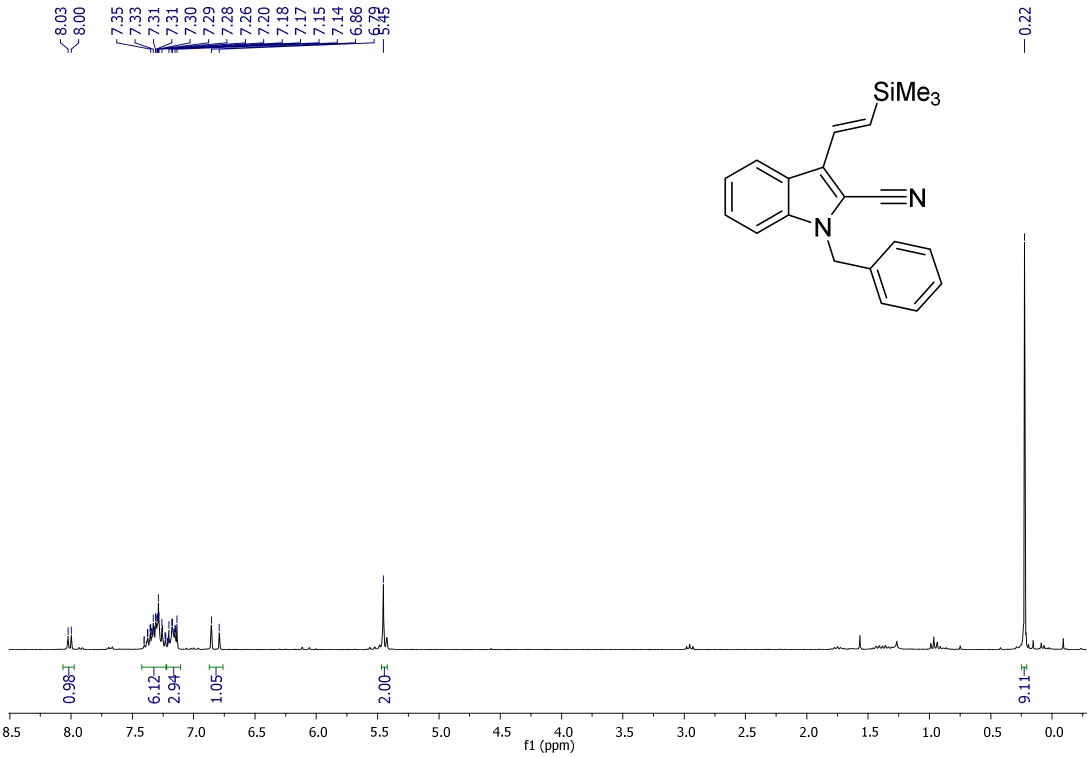

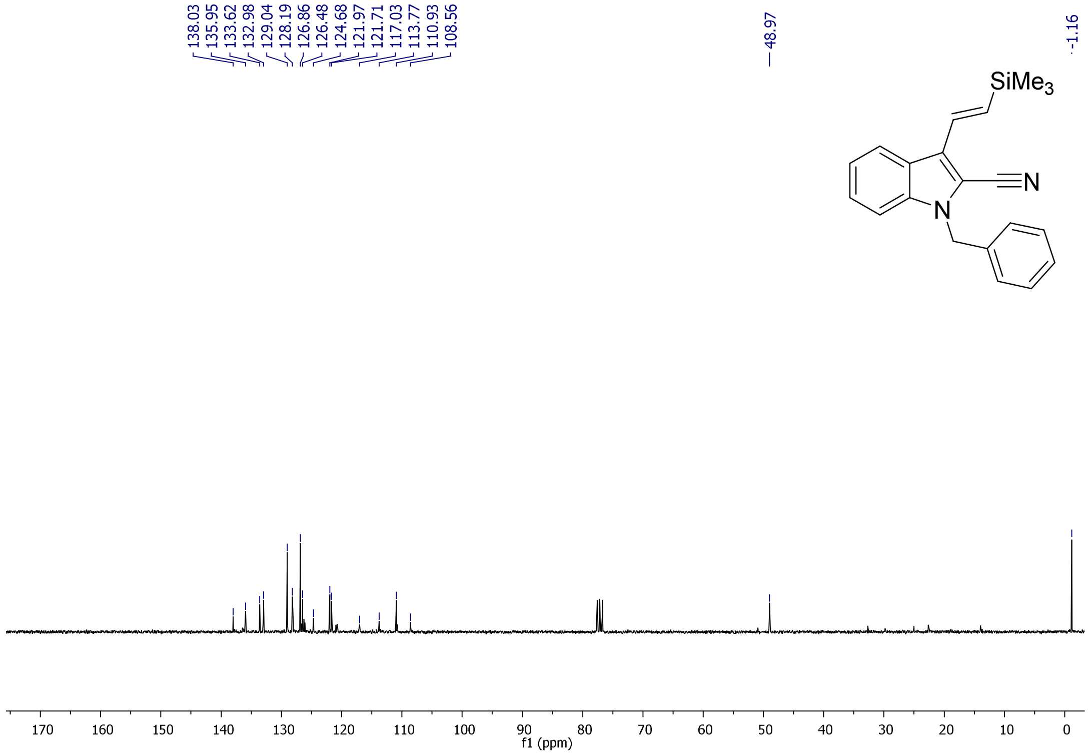

1-(1-Benzyl-3-(*p*-tolylethynyl)-1*H*-indol-2-yl)pentan-1-one (8b')

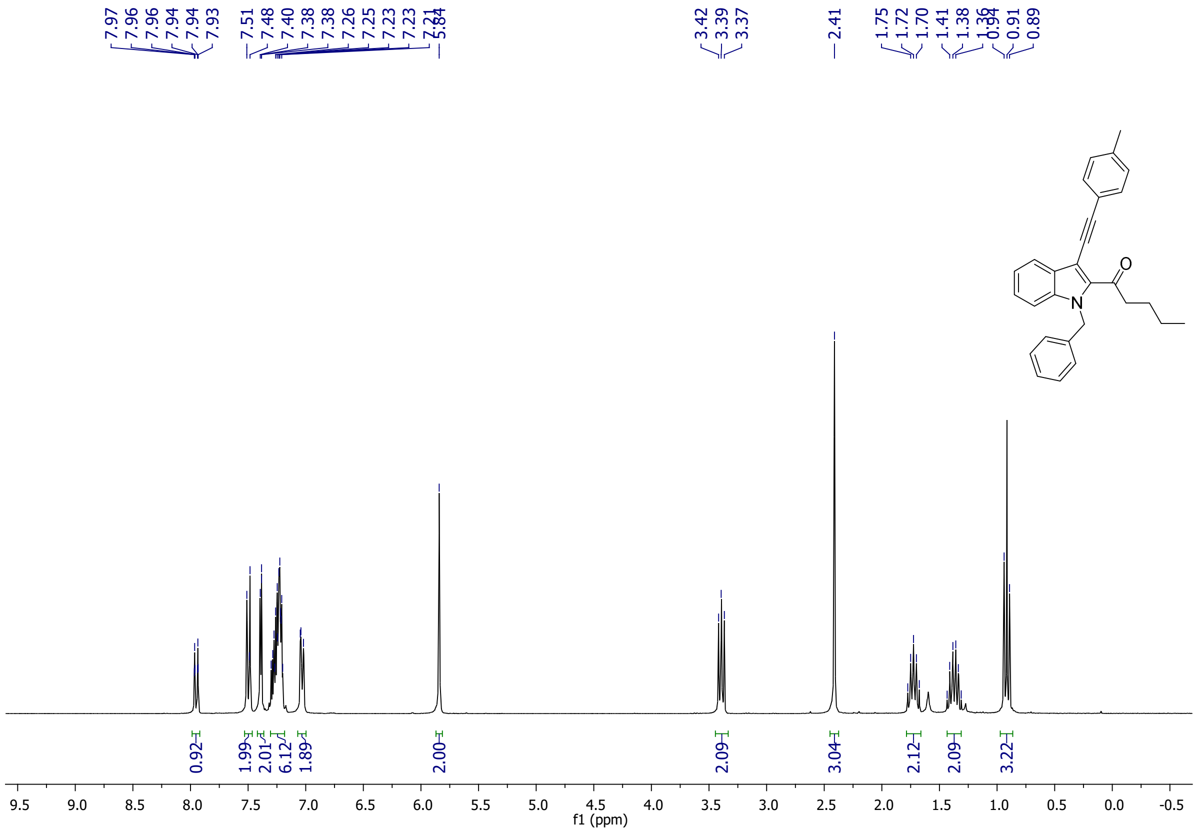

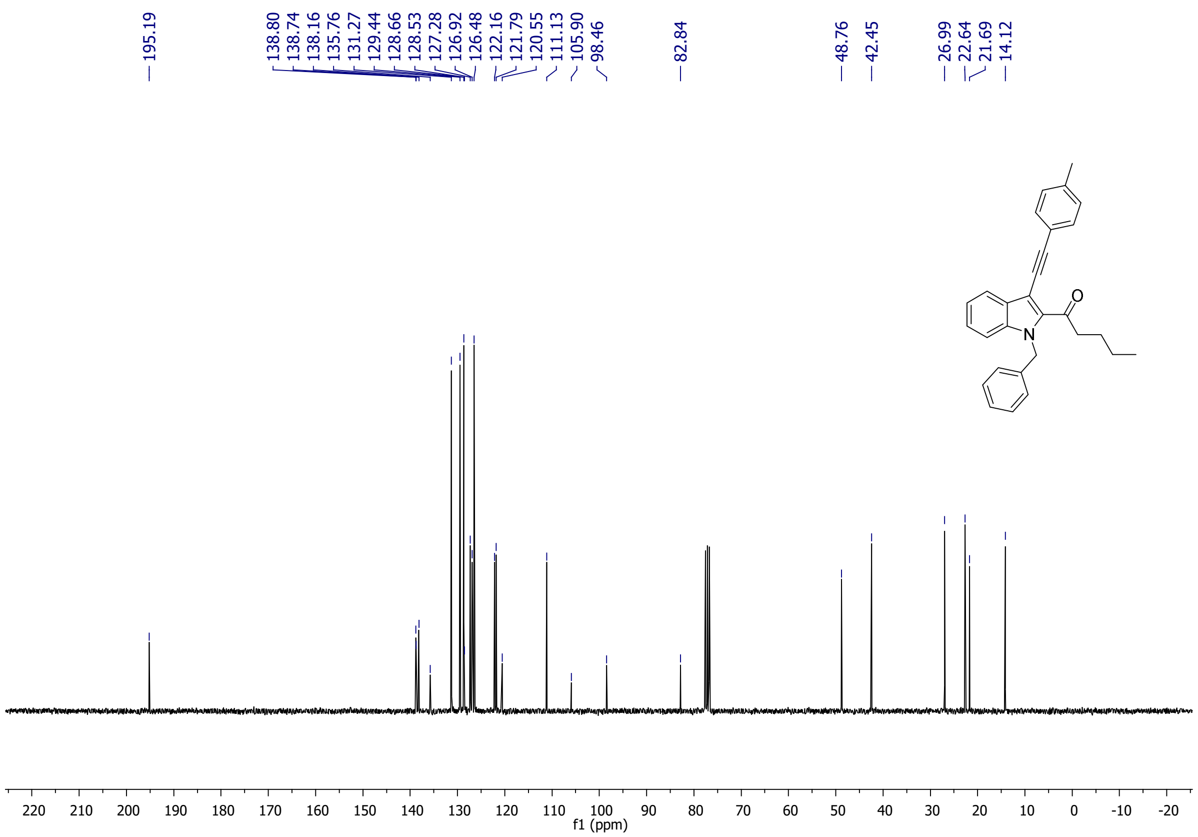

Supplement: Supplementary file 1 [file molecules-26-05287-s001.zip › molecules-1321463-supplementary.pdf]
